# Recruitment, Assembly, and Molecular Architecture of the SpoIIIE DNA Pump Revealed by Superresolution Microscopy

**DOI:** 10.1371/journal.pbio.1001557

**Published:** 2013-05-07

**Authors:** Jean-Bernard Fiche, Diego I. Cattoni, Nele Diekmann, Julio Mateos Langerak, Caroline Clerte, Catherine A. Royer, Emmanuel Margeat, Thierry Doan, Marcelo Nöllmann

**Affiliations:** 1 Centre National de la Recherche Scientifique, Unité Mixte de Recherche 5048, Centre de Biochimie Structurale, Montpellier, France; 2 Institut National de la Santé et la Recherche Médicale, Unité 1054, Montpellier, France; 3 Universités Montpellier I et II, Montpellier, France; 4 Centre National de la Recherche Scientifique, Institut de Génomique Humaine, Montpellier, France; 5 Laboratoire de Chimie Bactérienne, Centre National de la Recherche Scientifique Unité Mixte de Recherche 7283, Marseille, France; Harvard University, United States of America

## Abstract

ATP-fuelled molecular motors are responsible for rapid and specific transfer of double-stranded DNA during several fundamental processes, such as cell division, sporulation, bacterial conjugation, and viral DNA transport. A dramatic example of intercompartmental DNA transfer occurs during sporulation in *Bacillus subtilis*, in which two-thirds of a chromosome is transported across a division septum by the SpoIIIE ATPase. Here, we use photo-activated localization microscopy, structured illumination microscopy, and fluorescence fluctuation microscopy to investigate the mechanism of recruitment and assembly of the SpoIIIE pump and the molecular architecture of the DNA translocation complex. We find that SpoIIIE assembles into ∼45 nm complexes that are recruited to nascent sites of septation, and are subsequently escorted by the constriction machinery to the center of sporulation and division septa. SpoIIIE complexes contain 47±20 SpoIIIE molecules, a majority of which are assembled into hexamers. Finally, we show that directional DNA translocation leads to the establishment of a compartment-specific, asymmetric complex that exports DNA. Our data are inconsistent with the notion that SpoIIIE forms paired DNA conducting channels across fused membranes. Rather, our results support a model in which DNA translocation occurs through an aqueous DNA-conducting pore that could be structurally maintained by the divisional machinery, with SpoIIIE acting as a checkpoint preventing membrane fusion until completion of chromosome segregation. Our findings and proposed mechanism, and our unique combination of innovating methodologies, are relevant to the understanding of bacterial cell division, and may illuminate the mechanisms of other complex machineries involved in DNA conjugation and protein transport across membranes.

## Abstract 2

Super-resolution and fluctuation microscopy in a model DNA-segregation system reveal the architecture and assembly mechanism of the motor responsible for DNA translocation during bacterial cell division.

## Author Summary

Molecular motors are implicated in myriad cellular processes, notably in the transcription, replication, and segregation of DNA. Segregation or packaging of DNA is essential for production of viable viral particles, proper division of bacterial cells, and production of spores. A dramatic example of this process occurs during sporulation in *Bacillus subtilis*, in which a large proportion of the chromosome is actively transferred across a division septum by the SpoIIIE motor protein. Here, we use advanced microscopy methods to study the mechanism of recruitment and assembly of the SpoIIIE pump and the architecture of its complex with DNA. We found that SpoIIIE complexes are recruited before the beginning of cell division, and are subsequently escorted by the constriction machinery to the center of the septum. We show that the directionality of DNA transport by SpoIIIE results in the establishment of an asymmetric complex that exports DNA into the nascent spore. Our data are inconsistent with previous models that predicted the formation of a membrane-spanning DNA-conducting channel. Instead, the greater resolution afforded by our approach supports a model in which DNA movement occurs through an open pore structurally maintained by the division apparatus, with SpoIIIE working as a checkpoint preventing membrane fusion until completion of chromosome segregation. Our findings and proposed model may illuminate the mechanisms of other complex machineries involved in DNA conjugation and protein transport across membranes.

## Introduction

**Figure 1:**
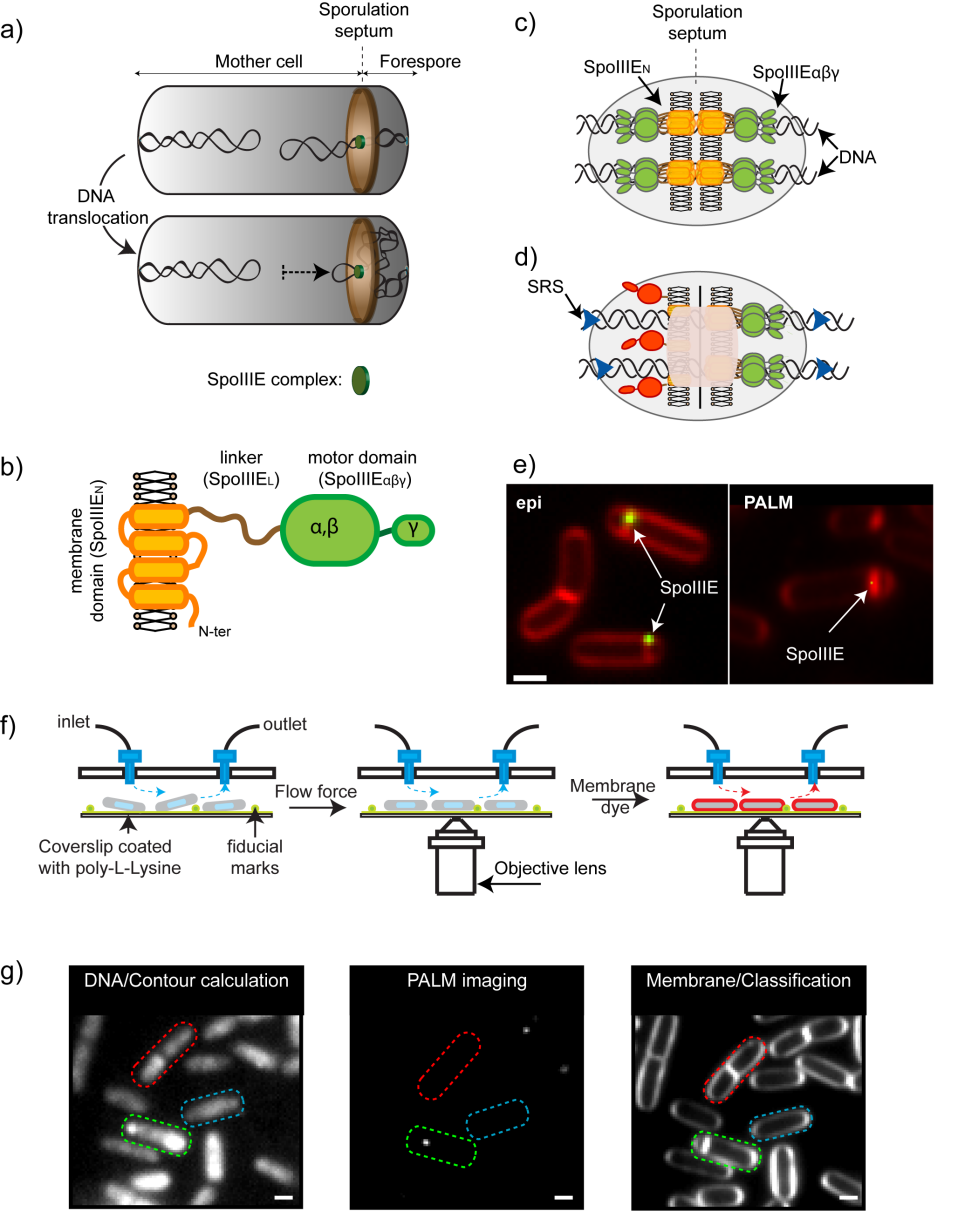
Role of SpoIIIE during chromosome segregation during sporulation in *B. subtilis* and experimental setup. (a) Formation of the asymmetric sporulation septum (brown disc) divides the cell into mother cell and forespore compartments, and traps one fourth of the chromosome (black ribbon) inside the forespore (upper panel). Packaging of the remainder of the chromosome into the nascent spore (lower panel) is achieved by SpoIIIE (green disc), a septal-bound double-stranded DNA motor of the FtsK family. (b) SpoIIIE is composed of a membrane-spanning domain (orange), a 134-residue unstructured linker (brown), and a motor domain responsible for directional DNA translocation (green). A single membrane bilayer is represented by yellow circles and black sticks. (c–d) Models for the architecture of the DNA translocating complex. (c) The DNA channel model suggested that SpoIIIE hexamers on either side of fused septa assemble to form a DNA-conducting channel. (d) The sequence-directed DNA exporter model proposed that specific interactions between SpoIIIE-γ and SRS sequences (blue arrows) lead to the establishment of active SpoIIIE hexamers (green) present exclusively on the mother cell side of the septum. Inactive SpoIIIE subunits are shown in red and are located in this model on the forespore side. (e) Left panel shows a conventional epi-fluorescence image of sporulating *B. subtilis* cells, in which SpoIIIE (green) assembles in diffraction-limited foci. Membranes are shown in red. Right panel shows a PALM image of a sporulating cell undergoing sporulation, in which the green spot represents a probability density reconstruction of the localization of SpoIIIE. Scale bar, 1 µm. (f) PALM acquisition procedure. A culture of *B. subtilis* pre-incubated with the DNA intercalator sytox-green and fiducial marks are introduced into a microfluidics device (left panel). Cells are flattened by using flow force and chromosomes are imaged by epi-fluorescence microscopy. SpoIIIE is imaged by PALM (middle panel), and finally a membrane dye is introduced and an epi-fluorescence image is obtained (right panel). (g) Cells were automatically detected and their contour (dotted lines) calculated from DNA images (left image). Individual SpoIIIE proteins were detected by PALM microscopy (middle panel), and membrane images used to classify bacteria according to their stage in the cell cycle (red, dividing; blue, vegetative/pre-divisional; green, sporulating). Middle panel shows the raw image of a single frame displaying two individual single-molecules. Scale bar, 1 µm.

Many cellular processes require the active transport of genetic material across hydrophobic membranes. In bacteria, these include chromosome segregation between daughter cells during cell division and transfer of DNA between separate cells during conjugation. A dramatic example of intercellular DNA transport is the segregation of chromosomes during sporulation in *Bacillus subtilis*. Early in this process, replicated sister chromosomes are remodeled into elongated axial filaments, and centromere-like elements located close to the replication origins of each chromosome are segregated and attached to opposite cell poles by the action of at least four proteins (Soj, Spo0J, RacA, and DivIVA) [1],[2]. Next, transcription of sporulation-specific factors directs the asymmetric relocation of the divisional plane from its normal mid-cell locus to the vicinity of the cell pole. This newly relocated septum divides the cell into mother cell and forespore and, importantly, traps a quarter of the chromosome inside the forespore compartment [3],[4]. Finally, SpoIIIE, a membrane-associated DNA translocase, assembles at the sporulation septum and translocates the remainder of the chromosome (∼3 Mbp) into the forespore compartment by using the energy of ATP hydrolysis (Figure 1a) [5],[6]. In addition to its function during sporulation, SpoIIIE may play a role in clearing DNA from division septa during vegetative growth [7]–[10].

SpoIIIE is closely related to the *Escherichia coli* DNA translocase FtsK, a septal-bound motor involved in chromosome dimer resolution (CDR) and in coordinating chromosome segregation and cell division [11]. SpoIIIE/FtsK are composed of three domains: an N-terminal, transmembrane-spanning domain (SpoIIIE_N_/FtsK_N_); a poorly conserved and putatively unstructured linker (SpoIIIE_L_/FtsK_L_); and a motor domain (SpoIIIE_αβγ_/FtsK_αβγ_) (Figure 1b). The motor domains of SpoIIIE and FtsK share ∼78% sequence similarity and have been classified as members of the RecA family of ATPases [12],[13]. Structural studies showed that the FtsK/SpoIIIE motor contains three subdomains (α, β, and γ) [14]. αβ assembles into a hexameric ring containing the ATPase machinery and a large central ∼3 nm channel through which double-stranded DNA (dsDNA) is threaded [14]. A critical feature of FtsK and SpoIIIE is their ability to translocate DNA directionally [6],[15], a process that requires the recognition of highly skewed octameric chromosomal DNA sequence motifs (KOPS, FtsK Orienting Polar Sequences; SRS, SpoIIIE Recognition Sequence) [6],[16],[17]. KOPS/SRS recognition is specifically achieved by FtsK/SpoIIIE γ domains, which are winged-helix domains linked to αβ by flexible linkers [6],[18]–[20]. Despite these advances, the mechanism of DNA translocation by SpoIIIE/FtsK in vivo is still poorly understood.

Early models for SpoIIIE, and more recent proposals for FtsK, suggested that SpoIIIE_N_/FtsK_N_ are anchored to a closing (but still unfused) septum, while motor domains assemble, bind, and directionally translocate the DNA segments crossing the aqueous channel connecting the two cells [11],[21],[22]. In stark contrast, the “DNA channel” model proposed that SpoIIIE_N_ (Figure 1b) assembles to form a DNA conducting channel consisting of two linked SpoIIIE hexamers of opposite orientation, spanning both lipid bilayers and the cell wall sandwiched between them (Figure 1c) [23]–[25]. Finally, it was suggested that DNA translocation and the relative orientation of chromosomal SRS/KOPS establish an asymmetric assembly of SpoIIIE_αβ_ motors on the mother-cell side of the septum (sequence-directed DNA exporter model) (Figure 1b and 1d) [6],[26]. This model requires DNA to be either pumped through an aqueous or a protein channel, but is at odds with the DNA channel model that requires SpoIIIE to be on either side of the septal membrane to assemble a paired DNA channel. To date, these models could not be refuted or confirmed by light microscopy methods because of the intrinsic resolution limit imposed by diffraction (∼250 nm) (Figure 1e, left panel).

In this article, we determine the position, architecture, and composition of SpoIIIE complexes in live cells by using photo-activation localization microscopy (PALM) [27],[28], a novel methodology that allows for the localization of genetically encoded photo-activatable markers at 10-fold higher resolution (∼20 nm) than conventional epifluorescence microscopy. To get insight into the cellular localization of SpoIIIE in three dimensions at superresolution and obtain absolute protein numbers and oligomerization states, we used two-color, 3D structured illumination microscopy (3D-SIM) [29], and scanning Number and Brightness analysis (N&B) [30]. We find that (1) SpoIIIE assembles into ∼45 nm clusters whose mobility and cellular localization are cell-cycle dependent; (2) in pre-divisional cells, SpoIIIE does not uniformly distribute on the cell membrane but preferentially localizes to future sites of septation; (3) SpoIIIE clusters are anchored to the leading edge of constricting septa, suggesting that assembly of a translocation proficient complex is not necessarily timed with DNA binding; (4) during cell division, SpoIIIE specifically assembles at the division septum, strongly suggesting a role in post-septational partitioning; (5) finally, we show that SpoIIIE complexes are symmetrically located on either side of the sporulation septum at the onset of DNA translocation, but ATP-fueled directional DNA transfer leads to the establishment of a biased, asymmetric complex specifically on the mother-cell side of the septum. Our data are inconsistent with the notion that SpoIIIE forms paired DNA conducting channels across fused membranes. Instead, they support a model in which DNA translocation occurs through an aqueous DNA-conducting pore structurally maintained by the divisional machinery, with SpoIIIE acting as a checkpoint preventing membrane fusion until completion of chromosome segregation.

## Results

### SpoIIIE Assembles in PALM-Limited Clusters

Previous studies using conventional microscopy methods proposed that SpoIIIE is monomeric during vegetative growth and only forms diffraction-limited foci during sporulation (Figure 1e) [24],[25],[31],[32], suggesting that SpoIIIE is recruited and assembles specifically at the sporulation septum. To test this hypothesis, we tagged SpoIIIE with a photo-activatable fluorescent protein and developed a microfluidics-coupled, multicolor PALM microscope to perform automatized superresolution detection and analysis of SpoIIIE localization and assembly in live cells (Figure 1f–g and Materials and Methods). First, a culture of *B. subtilis* was pre-incubated with the DNA intercalator sytox-green and introduced into a poly-L-lysine-coated microfluidics chamber (Figure 1f, left panel). Flow force (∼200 µl/s) was used to flatten and immobilize cells on the surface (Figure 1f, left and middle panels), and chromosomal DNA was imaged by epi-fluorescence microscopy (Figure 1g, left panel). Second, SpoIIIE was imaged by PALM microscopy (Figure 1f–g, middle panels). Third, the membrane dye FM4-64 was added into the micro-fluidics chamber and membranes were imaged by epi-fluorescence (Figure 1f–g, right panels). PALM and membrane images were sequentially detected using the same set of dichroic mirrors and emission filters to avoid chromatic aberrations. Fluorescence bleeding was also reduced, thanks to the sequential labeling procedure. Finally, custom-made software was used to automatically detect cells (Figure 1g, left panel), determine the localization of individual SpoIIIE proteins in the cell coordinate system by PALM microscopy (Figure 1g, middle panel), classify cells depending on their cell cycle state (Figure 1g, right panel), and quantify the amount of DNA in each cellular compartment (see Materials and Methods).

**Figure 2:**
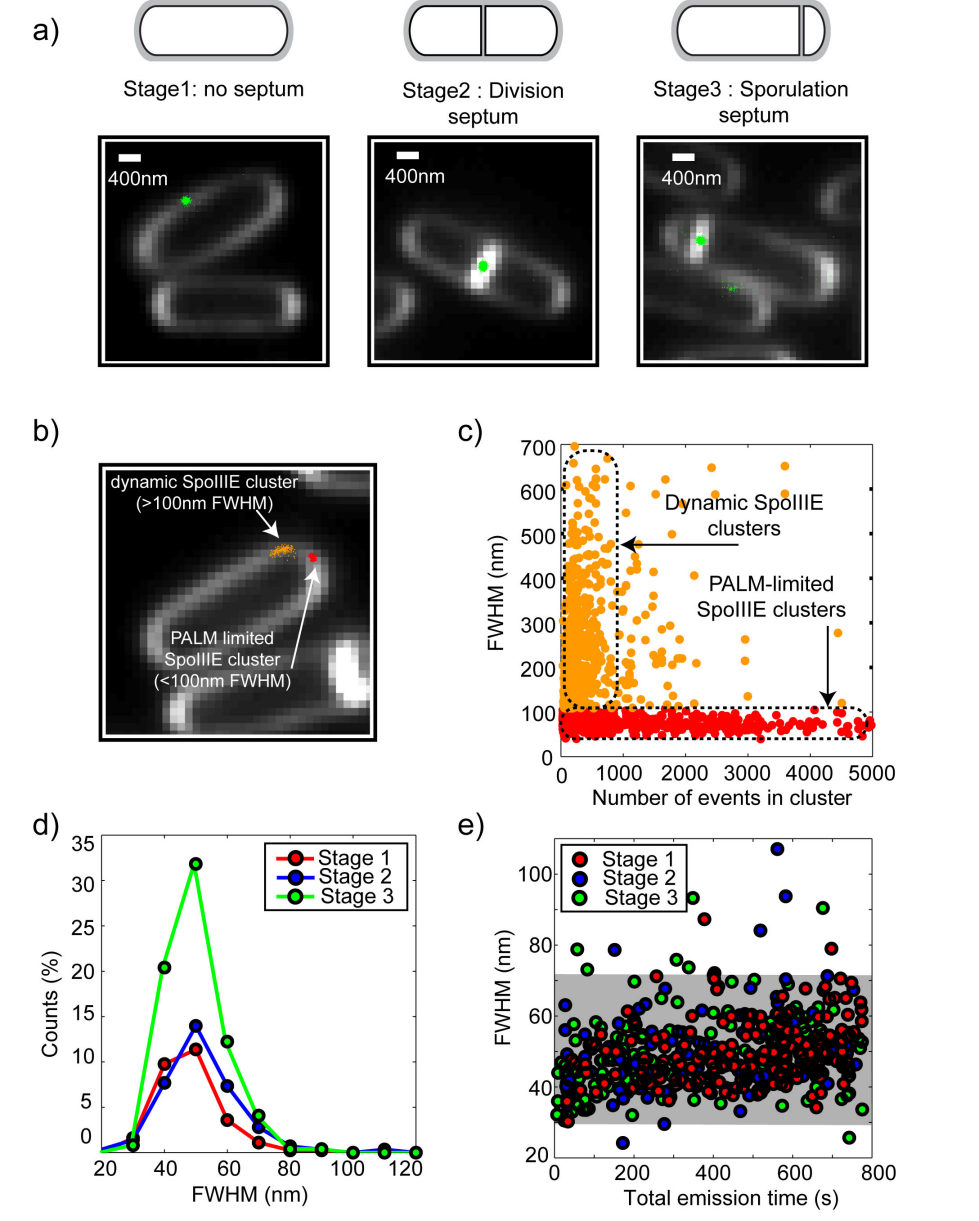
SpoIIIE localization at superresolution. (a) SpoIIIE is observed during all stages of the cell cycle. Individual cells were recognized and classified as described in Figure 1f. Pixel size was 110 nm. From each 55 ms image, we automatically determined the localization of each single molecule in the image by using MTT [57]. Each of these localizations is called a single-molecule event. In our pointilist representation, each single-molecule event is represented by a single green dot, whereas membrane stain is shown in white (see SM10 in Methods S1 for more details). Cells without septum were classified as stage 1 (vegetative/pre-divisional, left panel). Cells having a symmetric division septum were classified as stage 2 (division, middle panel), whereas those showing an asymmetric septum (at 1/5^th^ or 4/5^th^ of the total cell length) were classified as stage 3 (sporulating, right panel). (b) SpoIIIE clusters were automatically detected and classified depending on their size and composition. FWHM, full width at half maximum. (c) Analysis of the cluster size distribution versus the number of single-molecule events shows two distinct cluster types: PALM-limited clusters (red dots) have a size equal or smaller (∼45 nm FWHM) than the resolution of PALM in our conditions and contain a large number of events (>1,000), whilst dynamic clusters (orange dots) are large (>100 nm FWHM) and contain fewer events (<1,000). (d) The size of PALM-limited clusters is independent of cell cycle stage and the most typical size is ∼45 nm FWHM. (e) PALM-limited cluster sizes as a function of imaging time (total time used to image each single cluster) show that these clusters are extremely stable.

Using this approach, we first investigated the spatial distribution of SpoIIIE as a function of cell cycle stage in cells growing exponentially or after induction of sporulation. Three cell cycle stages were identified: cells were classified as in stage 1 or stage 2 (symmetric division) depending on whether a division septum at mid-cell could be detected or not (Figure 2a, left and middle panels), and in stage 3 (sporulating cells during DNA translocation) when septa were detected at the 1/5^th^ or 4/5^th^ position (Figure 2a, right panel). Stage 1 cells contained both vegetatively growing and pre-divisional cells, as we could not distinguish between these different physiological states solely based on membrane imaging. We observed that SpoIIIE forms clusters in all stages (Figure 2a). Clusters were automatically detected from the spatial distribution of single-molecule localization events (Figure 2b; SM5 and SM10 in Methods S1). Analysis of cluster sizes and compositions revealed that SpoIIIE assembles in two kinds of clusters (Figure 2b–c): the first type contains a large number of single-molecule events and displays sizes smaller than 100 nm (full width at half maximum, or FWHM), whereas the second type presents a considerably lower number of events and exhibits larger sizes (>100 nm FWHM). The assembly of SpoIIIE in these two cluster types is independent of the photo-activatable protein used (Figure S4d–e). Based on these properties, clusters were automatically classified as PALM-limited or dynamic, respectively (Figure 2b–c, Materials and Methods, and Methods S1). Interestingly, PALM-limited and dynamic clusters existed in all cell cycle stages (Figure 2a–b), but proportions between these cluster types varied considerably depending on cell cycle progression (see below).

Cluster size analysis showed that PALM-limited complexes are 45±10 nm in size (FWHM), independent of cell cycle stage (Figure 2d). Based on diffusion coefficients of typical membrane-bound proteins in bacteria (0.005–0.1 µm^2^/s) [33], we can estimate that free membrane diffusion of SpoIIIE would result in a root mean square displacement of at least ∼1 µm/min. Surprisingly, time-trace analysis of single clusters revealed that PALM-limited complexes are extremely stable (move less than ∼45 nm over several minutes), and their apparent size is not a function of imaging time (Figure 2e). In contrast, time-trace analysis showed that SpoIIIE in dynamic clusters is highly mobile (Figures S1 and S2). These observations suggest that SpoIIIE in PALM-limited clusters is tethered to large, rather immobile structures such as membrane-anchored division proteins or cytoskeletal components.

Analysis of the distribution of number of single events in PALM-limited and dynamic clusters (proportional to the number of SpoIIIE proteins) revealed small variations in cluster composition between cell cycle stages (Figure S3a). Interestingly, upon induction of sporulation, SpoIIIE levels determined by the number of single-molecule events in PALM-limited clusters increased ∼2-fold (Figure S3a–b). To validate these results and to obtain further insight into the nature of PALM-limited clusters, we turned to two-photon number and brightness microscopy, a method that allows for the in vivo quantification of absolute protein numbers and their oligomerization state (Methods S1) [30],[34]. After induction of sporulation, N&B experiments showed that clusters present in sporulation and division septa contain 47±20 SpoIIIE molecules, with brightness values consistent with at least 70% of SpoIIIE in hexameric state (i.e., 6–7 hexamers and ∼12 monomers, Figure S5). In contrast, clusters in division septa of exponentially growing cells displayed 20±4 SpoIIIE molecules, and showed brightness values compatible with at least 42% of SpoIIIE assembling into hexamers (i.e., 1–2 hexamers and ∼9 monomers, Figure S5). The *spoIIIE* gene is not transcribed from a sporulation-specific, but from a constitutive promoter, prompting the assumption that its expression levels may be constant during vegetative growth and sporulation [32],[35],[36]. In contrast, our PALM and N&B results show a ∼2.5-fold increase in the number of SpoIIIE molecules in clusters in cells undergoing sporulation, suggesting that SpoIIIE protein levels during vegetative growth are down-regulated, possibly by RNA degradation implicating the RNase Y pathway as for other sporulation-specific proteins (e.g., SpoIISAB) [37],[38].

Taken together, these results reveal that (1) SpoIIIE partially assembles into PALM-limited clusters during vegetative, division, and sporulation stages; (2) PALM-limited clusters are static and contain SpoIIIE in a partially hexamerized state; and (3) absolute SpoIIIE levels in clusters increase approximately 2-fold upon induction of sporulation as independently shown by PALM and N&B analysis.

### SpoIIIE Is Recruited and Assembles Before Septum Invagination and Follows the Leading Edge of Constricting Septa

**Figure 3:**
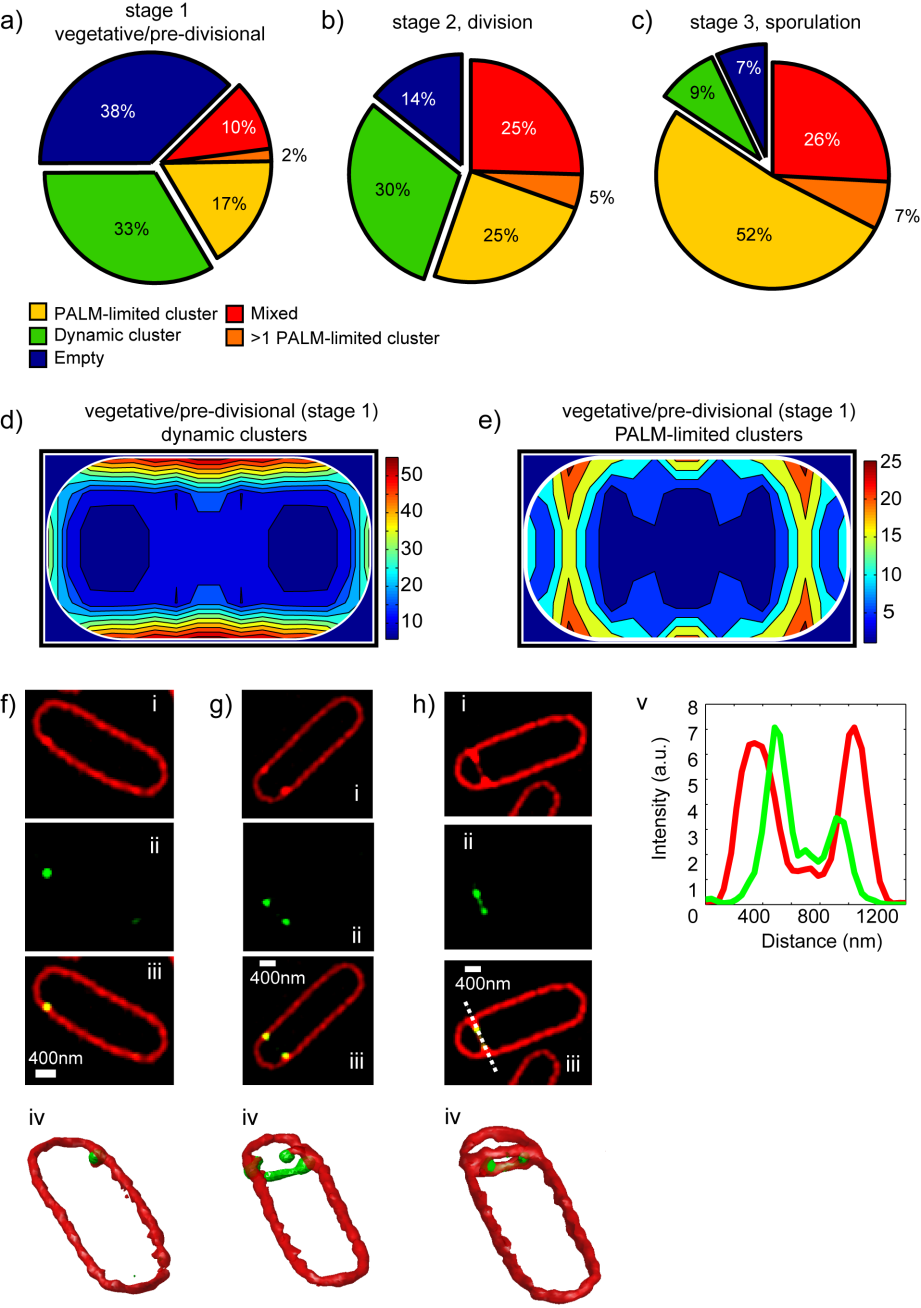
SpoIIIE is recruited to future sites of septation and localizes to the leading edge of closing septa. (a–c) Statistics of SpoIIIE clusters in vegetative/pre-divisional (*N* = 705), dividing (*N* = 174), and sporulating (*N* = 2 13) cells. Clusters were automatically classified as dynamic, PALM-limited, or mixed (cells containing both cluster types). Cells with less than 50 detected events were classified as “empty.” Cells with more than one PALM-limited cluster were classified independently from those containing a single one. The proportion of single PALM-limited clusters increases from vegetative/pre-divisional to dividing cells, and is maximal in sporulating cells. (d–e) The normalized coordinates of each localized event (axial and longitudinal coordinates) were used to calculate the localization probability distribution (heat maps) of SpoIIIE for each cluster type in vegetative/pre-divisional cells. Normalized localization probability distributions were calculated for the first quartile of the cell and then reflected into the other three quartiles to impose mirror symmetry in the axis perpendicular and parallel to the cell axis. The relative average number of clusters detected in each pixel of the grid is color-coded according to the color bar (right). White lines represent cells outlines. (d) Dynamic clusters distribute homogeneously over the cell membrane. (e) In contrast, in cells in which sporulation was induced, PALM-limited clusters specifically localize to future sites of asymmetric septation. (f–h) 3D-SIM imaging of *B. subtilis* cells in the early phases of sporulation at different stages of septal constriction. Axial projections showing the distribution of FM4-64-stained membranes (red, i), fluorescently labeled SpoIIIE (ii), and a merged image (iii). A 3D reconstruction is obtained by calculating a constant fluorescence intensity profile (iv). (f) SpoIIIE localizes to future sites of asymmetric division before the onset of septal constriction as a single cluster with a size <100 nm (resolution limit in 3D-SIM). (g) In cells early in septal constriction, SpoIIIE often shows a ring-like distribution probably due to the intrinsic dynamics of the closing septal ring. (h) In cells advanced in septum constriction, SpoIIIE specifically localizes to the leading edge of the invaginating septum. A line scan (v) of the fluorescence signal across the closing septum (white dotted line in panel iii) shows that SpoIIIE fluorescence (green) is always internal to the fluorescence of the membrane (red). Scale bar, 400 nm.

PALM-limited clusters appeared in all cell-cycle stages (Figure 3a–c), but their numbers and spatial localization patterns varied. In vegetative/pre-divisional cells (stage 1), ∼19% of cells displayed PALM-limited clusters, 33% dynamic clusters, and 10% a mixture of both, while in 38% of cells no cluster was detected (hereafter empty cells) (Figure 3a). The relative proportions of cluster types did not significantly depend on whether cells were exponentially growing or entering sporulation, or on the photo-activatable protein used (Figures S3, S4, and S6).

To investigate the cellular distribution of SpoIIIE, we constructed probability distribution maps (heat maps) based on the cellular localization statistics of thousands of individual molecules in single cells (Methods S1). This analysis showed that dynamic clusters distributed homogeneously over the cell membrane in vegetative/pre-divisional cells (Figure 3d). In contrast, and despite the absence of a visible septum, PALM-limited clusters localized to future sites of asymmetric septation in cells at the onset of sporulation (Figure 3e) and to future sites of symmetric division in exponentially growing cells (Figure S7a). Interestingly, both PALM-limited and dynamic clusters localized specifically to future septation sites with a higher frequency in long (pre-divisional) than in short (vegetative) cells (Figures S7e–h), suggesting that recruitment of SpoIIIE to future septation sites is timed with the assembly of a new septal ring.

Globally, these results suggest that during vegetative growth SpoIIIE can exist in two states: a mobile possibly pre-assembled state (dynamic clusters) with no defined localization, and a static, assembled state (PALM-limited clusters) with a well-defined localization to future sites of septation. It is tempting to speculate that the specific partitioning of SpoIIIE into these states and their specific cellular localization in pre-divisional cells are determined by the assembly of the division machinery, with PALM-limited clusters being recruited by specific interactions with components of the divisome.

To study the recruitment of SpoIIIE during the early steps of cell division, we turned to structured illumination microscopy (3D-SIM), a super-resolution method that allows for live, multicolor, three-dimensional imaging at a resolution of ∼100 nm [29]. Two-color images of *B. subtilis* membranes stained with FM4-64 and fluorescently labeled SpoIIIE were obtained in exponentially growing and sporulating cells. Independently of growth conditions, SpoIIIE localized to the site of nascent division and sporulation septa before mechanical constriction commenced (Figure 3f, Figure S8a, and Movie S1).

Later during septal constriction, SpoIIIE assembled in a single cluster in 38% of cells (Figure 3f), or in a ring that followed the leading edge of the closing septum in 57% of cells (Figure 3g, Figure S9a, and Movie S2), consistent with the dynamic nature of the closing septum. Interestingly, in this stage SpoIIIE clearly followed the leading edge of the invaginating septum in both asymmetric and symmetric septa (Figure 3g–h and Figure S8b–d), suggesting a direct interaction with the constriction machinery.

**Figure 4:**
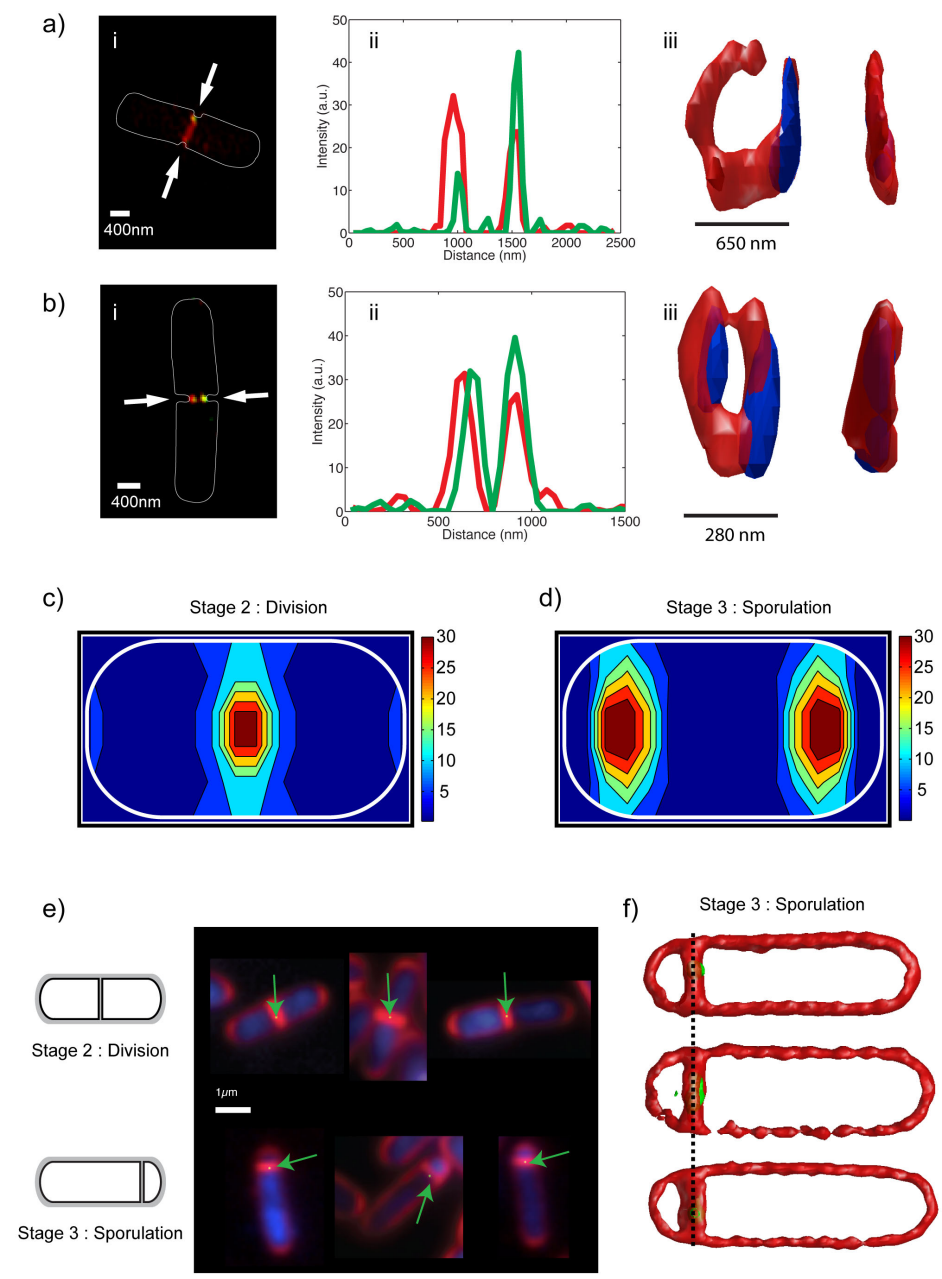
SpoIIIE clusters localize to the FtsZ ring in invaginating cells and to the septal midplane in dividing and sporulating cells. (a–b) (i) 3D-SIM imaging of SpoIIIE (green) and FtsZ (red) at two stages of septal constriction show that SpoIIIE clearly localizes to the FtsZ ring throughout the process of invagination (55% of cells, *N* = 78). Solid white line represents cell contour. (ii) A line scan of the fluorescence signal across the FtsZ ring (white arrows in panel i) shows that SpoIIIE fluorescence signal (green) overlaps to that of FtsZ (red). (iii) A 3D reconstruction of the FtsZ ring (red) and SpoIIIE (blue) is obtained by calculating a constant fluorescence intensity profile. Front (right) and side (left) views are shown. The size (FWHM) of the ring is 650 nm in (a-iii) and 280 nm in (b-iii). (c–d) Heat maps of PALM-limited clusters in (c) dividing (*N* = 87) and (d) sporulating (*N* = 180) cells show a clear SpoIIIE localization to the center of the septal plane. Normalized localization probability distributions were calculated for the first quartile of the cell and then reflected into the other three quartiles to impose mirror symmetry in the axis perpendicular and parallel to the cell axis. The relative average number of clusters detected in each pixel of the grid is color-coded according to the color bar (right). White lines represent cells outlines. (e) Probability density representation of SpoIIIE localization in dividing (top panels) and sporulating (bottom panel) cells. (f) Reconstructions of 3D-SIM images of three sporulating cells during DNA translocation show that SpoIIIE clusters localize to the center of the asymmetric septal plane.

To further investigate the role of the division apparatus in determining the localization of SpoIIIE, we simultaneously imaged SpoIIIE and FtsZ by 3D-SIM imaging. We observed that in the absence of any apparent FtsZ ring (vegetative cells), SpoIIIE did not seem to have any specific localization pattern (75% of cells, *N* = 104, Figure S10b). In contrast, in dividing cells, SpoIIIE clearly localized to early forming and actively constricting FtsZ-rings independently of their size (55% of cells, *N* = 78, Figure 4a–b, and Figure S10d–e). These results strongly suggest that SpoIIIE is an early component of the division machinery whose localization is driven by the assembly and constriction of the FtsZ ring.

### SpoIIIE Assembles in Single PALM-Limited Clusters in Mature Septa

Next, we investigated the assembly of SpoIIIE in mature division and sporulation septa (flat septa with no detectable opening). In cells undergoing symmetric division, the majority of cells (Figure 3b) contained single PALM-limited clusters that strongly localized to the center of the septal plane (Figure 4c,e and Figure S4d,g; see Figure S7b for dynamic clusters). These results are in agreement with 3D-SIM experiments in which a majority of dividing cells contained single SpoIIIE clusters at the center of the septal plane (Figure S9b). Interestingly, simultaneous 3D-SIM imaging of SpoIIIE and FtsZ showed that SpoIIIE often localized to the center of the septum after completion of constriction by the FtsZ ring (25% of cells, *N* = 104, Figure S10a, c–i), and tended to remain bound to the interphase between cells after separation (30%, *N* = 77, Figure S10a and S10c-ii).

In mature sporulation septa, 3D-SIM and PALM imaging showed that 85% of cells (Figure S9c, and Figure 3c) contained single SpoIIIE clusters that preferentially localized to the center of sporulation septa during DNA translocation (Figure 4d–f, Figures S4h and S7c, and Movie S3; see localization of dynamic clusters in Figure S7c). Importantly, PALM-limited clusters detected in flat sporulation septa are single (Figure 4e). As both chromosomal arms are independently translocated during sporulation [24], these data imply that the SpoIIIE motors (minimally one hexamer per DNA arm, ∼12 nm in size [14]) responsible for the translocation of each chromosome arm reside within a single PALM-limited cluster (∼45 nm in size) predominantly located at the septal midpoint.

Overall, these results are consistent with the notion that SpoIIIE complexes reach a mid-septum position following early interactions with components of the septal constriction machinery. In this scenario, the site of DNA translocation at the center of the septal disc is determined by the cylindrical symmetry of the septum.

### DNA-Translocating SpoIIIE Assembles Specifically on the Mother Cell

**Figure 5:**
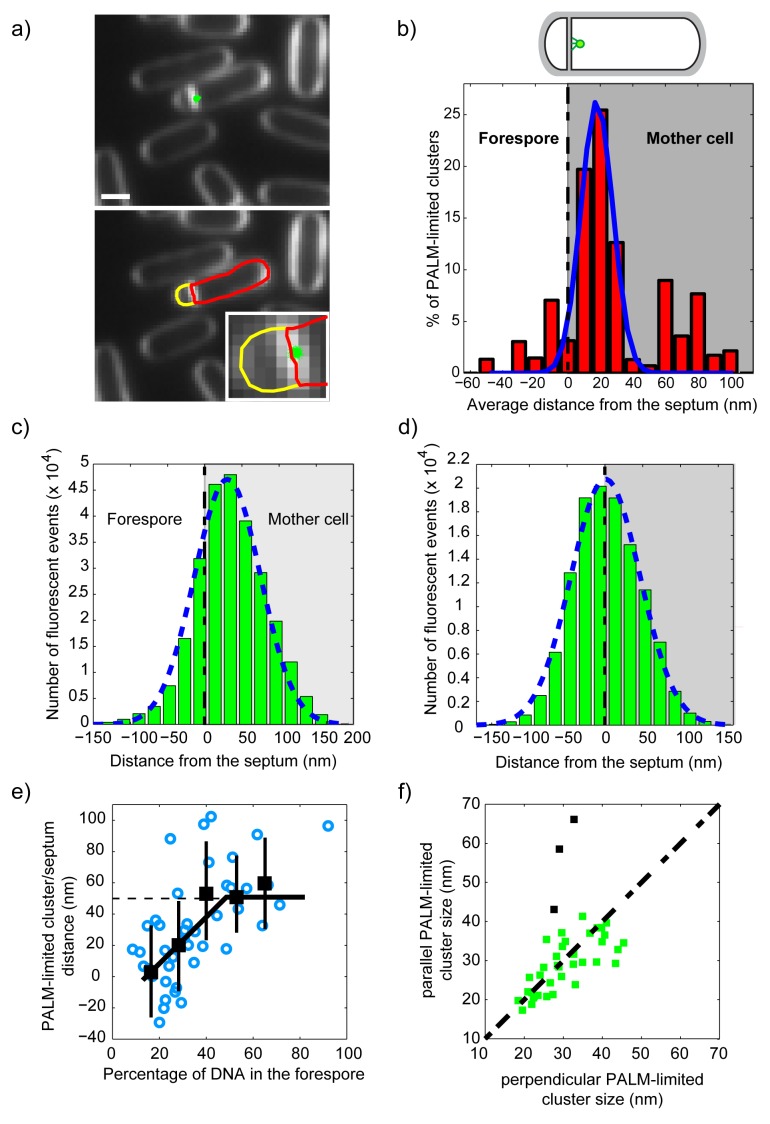
SpoIIIE clusters assemble asymmetrically in sporulation septa. (a) PALM imaging of SpoIIIE (green dots) in sporulating cells overlaid with an epi-fluorescence image of the membrane (white, top panel). Intensity profiles across the direction perpendicular to the septum were used to determine the precise localization of the septal plane, which was used to calculate the distance of each single-molecule detection to the center of the septum and to automatically partition the cell into forespore (yellow) and mother cell (red). Using this partition, individual PALM-limited clusters and single-molecule events were classified as belonging to the mother cell or the forespore compartments. (b) Histogram of PALM-limited cluster localizations with respect to the center of the septum (red columns) in sporulating cells with flat septa and undergoing DNA translocation (*N* = 43). Black dotted line indicates the position of the septum. A Gaussian distribution was fitted to the data (blue solid line). SpoIIIE PALM-limited clusters preferentially localize on the mother cell side of the sporulation septum. (c) Histogram of single-molecule localizations in PALM-limited clusters (*N* = 43) in sporulating cells undergoing DNA translocation, and Gaussian fit (blue dotted line). In sporulating cells, the distribution of SpoIIIE with respect to the septum is asymmetric and biased towards the direction of the mother cell. (d) Histogram of localizations of single molecules in PALM-limited clusters of dividing cells (*N* = 71) and Gaussian fit (blue dashed line). During division, the distribution of SpoIIIE with respect to the septum is symmetric and unbiased. (e) Distance of SpoIIIE PALM-limited clusters to the center of asymmetric septa versus the amount of translocated DNA. Open blue circles represent individual distance values for individual clusters, and black squares the average distance for all clusters detected at each particular percentage of DNA translocated. The relative distance increases linearly with the amount of DNA in the forespore until ∼45% of DNA translocated, and thereon remains constant at ∼50 nm. Solid black line is a guide to the eye. (f) The size (FWHM) of individual PALM-limited clusters in the directions parallel or perpendicular to the asymmetric septa were calculated and plotted against each other to evaluate cluster symmetry (squares). The overwhelming majority of clusters are symmetric (green squares) with only a small minority being longer in the direction parallel to the septum (black squares). The dotted line is a guide to the eye.

The DNA conducting channel and the sequence-directed DNA exporter models produce contrasting predictions: the former predicts that SpoIIIE is present in both sides of the sporulation septum [23],[24], while the latter predicts single symmetric clusters asymmetrically located on the mother cell side of the sporulation septum [6]. To test these distinct predictions, we investigated the relative localization of individual PALM-limited clusters with respect to the center of the septum, in symmetric and asymmetric septa. Electron-microscopy [24],[39] and 3D-SIM (Figure 4f) imaging demonstrated that sporulation and vegetative division septa are flat. In particular, electron-microscopy [24],[39] showed that sporulation septa have a thickness of only ∼10 nm. Thus, intensity profiles drawn across the direction perpendicular to the septum can be used to position the center of the septum with high precision (Figure 5a and Methods S1). We selected strictly flat and mature septa to avoid geometrical artifacts introduced by partially curved or incomplete septa appearing during engulfment or at the early stages of invagination. For each PALM-limited cluster, we determined their distance to the septum by projecting the vector defining their center-of-mass in the direction perpendicular to the septum (Methods S1).

Our results unequivocally show that the distribution of PALM-limited clusters is asymmetric and strongly biased towards the mother cell, with a maximum located at 22±2 nm (s.e.m.) (Figure 5b). These results were not influenced by the orientation of the cell axis in the sample plane (Figure S11a) or by the tilt angle between the cell axis and the sample plane (Figure S12). In addition to classifying the locations of the centers of each cluster, we determined the distribution of localization of single proteins as a function of their distance to the center of the septum. The distribution of individual SpoIIIE proteins was also biased and peaked at 25±1 nm (s.e.m.) (Figure 5c). A conservative classification of individual PALM-limited clusters based on their position with respect to the center of the septum similarly revealed that a majority of PALM-limited clusters are located on the mother cell side (49%) with only a small minority (∼6%) in the forespore (Figure S11b). To test whether these results were influenced by our cell immobilization methodology, we performed similar experiments in which cells were immobilized in agar-pads (Figure S11c). In agreement with our previous findings, we obtained a biased distribution with a maximum at 30±5 nm s.e.m. (Figure S11d). These statistics contrast with the symmetric distribution of single-molecules in PALM-limited clusters observed in division septa (Figure 5d and Figure S11b). This symmetric distribution is consistent with the lack of asymmetry between daughter cells. Overall, these results show that SpoIIIE assembles in a strongly compartment-specific manner during sporulation, in support of the sequence-directed DNA exporter model.

Next, we investigated whether the bias observed in SpoIIIE localization in sporulation septa depended on the amount of DNA translocated into the forespore. We used a method similar to that in Ptacin et al. [6], in which the percentage of DNA translocated is calculated from the ratio of DNA fluorescence inside the forespore with respect to that of the whole cell. This percentage starts at ∼15% when the septum closes and SpoIIIE first contacts DNA and increases constantly until reaching 100% when the full chromosome has been completely translocated [6]. Our experiments indicate that the average distance between SpoIIIE complexes and the septum increases linearly from ∼0±10 nm at the onset of DNA translocation, until reaching a maximum of 50±10 nm after ∼40% of the chromosome has been translocated (Figure 5e). These results strongly suggest that directional DNA translocation participates in the establishment of a biased SpoIIIE complex across the septum. In addition, these measurements show that SpoIIIE clusters are found in the forespore side of the septum (Figure 5b) only at the onset of DNA translocation.

Finally, we quantified the symmetry of individual PALM-limited clusters (aspect ratio) by measuring their dispersion in the direction parallel and perpendicular to the sporulation septum. The DNA exporter model predicts that SpoIIIE should assemble in single symmetric clusters, whereas the DNA conducting channel model predicts either two clusters on either side of the septum (which we do not observe) or a cluster elongated in the direction perpendicular to the septal plane (in case the clusters were unresolved). Our measurements clearly indicate that clusters are symmetric in shape with only a minority of clusters being more spread in the direction along the septum (Figure 5f). These results are in contradiction with predictions of the DNA conducting channel model, and reinforce the hypothesis by which PALM-limited clusters represent DNA-translocating SpoIIIE complexes that assemble in a compartment-specific fashion.

## Discussion

### Recruitment and Assembly of SpoIIIE

In this article, we combined for the first time, to our knowledge, micro-fluidics with three advanced microscopy approaches to obtain single-molecule, quantitative information at nanometer resolution, 3D superresolution cellular distributions of complexes, and quantitative measures of oligomerization state and absolute protein numbers. This approach is unique and will be relevant in the future to the study of macromolecular complexes and ultrastructures in bacteria or eukaryotes. Here, we used this methodology to investigate the recruitment, assembly, and architecture of the SpoIIIE-DNA translocation complex. Previously, it was thought that during vegetative growth SpoIIIE was monomeric and homogeneously distributed along the membrane [22],[24],[25],[31], with its assembly occurring specifically at the sporulation septum upon DNA binding [23]. Our results indicate that in vegetative cells SpoIIIE can be either dynamic or assemble in static, PALM-limited clusters that predominantly localize to future sites of septation. These results suggest that (1) recruitment of SpoIIIE to division/sporulation septa may not only involve the confluence of membrane-diffusive SpoIIIE monomers but also the relocalization of stable complexes to nascent septa, (2) the assembly of these complexes does not require interaction with DNA at the invaginating septum, and (3) SpoIIIE interacts with division proteins that direct its recruitment and localization.

Recent studies using TIRF microscopy showed that SpoIIIE monomers dynamically localize to the closing septum and proposed that SpoIIIE assembles stable complexes only upon contacting DNA [23]. Our PALM and 3D-SIM data show that SpoIIIE is able to assemble in static, PALM-limited clusters that localize to future sites of septation before the onset of invagination. In addition, 3D-SIM showed that during septal constriction, SpoIIIE clusters are dynamically driven by the leading edge of constricting septa, specifically localize to FtsZ rings, and come to lie at the septal midpoint in mature septa. This behavior can be attributed to the intrinsic dynamics of constricting septa. Interestingly, these observations did not depend on whether SpoIIIE was visualized in sporulation or division septa, suggesting that SpoIIIE localization depends on the same machinery implicated in both symmetric and asymmetric septation. Most of the components of the vegetative division machinery are involved in asymmetric cell division and are widely conserved throughout bacteria [40],[41]. In particular, tethering of SpoIIIE to membrane-bound components could involve interactions between SpoIIIE_N_, the domain required for septal recruitment [23],[25], and other components of the division machinery that localize to the Z-ring. In addition, SpoIIIE may interact with cytosolic components of the constriction apparatus (FtsZ, FtsA, ZapA) through SpoIIIE_L_, as recently shown for FtsK [42]. Further studies are required to determine whether SpoIIIE interacts with downstream membrane/periplasmic components of the division machinery involved in peptidoglycan synthesis–separation common to both vegetative and asymmetric septa (i.e., PBP1, DivIB, or FtsL), as was shown for *E. coli* FtsK [42],[43]. Overall, our results suggest that SpoIIIE takes part in both sporulation and division machineries, and that the different functions of SpoIIIE in chromosome dynamics during division and sporulation may be regulated by alternative interactions with factors specific to asymmetric or symmetric septa or by factors that are specifically located in the terminus region of the chromosome.

Previous studies using conventional microscopy methods reported that SpoIIIE forms foci on division septa at a very low frequency (∼1% of cells) [10]. These results can be explained by the reduced sensitivity of conventional microscopies, long acquisition times, decreased levels of SpoIIIE during exponential phase, and higher background fluorescence. Instead, our PALM, 3D-SIM, and N&B studies show that SpoIIIE is present in division septa in more than 30% of cells, localizes to the FtsZ ring during invagination in 55% of cells, remains at the center of constricted septa, and partially assembles into hexameric motors during vegetative cell division. These findings are consistent with previous observations showing that SpoIIIE plays two roles during vegetative cell division. First, it enhances the function of the chromosome dimer resolution (CDR) system (RipX/CodV) [9],[10]. Second, similar to other nonessential regulatory proteins involved in division, SpoIIIE is required for viability only when chromosome architecture is disrupted [7],[8]. In this background, SpoIIIE is required for repositioning chromosomes bisected by the invaginating septum during vegetative growth. In brief, our results support a role for SpoIIIE in actively transporting DNA during vegetative cell division, a function that could be essential in the presence of nucleoid partitioning defects produced by DNA damage or delays in replication/segregation. This function of SpoIIIE could be synergistically shared with SftA, the second FtsK analogue in *B. subtilis* that localizes to the division septum despite lacking a recognizable transmembrane domain [10]. Further research will be required to find what determines the apparently different functions of SpoIIIE in coordinating DNA segregation and cytokinesis in sporulation and vegetative septa.

### SpoIIIE Complex Polarity Is Determined by Directional Translocation

**Figure 6:**
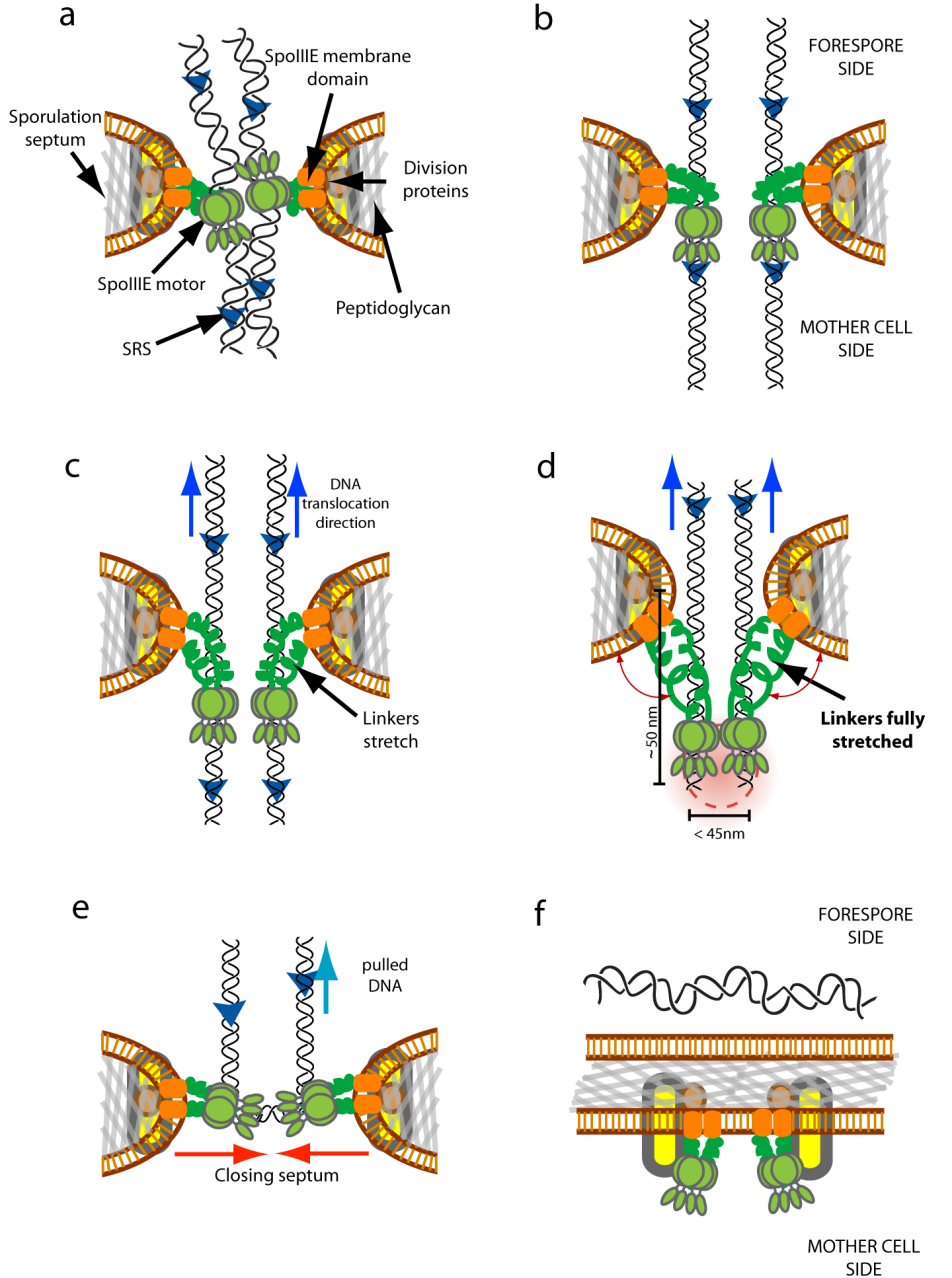
Model for the establishment and architecture of the DNA-translocating SpoIIIE complex. (a) SpoIIIE motors are recruited to the center of constricting sporulation septa and bind nonspecifically to DNA. Early divisional proteins interact with SpoIIIE membrane domains and contribute to the formation and regulation of the aqueous channel. (b) Interactions between SpoIIIE-γ and SRS lead to the establishment of directional motion towards the mother cell compartment. (c–d) The movement of SpoIIIE motors on DNA continues until the linker domains are fully stretched, at which point further translocation by SpoIIIE causes directional chromosomal segregation. Red gradient represents the size of a PALM-limited cluster. (e) Upon completion of DNA translocation, SpoIIIE motors disengage from DNA and the last segment of circular DNA is pulled into the forespore. (f) After completion of DNA translocation, SpoIIIE or a protein interacting with it leads to membrane fission.

Finally, our data showed that (1) at the onset of DNA translocation, SpoIIIE motors are predominantly localized at the center of the septum at equal distance from the forespore and mother cell; (2) subsequently, the bias in SpoIIIE motor localization towards the mother cell compartment increases linearly with the amount of DNA translocated, until reaching a maximum distance of ∼50 nm from the septal membrane on the mother cell side; and (3) at this stage, SpoIIIE clusters contain ∼50 molecules mostly in hexameric form, are extremely small (<45 nm), immobile, and symmetric, and are specifically located at the midpoint of the septal plane. These observations rule out models in which SpoIIIE alone forms a paired DNA-conducting channel or a sequence-directed DNA exporter, as these models require SpoIIIE to be present on both sides of the septum (Figure 1c–d). We cannot exclude the possibility that SpoIIIE assembles a heterologous paired channel comprised of SpoIIIE in the mother cell and an unidentified channel-forming protein in the forespore, or that an unidentified protein participates in the formation of a channel that conducts DNA from the mother cell to the forespore. However, the simplest model to explain our data involves SpoIIIE-driven translocation of DNA through a preexisting DNA-conducting pore before the final closure of the septum. In this model: (1) SpoIIIE motors localize to the center of the sporulation septum at the onset of DNA translocation after completion of septal constriction (Figure 6a) by interactions with components of the division machinery. (2) SpoIIIE follows the skew of chromosomal SRS sequences to establish directional translocation [6], which initially guides the movement of SpoIIIE motors away from the center of the septum towards the mother cell compartment (Figure 6b–c). (3) Movement of SpoIIIE on DNA continues until the linker domain is fully stretched, at which point further translocation by SpoIIIE causes directional chromosomal segregation. At this stage, we estimate the distance from the fluorescent probe to the center of the septal membrane by crystallographic and modeling data to be ∼40–55 nm (Figure 6d), consistent with our measured maximum distance of ∼50 nm. SpoIIIE_L_ was predicted to be an unstructured linker between the motor and the membrane anchoring domains of SpoIIIE. Our data suggest that SpoIIIE_L_ is fully extended during DNA translocation. Interestingly, bioinformatics analysis reveals that SpoIIIE_L_ is proline-rich and displays homology to the vinculin family of eukaryotic proteins involved in coupling integrins or cadherins to the actin cytoskeleton in focal adhesion complexes [44]. These P-rich proteins undergo a random coil to stretched conformational change when under tension, which regulates its interactions with actin filaments and thus serves as a force sensor [45]. We hypothesize that, similarly, the stretching of SpoIIIE_L_ could serve as a force sensor that communicates DNA translocation activity to other division proteins, thus acting as a checkpoint to prevent further steps in septum maturation (i.e., constriction, peptidoglycan degradation, or fusion) during DNA segregation (Figure 6d). This model provides an explanation why SpoIIIE septal localization requires trapped DNA [22], as in its absence the septum can fuse and the division machinery disassemble. Similarly, FtsK_L_ interacts with different components of the divisional machinery (FtsZ, FtsQ, FtsL, and FtsI) and was proposed to play a role in the control of constriction by a mechanism in which DNA translocation induces the breakage of FtsK_L_ contacts with its septal partners [42] and regulates peptidoglycan synthesis [46]. Alternatively, the septum could close slowly during DNA translocation, ending in a nonpermeable protein channel only at the last stages of chromosome translocation. In this model, the distance of SpoIIIE to the center of the septum increases progressively in the long axis of the cell as the channel diameter decreases. Further experiments will be required to investigate this model.

(4) During active DNA translocation, each chromosomal arm is being independently translocated by at least one SpoIIIE motor [24]. From our measurements of size, oligomerization state, and septal plane localization of PALM-limited clusters, we can conclude that SpoIIIE motors translocating each arm lie within ∼10–20 nm from each other. These observations are consistent with both chromosomal arms being pumped across the same preexisting channel (Figure 6d), instead of through two independent paired SpoIIIE channels with uncorrelated and homogeneous septal-plane localization, as predicted by the DNA channel model [24]. Furthermore, the size of SpoIIIE PALM-limited clusters and the length of SpoIIIE_L_ impose a constraint on the maximum size of the DNA-conducting pore (∼10–20 nm), whose architecture could be defined and maintained by components of the division machinery (Figure 6d). In fact, FtsW and FtsL are present at the septal midpoint in sporulating cells undergoing DNA translocation (flat septa) [24], but further research will be required to determine which division factors may be responsible for the structural integrity of the DNA-conducting pore. (5) Finally, SpoIIIE disassembles after completion of DNA translocation, and the last segment of circular DNA can be spontaneously drawn up into the forespore by entropic or condensing forces (Figure 6e) [47]. Either SpoIIIE_N_ or a factor that requires interactions with SpoIIIE_N_ is then responsible for septal membrane fusion after completion of DNA translocation (Figure 6f) [25]. Thus, SpoIIIE would act as a signaling checkpoint preventing membrane fission until the completion of DNA translocation.

Our model, in which SpoIIIE translocates DNA across an aqueous pore before septal fusion, is consistent with several previous observations. First, it was shown that assembly of a septal SpoIIIE focus and DNA binding (not translocation) by SpoIIIE motors are necessary to block diffusion of cytosolic proteins between mother cell and forespore compartments [25], compatible with a nanometer-sized channel obstructed by DNA and translocating SpoIIIE motors. Thus, SpoIIIE ensures separate, compartment-specific transcriptional programs by providing a diffusion barrier preventing transcription factors from moving between cells during DNA translocation [31],[48],[49] as well as by wire-stripping DNA [50].

Secondly, it was found that mutations in the glycine motif (GGGxxG) of SpoIIIE_N_ are proficient at focus formation and DNA translocation, but allow diffusion of proteins from the mother cell to the forespore [25]. Importantly, these results indicate that membrane fusion is not necessary for DNA translocation, as required by the DNA channel model, and could indicate that the GGGxxG motif is implicated in the recruitment of downstream components of the division machinery necessary for the proper architecture of the DNA-conducting pore (most likely components of the peptidoglycan synthesis/separation machinery).

Finally, membrane dye diffusion between mother cell and forespore is hindered by the presence of SpoIIIE foci at the septal membrane [24],[25], consistent with membrane dye diffusion being limited by the small size of the DNA-conducting pore [23] and the overcrowding effect of more than a dozen division machinery components [51] further hindering dye diffusion. Interestingly, further studies showed that membrane fusion occurs in the absence of SpoIIIE and DNA [23]. These results are important as they indicate that SpoIIIE is not essential for septal membrane fusion, and support the idea that translocating SpoIIIE may act as a signaling checkpoint preventing membrane fission until the completion of DNA translocation, as suggested by our model.

The notion that the DNA-conducting pore is not formed by paired SpoIIIE_N_ channels across a fused membrane is further supported by three additional observations: (1) mother cell-specific expression of SpoIIIE after septation rescues viable spore production to near wild-type levels [32], (2) the transmembrane domain of the SpoIIIE homolog FtsK can be replaced with other transmembrane domains without affecting its function in chromosome dimer resolution [21], and (3) *Streptomyces* Tra proteins, highly homologous motors of the SpoIIIE/FtsK family involved in conjugative transfer [52], are only present in the donor cell side of the communicating cell wall and were proposed to use preexisting channels contacting mycelial tips to transfer dsDNA [52],[53].

In brief, SpoIIIE/FtsK are extremely modular proteins, in which each domain encodes a separate function: SpoIIIE_N_/FtsK_N_ is responsible for septal recruitment and interactions with downstream components of the divisome, SpoIIIE_L_/Ftsk_L_ may act as a flexible linker communicating information between the motor domain and proteins composing the DNA-conducting pore, SpoIIIEα/Ftskα is responsible for DNA translocation, SpoIIIEβ/Ftskβ contains the ATPase motifs energizing the motor, and SpoIIIEγ/Ftskγ provides motor directionality. Our model for the recruitment, assembly, and architecture of SpoIIIE provides a rationale for how the coordination and co-regulation of these specific activities could lead to the synchronization of DNA segregation and cell division. Our findings and proposed mechanism considerably advance our understanding of the function and mechanism of SpoIIIE, are relevant to further understand the coordination between chromosome segregation and cell division [21], and may illuminate the mechanisms of other complex machineries involved in DNA conjugation and protein transport across membranes [52],[54].

## Materials and Methods

### Strains and Cell Culture

Strains are derivatives of *B. subtilis* PY79 and were kindly provided by Eric Becker, Tinya Fleming, Kit Pogliano, and David Rudner (Methods S1, Table S3). Mutations were introduced by transformation with plasmids or genomic DNA as described in Fleming et al. [23] from strain EBS1380. The *SpoIIIE* gene was replaced at its ectopic location by a SpoIIIE-GFP, a SpoIIIE-eosFP, or a SpoIIIE-mMaple fusion under the endogenous promoter. Fusion strains no longer contained the wild-type copy of SpoIIIE and relied on the tagged protein for function. Fusions were fully functional, as cells producing the fusion protein were able to grow and sporulate as well as cells producing the unmodified DNA translocase. Cells were cultured overnight at 20°C on solid (plate) Luria-Bertani (LB) medium at room temperature complemented with 10 µg/ml kanamycin. From a single colony, serial dilutions were grown overnight at 30°C in LB20%. An exponentially growing dilution (OD∼0.3–0.5) was then diluted to OD∼0.05 in LB20% and incubated at 37°C. When reaching OD∼0.6, bacteria were centrifuged, pelleted, and resuspended in prewarmed sporulation medium (see SM3 in Methods S1) [6],[55]. Cells were incubated for 2 h in sporulation medium to obtain a population where most cells are in the DNA translocation phase of sporulation. When DNA staining was needed, 7.5 nM of sytox green (Invitrogen, France) was added directly to the sporulation medium. Exponentially growing cells were centrifuged, pelleted, and incubated for 20′ in sporulation medium just before imaging to reduce background autofluorescence. More information can be found in SM3 in Methods S1.

### Sample Preparation

Coverslips were cleaned and used to assemble microfluidics chambers as described in Methods S1. Each channel was filled with 0.01% (v/v) poly-L-Lysine and incubated for 5 min at room temperature, then rinsed sequentially with water and sporulation buffer. A bacterial resuspension containing 40 nm fluorescent beads (Invitrogen, France) used as fiducial marks for drift correction was injected and incubated onto the poly-L-lysine coated surface for 5 min. A high flow force (∼200 µL/s of sporulation buffer) was applied to flatten cells against the surface. This immobilization scheme minimized cell movement or changes in cell shape during cell division or engulfment. In addition, images were acquired at 19°C to slow cell movement and morphological changes. For each experiment, a new channel was used and measurements were performed within the first 15–20 min after injection of cells into the channel to avoid any possible surface-immobilization effect. More information can be found in Methods S1.

### PALM Instrumentation and Imaging

Imaging was performed in a modified Nikon Eclipse Ti-S inverted microscope equipped with a 100× Plan-Apo oil-immersion objective (NA = 1.4) mounted on a closed-loop piezoelectric stage (Table S1). Four lasers with excitation wavelengths of 405 nm, 488 nm, 532 nm, and 641 nm were combined by a series of dichroic mirrors and achromatic lenses and delivered to the microscope. Dichroic mirrors (see Table S2) were used to reflect and filter excitation/photo-activation lasers and an emCCD camera (Andor Ixon 897, Ireland) used for detection. Pixel size was 110 nm. Acquisition software controlling lasers, filter wheels, and camera were homemade using LabView 2010 (National Instruments, France). An active feedback autofocus system based on the real-time monitoring of changes in intensity of a reflected, circularly polarized 1,064 nm IR beam was developed and locked the focal plane position to within 5 nm over hours (see Methods S1for further details).

Image collection was performed in three steps. First, sytox-stained DNA was imaged by exciting at 488 nm and detecting at 525 nm. Secondly, PALM imaging was performed by continuously acquiring between 1 and 2.5 10^4^ images at ∼20 Hz (55 ms per frame) under continuous illumination with the 532 nm read-out laser and by applying 405 nm pulses for photo-activation. The length and intensity of UV pulses were modified during the course of the experiment to maintain the density of activated fluorophores constant while ensuring that only one protein is activated at a time in a single diffraction-limited spot. Thirdly, bacterial membranes were imaged after injection of 100 µl of 10 nM FM4-64 in the microfluidics chamber and excitation at 532 nm. Imaging of protein and membrane were performed sequentially on the same detection channel (605/70 nm) to avoid chromatic aberrations. More information can be found in Methods S1.

### Automatized PALM Analysis

Bacteria were automatically detected on the DNA channel using a modified version of MicrobeTracker [56]. Single-molecule localizations were obtained by using MTT [57]. Bacterial contours, DNA and membrane images, and localization coordinates were further processed using PALMcbs, a homemade software written in Matlab (Mathworks, Inc.). Emission from fluorescent beads was used to correct for sample drift and chromatic aberrations between the DNA and the protein/membrane channels in order to obtain a well-corrected superposition between DNA, protein, and membrane images. Additionally, PALMcbs was used to automatically classify cells, detect the precise location of the septum, and analyze the distribution of localization events to automatically detect and classify SpoIIIE clusters. Then, for each individual cluster, we calculated its size, number of events, dynamics, cellular localization, and distance to the center of the septum or to the cell periphery. For cells displaying septa, the percentage of DNA in each compartment was calculated as the ratio of DNA fluorescence in each compartment versus the total integrated DNA fluorescence. Parameters used to acquire PALM data and further analysis details can be found in Methods S1.

### 3D Structured Illumination Microscopy and N&B analysis

Samples for 3D-SIM and N&B experiments were prepared as for PALM experiments (see above), but used a SpoIIIE-GFP fusion strain (Methods S1 and Table S3). 3D-SIM imaging was performed on an OMX V3 microscope (Applied Precision Inc) using 488 nm and 561 nm lasers and the corresponding standard drawer. Reconstruction and alignment of 3D-SIM images was performed using softWoRx v 5.0 (Applied Precision Inc.). We used 100 nm green and red fluorescent beads (Invitrogen, France) to measure the experimental optical transfer functions for the corresponding channels. 3D stacks of 170 nm TetraSpeck beads (Invitrogen, France) were used to measure x, y, and z offsets, rotation about the *z*-axis, and magnification differences between channels. These corrections are applied back into the acquired images. The measured resolution of our OMX microscope is 90 nm for GFP and 120 nm for FM4-64.

For N&B imaging, cells were imaged on an Axiovert 200 M inverted microscope (Zeiss, Germany) equipped with an ISS laser scanning module and an ISS Alba (ISS) with APD detection (SPCMAQR-15 Perkin Elmer). GFP was excited at 930 nm (25 mW) with a femtosecond pulsed infrared Titanium Sapphire laser (Spectra Physics MaiTai, Newport). A series of 50 raster scanned 20 µm×20 µm images (256×256 pixels) were recorded with a 100 µs laser dwell time per pixel. Image stacks were analyzed for number and brightness (N&B) values using SimFCS [30]. The pixel-based temporal average and variance images were calculated from 50 raster scans. The average brightness for a population of cells expressing monomeric freely diffusing GFP under the same set of conditions as those used for SpoIIIE imaging were obtained and used to normalize brightness. More details are provided in Methods S1.

## Supporting Information

Data S1 Document containing all supplementary figures, captions, and additional comments regarding supplementary figures.
(PDF)

Figure S1 Time-trace analysis of single PALM-limited and dynamic clusters. (a) A PALM reconstruction and a pointillist representation of a PALM-limited cluster in which single localizations are color-coded by time (complete time-series for that cluster). The position of early (blue) or late (red) localization events does not vary over time, indicating that the cluster is stationary during the acquisition time. (b) Number of events detected in the region of interest (ROI, in this case the whole cell) containing the PALM-limited cluster shown in (a) as a function of time. Single, nonoverlapping localization events are detected and dark times between events are longer than average emission times. These data are consistent with each photo-activatable protein being imaged until photo-bleached with no noticeable overlapping between events. In this case, the total number of emission bursts was 96, giving an estimate of ∼28 fluorescent proteins [58]. (c) Cumulative number of events detected as a function of time for the ROI in (a). From these data, it is clear that photo-activation rates are in average homogeneous during acquisition (dashed line is a guide to the eye). (d) A pointillist representation of a dynamic cluster in which single localizations are color-coded by time (complete time-series for that cluster). Here, localization events spread over several pixels and follow a path from the top to the bottom of the septum (see green arrow). (e) Number of events as a function of time detected in the cell represented in panel (d). Single, nonoverlapping localization events are detected and dark times between events are longer than average emission times. In this case, the total number of emission bursts was 37, giving an estimate of ∼10 fluorescent proteins [58]. (f) Cumulative number of events detected as a function of time for the ROI in (d). Photo-activation rates are in average homogeneous during acquisition (dashed line is a guide to the eye).
(EPS)

Figure S2 Mobile nature of dynamic clusters. (a) Pointillist reconstruction of single-molecule events (green) detected in a *B. subtilis* cell in stage 1. Single events inside the white dotted area were automatically classified as part of a dynamic cluster. (b) Representative trajectories generated from tracking the motion of single localizations identified in panel (a) (color coded by time of detection: red, green, and blue). The solid line is a guide to the eye. (c) Spatial and temporal evolution of dynamic clusters. Distance between the initial position of the first assigned location of the cluster and subsequent positions as a function of time. Dynamic and static subclusters are indicated with an arrow. Dashed line is a guide to the eye.
(EPS)

Figure S3 Probability distributions of number of single-molecule events detected in PALM-limited and dynamic clusters for sporulating and exponentially growing cells. N was calculated for each cluster type (PALM-limited (top) or dynamic (bottom)) in vegetative/pre-divisional, dividing, and sporulating cells. The N frequency distribution is plotted on the top histograms for PALM-limited clusters and on the bottom panels for dynamic clusters. In each plot, distributions for vegetative/pre-divisional (green), dividing (blue), and sporulating (red) cells are shown. A log-normal function was fitted to each N distribution in order to estimate the average number of events (<N>) for each type of clusters. (a) N distributions in cells after 2 h of sporulation induction. Here, N depends only weakly on cell stage, although clusters in sporulating cells tend to have higher numbers of molecules. In average, PALM-limited clusters possess ∼4 times more SpoIIIE molecules than dynamic clusters. (b) N distributions in exponentially growing cells. Under these conditions, N distributions did not depend strongly on cell cycle stage, however PALM-limited clusters contained only ∼2–3 times more proteins than dynamic clusters. Importantly, the overall number of proteins detected was independent on cluster type or cell cycle stage, and was ∼2.5-fold higher in sporulating than in exponentially growing cells. (c) Number of detected single-molecule events per cell (N_cell_), in cells after 2 h of sporulation induction. N_cell_ depends only weakly on cell stage, although sporulating cells tend to have higher numbers of molecules. There are large cell-to-cell variations in the number of events detected, possibly due to stochastic differences in SpoIIIE expression levels and due to a poorer detection of freely diffusing monomers under our acquisition conditions. More information can be found in Data S1.
(EPS)

Figure S4 SpoIIIE-mMaple assembles in PALM-limited clusters in all cell-cycle stages and localizes specifically to symmetric and asymmetric septa. (a–c) Statistics of SpoIIIE clusters in vegetative/pre-divisional (*N* = 279), dividing (*N* = 245), and sporulating (*N* = 118) cells using a SpoIIIE fusion to the photo-activatable protein mMaple [59]. Clusters were automatically classified as dynamic, PALM-limited, or mixed (cells containing both cluster types). Cells with less than 10 events were classified as “empty,” and cells in which all clusters had less than 25 events were also classified as “empty.” Cells with more than one PALM-limited cluster were classified independently from those containing a single one. SpoIIIE-mMaple PALM-limited clusters are present in considerable proportions in all cell-cycle stages. The proportions of empty cells are larger than those shown in Figure 3a–c due to the lower number of events detected for the mMaple fusion (cells with <10 events are considered empty and clusters with <25 events are discarded). The proportion of single PALM-limited clusters increases from vegetative/pre-divisional to dividing cells, and is maximal in sporulating cells (9% in vegetative/pre-divisional, 14% in dividing, and 26% in sporulating cells), a trend that is also observed for SpoIIIE-eosFP (17% in vegetative/pre-divisional, 25% in dividing, and 52% in sporulating cells). Importantly, the proportions between cells containing PALM-limited clusters versus those containing only dynamic clusters are maintained: (i) 62% for mMaple/45% for eosFP in vegetative/pre-divisional cells; (ii) 60% for mMaple/62% for eosFP in dividing cells; and (iii) 83% for mMaple/89% for eosFP in sporulating cells. (d) Pointillist representation of SpoIIIE-mMaple localization in dividing (top panels) and sporulating (bottom panel) cells show that SpoIIIE-mMaple PALM-limited clusters localize to the center of symmetric and asymmetric division septa. (e) Analysis of the cluster size distribution versus the number of mMaple single-molecule events shows two distinct cluster types: PALM-limited clusters (red dots) have a size equal or smaller (∼45 nm FWHM) than the resolution of PALM in our conditions and contain a large number of events (>300), whilst dynamic clusters (orange dots) are large (σ>45 nm equivalent to >100 nm FWHM) and contain fewer events (<300). Note that the ordinate shows the standard deviation of the cluster size, rather than the FWHM (with FWHM ∼2.2 σ). The difference between the mean number of events detected in dynamic and PALM-limited clusters in SpoIIIE-eos or SpoIIIE-mMaple reflect the different photo-physical behavior of these proteins. In both cases, there is a clear distinction between the number of events and the size of PALM-limited and dynamic clusters. (f–h) The normalized coordinates of each localized event (axial and longitudinal coordinates) were used to calculate the localization probability distribution (heat maps) of SpoIIIE for each cluster type in vegetative/pre-divisional, dividing, and sporulating cells. Normalized localization probability distributions were calculated for the first quartile of the cell and then reflected into the other three quartiles to impose mirror symmetry in the axis perpendicular and parallel to the cell axis. The relative average number of clusters detected in each pixel of the grid is color-coded according to the color bar (right). (f) SpoIIIE-mMaple dynamic clusters distribute homogeneously over the cell membrane. (g–h) In contrast, heat maps of PALM-limited clusters in (g) dividing (*N* = 245) and (d) sporulating (*N* = 118) cells show a clear SpoIIIE-mMaple localization to the center of the septal plane.
(EPS)

Figure S5 Number and Brightness analysis of exponential and sporulating cells undergoing cell division or sporulation. Number and Brightness analysis in exponentially growing or sporulating cells. Cells growing exponentially (a) or after induction of sporulation (b) were imaged by two-photon laser scanning microscopy (see Methods S1). The intensity fluctuations at each pixel in a series of rapid raster scanned images of bacteria are used to deconvolve the average intensity (counts/s) into the molecular brightness (counts/s/molecule) and absolute number of fluorescent proteins inside individual bacterial cells [30],[34]. Color-coded scale indicates the intensity level detected per excitation volume (0.07 fL inside a typical *B. subtilis* cell). Green and red arrows indicate the estimated position of division and sporulation septa, respectively. Brightness of monomeric GFP was obtained in a strain in which monomeric GFP was expressed in the cytosol (Methods S1) [34]. More information can be found in Data S1.
(EPS)

Figure S6 Distributions of PALM-limited, dynamic, and mixed clusters in sporulating and exponentially growing cells. (a) Sporulating (same as in Figure 3a–c) and (b) exponentially growing cells were imaged by PALM, and cells were classified as vegetative/pre-divisional, dividing, or sporulating (stages 1, 2, and 3, respectively). In each stage, SpoIIIE clusters distribution was statistically analyzed. The proportion of cells with no clusters detected (empty) in stage 1 and 2 was significantly higher for cells growing exponentially than in sporulating cells. However, the relative proportions of PALM-limited and dynamic clusters remained unchanged. In stage 3 cells, the total number of both types of PALM-limited clusters increased significantly with respect to cells in either stage 1 or 2. More information can be found in Data S1.
(EPS)

Figure S7 Heat maps representing the spatial probability distributions of PALM-limited and dynamic SpoIIIE clusters in vegetative/pre-divisional, dividing, and sporulating cells. Heat maps representing the localization statistics of individual PALM-limited and dynamic clusters in pre-divisional, division, and sporulating cells were built as described in Figure 3d and Methods S1. White lines represent cell outlines, and the relative density of clusters detected in each position is color-coded according to the color bar (right side of each figure). (a) PALM-limited clusters in exponentially growing cells (stage 1) are predominantly found at positions where a division septum would be expected during symmetric cell division. A very small proportion of PALM-limited clusters is also detected at future asymmetric sites. This observation is not unexpected since exponentially growing cells were incubated in sporulation medium for 20 min to reduce cytoplasmatic background fluorescence. (b–c) Dynamic clusters in a sporulating culture, for cells with a symmetric division septum (b) or an asymmetric sporulation septum (c), are mostly localized to the center of symmetric and asymmetric septa. Distributions are slightly larger (in the septal plane) than those observed for PALM-limited clusters (Figure 4c–d). In contrast to the strong localization of PALM-limited clusters to the center of the division and sporulation septa (Figure 4c–d), a small proportion of dynamic clusters can be detected along the cell wall and at the poles (left). (d) Distribution of cell lengths from exponentially growing cultures (blue bars), and Gaussian fit (green solid line) indicating an average size of 2.5±0.5 µm (s.d.). (e–f) Distribution of localization of PALM-limited clusters in cells with lengths smaller (e) or larger (f) than 3 µm. PALM-limited clusters localize to future sites of symmetric or asymmetric septation in longer cells (panel f, pre-divisional cells), but show a homogeneous localization in small cells (panel e, newly born or still vegetatively growing). These results are consistent with SpoIIIE preferably localizing to new division sites in pre-divisional but not in vegetatively growing cells. (g–h) Distribution of localization of dynamic clusters in cells with lengths (L) smaller (g) or larger (h) than 3 µm. As PALM-limited, dynamic clusters localize to future sites of symmetric septation in long cells (panel h, pre-divisional cells), but show a rather homogeneous localization pattern in small cells (panel g, newly born or still vegetatively growing). More information can be found in Data S1.
(EPS)

Figure S8 3D-SIM imaging of SpoIIIE during early and late stages of septum formation in cells undergoing symmetric division. (a) Localization of SpoIIIE in vegetative/pre-divisional (stage 1) cells from an exponentially growing culture. Single clusters of SpoIIIE localize to future sites of symmetric division (white arrow heads) before a septum can be detected. As shown in these 3D-SIM images, cells lie flat on the micro-fluidics chamber. (b–d) Localization of SpoIIIE-GFP at different stages of septum invagination during symmetric cell division in *B. subtilis* by 3D-SIM. Three stages of advancement of the septum formation are displayed: (b) nascent, (c) early septation, and (d) and closing septum. For each stage, the left panel represents a z-stack where the first image represents membrane (stained with FM4-64, red), the middle image shows the localization of SpoIIIE (green), and the last image displays the overlay of fluorescence from SpoIIIE and membrane. A 3D reconstruction displaying membrane (red) and SpoIIIE (green) fluorescence signals is shown in the middle panel. In these different stages, SpoIIIE distributes either in single clusters (b–c) or along an arc (d). The latter probably due to the intrinsic dynamical behavior of invaginating septa. Finally, the right panel shows the quantification of fluorescence intensity from the membrane (red solid line) and SpoIIIE (green solid line) along the septum. Combined to the 3D reconstructions, these curves clearly show that SpoIIIE follow the leading edge of the closing septum. Interestingly, at the onset of invagination SpoIIIE localizes with the septal membrane, but shifts to the leading edge as invagination progresses. The white dotted line in the right image of the left panel indicates the direction used to calculate intensity profiles shown in the panels on the right. Scale bar, 400 nm. More information can be found in Data S1.
(EPS)

Figure S9 Number of clusters detected in sporulating and exponentially growing cells by 3D-SIM. (a) 3D-SIM statistics for sporulating cells with incomplete asymmetric septa (undergoing constriction). SpoIIIE appears in both single and dynamic clusters (see Figure S8b–d). In dynamic clusters, SpoIIIE localizes mainly in arcs accompanying the leading edge of the invaginating septum (see Figure 3g iv, and Figure S8d), consistent with the intrinsic dynamics of the septal membrane during constriction. (b) 3D-SIM statistics for exponentially growing cells undergoing division and displaying a mature (symmetric) division septum (stage 2). Here, half the cells show a single cluster of SpoIIIE at the division septum. (c) 3D-SIM statistics for sporulating cells displaying a mature sporulating septum (stage 3). Here, SpoIIIE predominantly assembles in single clusters with a size smaller than the lateral resolution limit of 3D-SIM (<100 nm). More information can be found in Data S1.
(EPS)

Figure S10 SpoIIIE localizes to FtsZ rings in dividing cells. (a) Wide-field epi-fluorescence imaging of SpoIIIE (white) and FtsZ (red). A field of view displays cells in different cell cycle stages, with SpoIIIE assembling in clusters during (i) closure of the FtsZ septal ring, (ii) at the end of septation (FtsZ fluorescence becomes cytoplasmic), and (iii) after cell separation. 3D-SIM images were selected whenever possible, but the same results were obtained with wide-field epi-fluorescence imaging. (b) 3D-SIM imaging of SpoIIIE (green) and FtsZ (red). SpoIIIE loses its localization pattern in vegetative cells (i.e., small cells with no visible FtsZ ring) in 75% of cells (*N* = 104). (c) (i) Wide-field epi-fluorescence imaging of SpoIIIE (green) and FtsZ (red). SpoIIIE often assembles in a cluster at the end of septal constriction when FtsZ becomes cytosolic (30%, *N* = 104). Note that in the terminology employed here, a “closed septum” is a septum that has completed invagination but not necessarily fused membranes. (ii) 3D-SIM imaging of SpoIIIE (green) and membrane stain (red). SpoIIIE often remains localized to the septum after cell separation (30%, *N* = 77). 3D-SIM images were selected whenever possible, but the same results were obtained with wide-field epi-fluorescence imaging. (d) Montage of a 3D-SIM image of SpoIIIE (green) and FtsZ (red), with z representing different z-stacks separated by 130 nm. SpoIIIE localizes to the FtsZ-ring in early dividing cells (ring diameter ∼700 nm). 3D reconstruction and fluorescence line-scan are shown in Figure 4a. (e) Montage of a 3D-SIM image of SpoIIIE (green) and FtsZ (red), with z representing different z-stacks separated by 130 nm. SpoIIIE localizes to the FtsZ-ring in a cell that almost completed invagination (ring diameter ∼300 nm). 3D reconstruction and fluorescence line-scan are shown in Figure 4b.
(EPS)

Figure S11 Effects of cell orientation in the localization of SpoIIIE, conservative classification of PALM-limited cluster distributions, and single-molecule SpoIIIE localizations with respect to the center of sporulation septa in agar pads. (a) Schematic representation of the directions of long axes of sporulating bacteria accumulated in a single field of view from different PALM imaging experiments. For each single cell, an arrow is represented, its position representing the location of the cell in the field of view, its direction representing the long axis of the cell, and its orientation pointing toward the direction of the sporulation septum. The color of each arrow indicates the calculated position of a SpoIIIE cluster with respect to a sporulating septum (green for forespore, red for mother cell, and yellow undefined). The direction and location of arrows is uncorrelated with the septal localization of SpoIIIE clusters, indicating that the relative position of SpoIIIE clusters with respect to sporulation septa is independent of cell orientation or position in the field of view. (b) The position of PALM-limited clusters with respect to the center of dividing or sporulating septa was calculated as described in Methods S1. From these measurements, clusters were conservatively classified as in the mother cell compartment (distance >15 nm), central (−15>distance<15 nm), or in the forespore compartment (distance <−15 nm) (*N* = 43). For cells undergoing symmetric division, clusters were similarly classified as “central,” or on the “left,” or “right” compartments (*N* = 71). (c) Experimental setup used to immobilize cells (black cylinder) in agar pads (yellow). Fiducial marks (green spheres) were used to correct for lateral drift during acquisition and an IR laser to correct for axial drift in real-time. More details are provided in Methods S1. (d) Distribution of distances of single emitters (proportional to the number of single SpoIIIE proteins) from the center of the asymmetric septum for sporulating cells (*N* = 129) immobilized in agar pads (see Methods S1). For these measurements, only cells with flat septa (undergoing DNA translocation) were selected. Dotted blue line represents a Gaussian distribution fitted to the experimental data (maximum = 30±5 nm s.e.m.). Black dotted line indicates the septum position. As for cells immobilized in poly-L-lysine, SpoIIIE assembles asymmetrically on the mother cell side of the sporulation septum. More information can be found in Data S1.
(EPS)

Figure S12 Influence of the bacterial tilt angle on the position of SpoIIIE clusters. We performed simulations to test the effect of tilt of the cell axis with respect to the optical axis (φ) in the localization of single-emitters with respect to the septal membrane. From these simulations, we can conclude that our measurements of distances of SpoIIIE clusters to the centre of septa were not affected by tilt. Simulated epifluorescence images of the cell membrane are calculated using an algorithm written in Matlab (Mathworks, version 2011a). We assume that the dye staining the membrane is homogeneously distributed along the bacterium membrane, cell wall, and sporulation septum. The width of the bacterium was considered to be 800 nm and its length 2 µm. The microscope point spread function was assumed to be Gaussian, with a lateral standard deviation σ_x-y_ of 250 nm and an axial standard deviation σ_z_ of 500 nm. The cluster of SpoIIIE proteins was simulated as an ensemble of 600 fluorescent events following a Gaussian distribution with a standard deviation σ of 30 nm. Then, we used our distance determination algorithm (Methods S1) to recover the distribution of distances of single-emitters to the center of the septum. (a) For the first simulation, the cluster of SpoIIIE proteins is placed at the centre of the sporulation septum (yz plane) and on the image plane (xy). (Middle Panel) Simulated image of the cell (white representing membrane stain) and the cluster of SpoIII localizations (green). (Right Panel) As expected, after applying our distance measurement algorithm, the distribution of SpoIIIE localizations (green curve) is perfectly centered with the membrane fluorescence intensity distribution across the septum (orange curve). d indicates the distance between the center of SpoIIIE cluster and center of membrane. (b) If a tilt of 10° is added without changing the position of the cluster or the image plane, almost no change is observed on the simulated image of the cell, though the sporulation septum appears slightly thicker. For tilts φ<10°, the distance d between the SpoIIIE and membrane intensity distributions remains equal to zero, and therefore does not affect our measurements of distance of the cluster to the center of the septum. For values above 15°, the simulated images start to show membrane distribution artifacts that are clearly related to a large tilt and are not observed experimentally. This simulated result suggests that the tilt angle on the sample surface remains below 15° in our experiments. Consistent with this observation, 3D-SIM imaging showed that our protocol for fixing cells in our micro-fluidics chambers produces samples with cells lying flat on the surface with a tilt smaller than 5°. Thus, in our observation conditions, our measurements are not affected by our experimental tilt. (c) To explore the influence of the septal membrane (yz plane) positioning of the SpoIIIE cluster, we simulated a cell tilted by 10° and a SpoIIIE complex outside the image plane. In this case, the cluster will appear shifted from the sporulation septum on the simulated epi-fluorescence image (middle panel), even though the proteins are actually assembled on the septum. Under these conditions, a tilt of 10° and a cluster 50 nm away from the septum centre can lead to a shift as high as ±8 nm along the x direction, depending on the position of the complex along the z-axis (right panel). The detection of single-molecules and the stringent fit of the PSF to a gaussian distribution creates a depth-of-field in PALM imaging that is smaller than a few tens of nanometers; thus, a shift of 8 nm would be an upper bound (i.e., maximum expected systematic error). In order to ensure that this potential systematic error was not influencing our measurements, we investigated the effect of tilt and septal plane positioning on the average cluster-to-septum distance after a series of 150 independent simulations (each representing a single cell with a specific φ angle and a cluster at a specific yz position) in which (1) the radial and angular positions of the cluster in the septal plane (yz) are homogeneously distributed, and (2) the tilt angle follows a normal law centered around zero with a standard deviation σ_φ_ of 10°. (d) As expected from the simulation conditions, the distribution of cluster positions on the septal plane (plotted as a distance distribution in the y-axis) is centered at zero and shows a large dispersion. (e) Importantly, despite this large dispersion of cluster positions on the septal plane, the distances of detected SpoIIIE clusters to the centre of the sporulation septum shows a Gaussian distribution with a mean of zero and a standard deviation of σ = 35 nm. Thus, provided that a large enough sample is used, our method recovers the mean distance of SpoIIIE clusters to the septum in spite of changes in the cell axis with respect to the surface (for angles φ<∼10°) and heterogeneous distributions of SpoIIIE clusters on the septal plane. (f) Finally, we simulated a distribution of clusters located at a distance x_0_ = 30 nm away from the septum in which we randomly varied the position of clusters in the yz plane and the angle φ as in (d–e). Importantly, the distribution of cluster-to-septum distances gives a mean distance <d> = 32±20 nm (s.d.), thus recovering the simulated results despite large dispersions in the yz distributions and φ angles. From these simulations, we conclude that the presence of a Gaussian distribution of tilt angles around φ = 0° (flat) in a population of cells changes the standard deviation of localization measurements but, importantly, not the mean value of the distribution. The dispersion in localization measurements increase with tilt, highlighting the need for an experimental method in which cells are kept as flat as possible and a large number of measurements are made in order to obtain an accurate value for the localization of SpoIIIE clusters. Thus, for experiments performed in micro-fluidics chambers, the influence of tilt in measurements is minimal (as cells were flat within a few degrees and the surface was highly stable). In SpoIIIE localization measurements performed in agar pads (Figure S11d), the dispersion was higher (σ∼60 nm) than that observed in micro-fluidics chambers (σ∼30 nm), likely due to the surface of the pads being not perfectly flat, leading to larger variations in cell orientations. More information can be found in Data S1.
(EPS)

Methods S1 (SM1) PALM microscope setup. (SM2) Coverslip cleaning protocol. (SM3) Cell culture, strains and induction of sporulation. (SM4) Fluidic chamber assembly. (SM5) In vivo PALM imaging with fluidic chamber. (SM6) In vivo PALM experiments on agar pads. (SM7) Alignment correction in PALM experiments. (SM8) Drift correction for PALM experiments. (SM9) Contour calculation and automatic cell sorting. (SM10) SpoIIIE pointillist reconstruction and cluster detection for PALM experiments. (SM11) Calculation of distance between SpoIIIE proteins and the membrane. (SM12) 3D structured illumination microscopy (3D-SIM). (SM13) Two-photon number and brightness analysis. (SM14) Calculation of cluster localization probability.
(PDF)

Movie S1 Two-color 3D-SIM image of a single *B. subtilis* cell in the early stages of formation of the sporulation septum. SpoIIIE clusters (white) localize to the sporulation septum before septal constriction (red).
(MP4)

Movie S2 Two-color 3D-SIM image of a single dividing *B. subtilis* cell during septal invagination. SpoIIIE (white) clusters localize to the leading edge of the closing septum (red).
(MP4)

Movie S3 Two-color 3D-SIM image of a single sporulating *B. subtilis* cell with a mature septum. A single cluster of SpoIIIE (white) localizes to the center of the septal plane (red).
(MP4)

Table S1 Equipment parts.
(DOC)

Table S2 Products and consumables used for the PALM experiments.
(DOC)

Table S3 Bacterial strains used in this work.
(DOC)

## Abbreviations

3D-SIM: 3 dimensional structured illumination microscopy
CDR: chromosome dimer resolution
dsDNA: double-stranded DNA
FWHM: full width at half maximum
KOPS: FtsK Orienting Polar Sequences
LB: Luria-Bertani medium
N&B: Number and Brightness analysis
PALM: photo-activated localization microscopy
SpoIIIE_αβγ_/FtsK_αβγ_: SpoIIIE or FtsK αβγ domains
SpoIIIE_L_/FtsK_L_: SpoIIIE or FtsK linker domains
SpoIIIE_N_/FtsK_N_: SpoIIIE or FtsK N-terminal membrane-anchoring domains
SRS: SpoIIIE Recognition Sequence
