# Supplementary material for: Recruitment, Assembly, and Molecular Architecture of the SpoIIIE DNA Pump Revealed by Superresolution Microscopy
Source: PLoS Biol. 2013 May 7;11(5):e1001557. doi: 10.1371/journal.pbio.1001557 (PMC3646729; doi:10.1371/journal.pbio.1001557)
Supplement: Data S1 — Document containing all supplementary figures, captions, and additional comments regarding supplementary figures. (PDF) [file pbio.1001557.s001.pdf]

Recruitment, assembly and molecular architecture of the SpoIIIE DNA pump revealed by super-resolution microscopy

Fiche[1], JB., Cattoni[1], DI., Diekmann[1], N., Langerak[2], J., Clerte[1], C., Royer[1], C.A., Margeat[1], E., Doan[3],T., Nöllmann[1#], M.

Supplementary Data (SD)

SD 1. Time-trace analysis of single PALM-limited and dynamic clusters 2

SD 2. Mobile nature of dynamic clusters 4

SD 3. Probability distributions of number of single-molecule events detected in PALM-limited and dynamic clusters for sporulating and exponentially growing cells 6

SD 4. SpoIIIE-mMaple assembles in PALM-limited clusters in all cell-cycle stages, and localizes specifically to symmetric and asymmetric septa. 9

SD 6. Distributions of PALM-limited, dynamic and mixed clusters in sporulating and exponentially growing cells 13

SD 7. Heat maps representing the spatial probability distributions of PALM-limited and dynamic SpoIIIE clusters in vegetative/pre-divisional, dividing and sporulating cells 14

SD 8. 3D-SIM imaging of SpoIIIE during early and late stages of septum formation in cells undergoing symmetric division 16

SD 9. Number of clusters detected in sporulating and exponentially growing cells by 3D-SIM 18

SD 10. SpoIIIE localizes to FtsZ rings in dividing cells 19

SD 12. Influence of the bacterial tilt angle on the position of SpoIIIE clusters 23

Bibliography 26

SD 1. Time-trace analysis of single PALM-limited and dynamic clusters

Supplementary Figure 1.

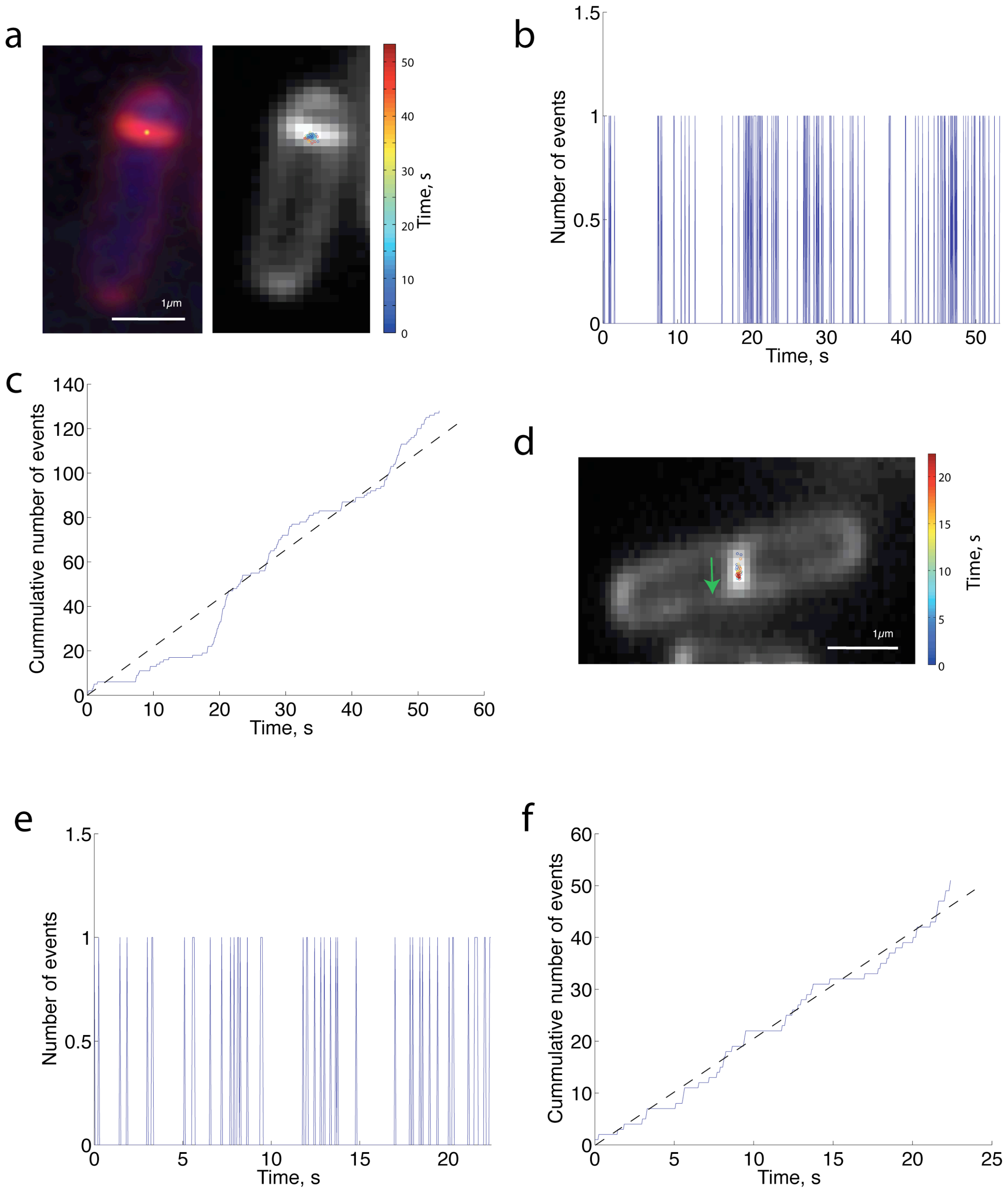

(a) A PALM reconstruction and a pointillist representation of a PALM-limited cluster in which single localizations are color-coded by time (complete time-series for that cluster). The position of early (blue) or late (red) localization events does not vary over time, indicating that the cluster is stationary during the acquisition time.

(b) Number of events detected in the region of interest (ROI, in this case the whole cell) containing the PALM-limited cluster shown in (a) as a function of time. Single, non-

overlapping localization events are detected and dark times between events are longer than average emission times. This data is consistent with each photo-activatable protein being imaged until photo-bleached with no noticeable overlapping between events. In this case, the total number of emission bursts was 96, giving an estimate of ~28 fluorescent proteins [1].

(c) Cumulative number of events detected as a function of time for the ROI in (a). From this data, it is clear that photo-activation rates are in average homogeneous during acquisition (dashed line is a guide to the eye).

(d) A pointillist representation of a dynamic cluster in which single localizations are color-coded by time (complete time-series for that cluster). Here, localization events spread over several pixels and follow a path from the top to the bottom of the septum (see green arrow).

(e) Number of events as a function of time detected in the cell represented in panel (d). Single, non-overlapping localization events are detected and dark times between events are longer than average emission times. In this case, the total number of emission bursts was 37, giving an estimate of ~10 fluorescent proteins [1].

(f) Cumulative number of events detected as a function of time for the ROI in (d). Photo-activation rates are in average homogeneous during acquisition (dashed line is a guide to the eye).

The probability of photo-activation of a single protein in a diffraction-limited spot depends not only on the intensity of the photo-activation laser and the photo-physics of the probe but also on the density of proteins: small clusters with a large number of proteins will have to be photo-activated with lower photo-activation intensity than clusters with fewer proteins. Our PALM imaging conditions were optimized to image the most dense clusters as rapidly as possible, while ensuring that a single protein is photo-activated per diffraction-limited spot at any given time. Thus, in a field-of-view with many clusters with different protein densities being imaged in parallel, the imaging time of the less dense clusters may not be fastest possible for that cluster, but is set to ensure that in all cases a single-protein was detected per single diffraction-limited spot at any given time (an essential requirement of PALM imaging).

SD 2. Mobile nature of dynamic clusters

As described in Fig 2b-c, SpoIIIE distributed in two cluster types. The first, contained thousands of single-molecule events and were small in size (~45 nm FWHM, PALM-limited). The second had large sizes and a smaller number of single-molecule events. In order to determine the nature of the second cluster type, we analyzed the number of single-molecule localizations and their trajectories. To identify whether these clusters represented a dispersed, static population of single-emitters or rather single-molecules moving during the hundreds/thousands of individual acquisitions required for a PALM image, we investigated the trajectory of localizations (Supplementary Fig. 1a, d). Analysis of trajectories in dynamic clusters show that some molecules move dynamically whereas others remain static during the acquisition of a PALM image series (Supplementary Fig. 2c). The former could represent single diffusing emitters whilst the latter display confinement zones, suggesting transient interactions with cytoskeletal membrane-bound factors. From these analyses, we cannot discard that some clusters classified as dynamic may contain a dispersed population of static single emitters, however, the proportion of these occurrences is small and does not affect our conclusions in the main text.

Supplementary Figure 2.

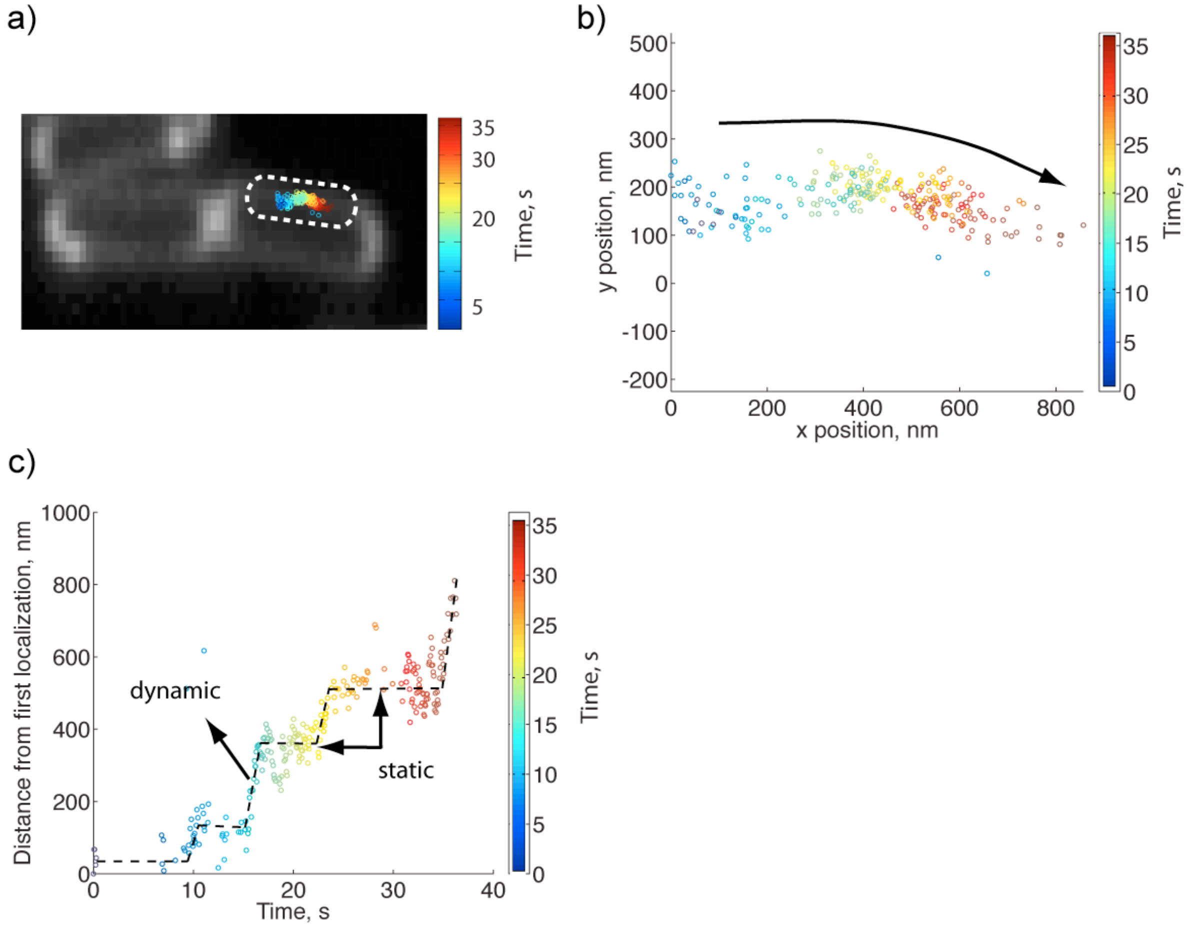

(a) Pointillist reconstruction of single molecule events (green) detected in a *B. subtilis* cell in stage 1. Single events inside the white dotted area were automatically classified as part of a dynamic cluster. **(b)** Representative trajectories generated from tracking the motion of single-localizations identified in panel **a** (color coded by time of detection: red, green and blue). The solid line is a guide to the eye. **(c)** Spatial and temporal evolution of dynamic clusters. Distance between the initial position of the first assigned location of the cluster and subsequent positions as a function of time. Dynamic and static sub-clusters are indicated with an arrow. Dashed line is a guide to the eye.

SD 3. Probability distributions of number of single-molecule events detected in PALM-limited and dynamic clusters for sporulating and exponentially growing cells

Supplementary Figure 3

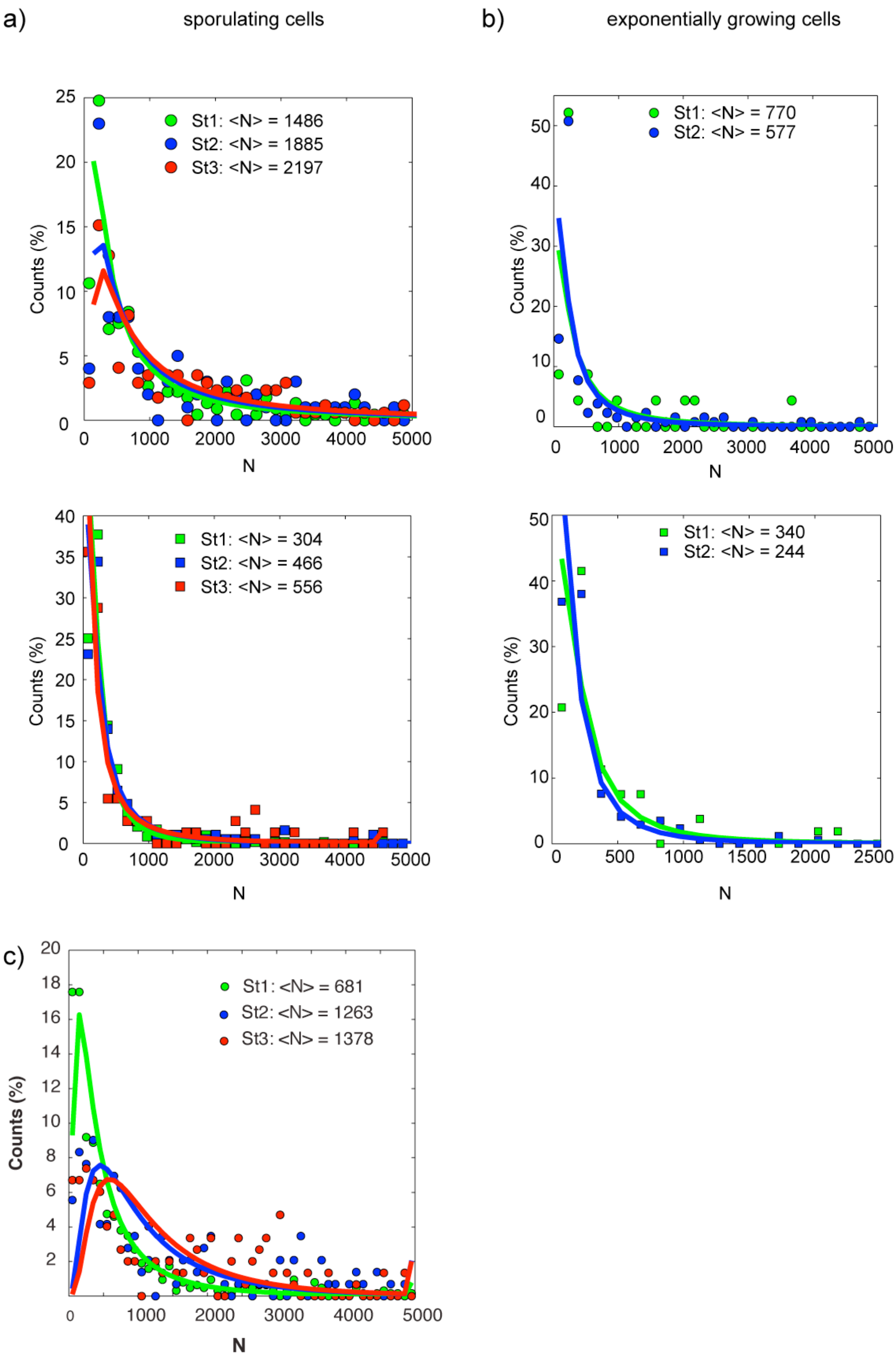

The number of detected single-molecule events per cluster (*N*, or cluster composition) does not provide absolute protein numbers. However, *N* is still proportional to the number of single emitters, and can thus be used to obtain relative measures of the number of proteins in different cluster types and different cell cycle stages.

N was calculated for each cluster type (PALM-limited (top) or dynamic (bottom)) in vegetative/pre-divisional, dividing and sporulating cells. The *N* frequency distribution is plotted on the top histograms for PALM-limited clusters and on the bottom panels for dynamic clusters. In each plot, distributions for vegetative/pre-divisional (green), dividing (blue) and sporulating (red) cells are shown. A log-normal function was fitted to each *N* distribution in order to estimate the average number of events (<*N*>) for each type of clusters. **(a)** *N* distributions in cells after 2h of sporulation induction. Here, *N* depends only weakly on cell stage, although clusters in sporulating cells tend to have higher numbers of molecules. In average, PALM-limited clusters possess ~4 times more SpoIIIE molecules than dynamic clusters. **(b)** *N* distributions in exponentially growing cells. Under these conditions, *N* distributions did not depend strongly on cell cycle stage, however PALM-limited clusters contained only ~2-3 times more proteins than dynamic clusters. Importantly, the overall number of proteins detected was independent on cluster type or cell cycle stage, and was ~2.5-fold higher in sporulating than in exponentially growing cells. **(c)** Number of detected single-molecule events per cell (N_{cell}), in cells after 2h of sporulation induction. N_{cell} depends only weakly on cell stage, although sporulating cells tend to have higher numbers of molecules. There are large cell-to-cell variations in the number of events detected, possibly due to stochastic differences in SpoIIIE expression levels and due to a poorer detection of freely diffusing monomers under our acquisition conditions.

Proteins of the Eos family are known to shown intermittency [2]. To determine whether *N* distributions corresponded to single or multiple emitters, we performed i*n vitro* experiments in which we deposited a diluted sample of recombinant mEos2 on a surface and detected the emission profiles of hundreds of surface-immobilized single-molecules under the same experimental conditions (laser wavelength and power, camera settings, etc) as those used in live PALM experiments (A. Valeri, J.B. Fiche, and M. Nollmann, submitted). We determined that single mEos2 proteins produce an average of ~60 single-molecule events. From these measurements, we can approximately estimate that PALM-limited clusters contain ~36 SpoIIIE proteins, in agreement with our absolute measurements of protein numbers by N&B analysis. In contrast, dynamic clusters contained in average ~400 events, consistent with ~10 single emitters per cluster.

SpoIIIE is under the expression of a constitutive promoter. Our experiments in vegetatively growing cells are performed after 3-4 hours of growth in Luria Bertani (20%) medium, whereas sporulating cells were further incubated for 2hrs to induce sporulation. Thus, the maturation time of eosFP (~1hrs [3]) or GFP (few minutes for mut2 [4]) should not have an influence on the steady-state number of SpoIIIE proteins at the time of observation in vegetatively growing or sporulating cells.

Detection of monomeric SpoIIIE species

Typical membrane proteins in bacteria move with diffusion coefficients ranging from 0.005-0.1 μm²/s [5]. By assuming the highest diffusion coefficient of 0.1 μm²/s for SpoIIIE monomers and taking into account our acquisition time, we calculate that the root mean square displacement of a SpoIIIE monomer can be at most ~150 nm, which is under the diffraction limit in our conditions (218 nm). Thus, in our PALM experiments the emitted fluorescence signal of a single diffusing monomer should in average not blur, and it should thus be detected in our setup. As reported diffusion coefficients reflect the average movement of proteins, we cannot discard that a low frequency of fast diffusing monomers could have been missed under our acquisition conditions due to an asymmetry in their emission point-spread function. A second reason why freely diffusing monomers or small clusters may be detected with a lower efficiency has to do with their spatial localization. The selection of the focal plane for each field of view is optimized to focus at the center of the population of bacteria in the field. We estimate that the focal depth in our experiments is ~ 300-400 nm (smaller than the FWHM of the point-spread function in the axial direction as the process of localization would tend to discard events with a low signal to noise ratio and with a width incompatible with the PSF expected for a single emitter). Thus, detection of proteins in the apical or basal membranes would have a lower efficiency than that of proteins in the focal plane. This effect would tend to have a stronger effect in monomeric diffusing proteins that in clusters. These combined effects could then lead to a small underestimation in the number of events and clusters per cell, an effect that would be more acute in cells in which SpoIIIE is not assembled or interacting with division components.

SD 4. SpoIIIE-mMaple assembles in PALM-limited clusters in all cell-cycle stages, and localizes specifically to symmetric and asymmetric septa.

Supplementary Figure 4

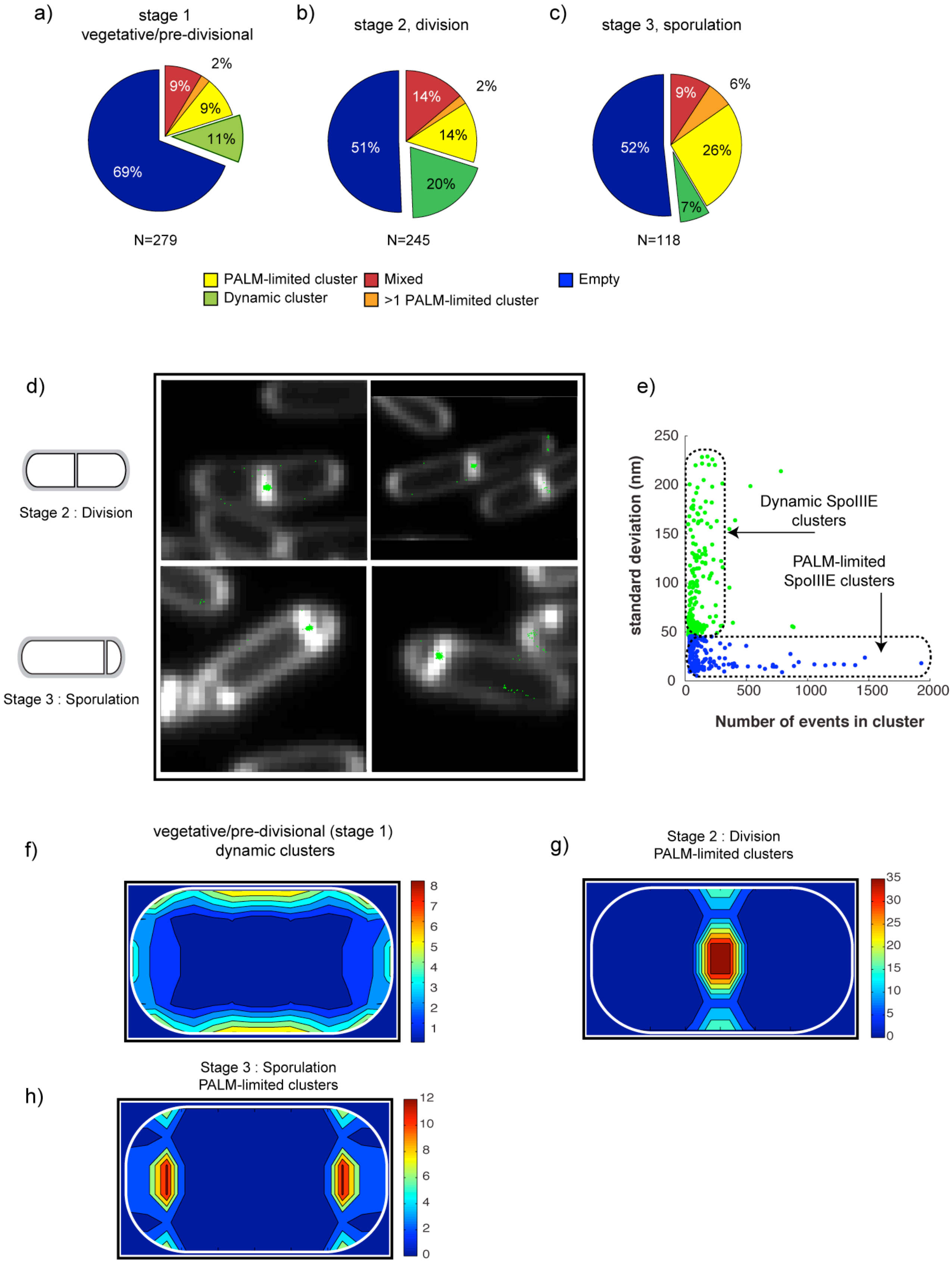

(a-c) Statistics of SpoIIIE clusters in vegetative/pre-divisional (N=279), dividing (N=245) and sporulating (N=118) cells using a SpoIIIE fusion to the photo-activatable protein mMaple [6]. Clusters were automatically classified as dynamic, PALM-limited or mixed (cells containing both cluster types). Cells with less than 10 events were classified as 'empty', and cells in which all clusters had less than 25 events were also classified as 'empty'. Cells with more than one PALM-limited cluster were classified independently from those containing a single one. SpoIIIE-mMaple PALM-limited clusters are present in considerable proportions in all cell-cycle stages. The proportions of empty cells are larger than those shown in Fig. 3a-c due to the lower number of events detected for the mMaple fusion (cells with <10 events are considered empty and clusters with <25 events are discarded). The proportion of single PALM-limited clusters increases from vegetative/pre-divisional to dividing cells, and is maximal in sporulating cells (9% in vegetative/pre-divisional, 14% in dividing, and 26% in sporulating cells), a trend that is also observed for SpoIIIE-eosFP (17% in vegetative/pre-divisional, 25% in dividing, and 52% in sporulating cells). Importantly, the proportions between cells containing PALM-limited clusters versus those containing only dynamic clusters are maintained: (i) 62% for mMaple/ 45% for eosFP in vegetative/ pre-divisional cells; (ii) 60% for mMaple/ 62% for eosFP in dividing cells; (iii) 83% for mMaple/ 89% for eosFP in sporulating cells.

(d) Pointillist representation of SpoIIIE-mMaple localization in dividing (top panels) and sporulating (bottom panel) cells show that SpoIIIE-mMaple PALM-limited clusters localize to the center of symmetric and asymmetric division septa.

(e) Analysis of the cluster size distribution versus the number of mMaple single-molecule events shows two distinct cluster types: PALM-limited clusters (red dots) have a size equal or smaller (~45 nm FWHM) than the resolution of PALM in our conditions and contain a large number of events (> 300), whilst dynamic clusters (orange dots) are large (σ>45 nm equivalent to >100 nm FWHM) and contain fewer events (< 300). Note that the ordinate shows the standard deviation of the cluster size, rather than the FWHM (with FWHM ~ 2.2 σ). The difference between the mean number of events detected in dynamic and PALM-limited clusters in SpoIIIE-eos or SpoIIIE-mMaple reflect the different photo-physical behavior of these proteins. In both cases, there is a clear distinction between the number of events and the size of PALM-limited and dynamic clusters.

(f-h) The normalized coordinates of each localized event (axial and longitudinal coordinates) were used to calculate the localization probability distribution (heat maps) of SpoIIIE for each cluster type in vegetative/pre-divisional, dividing, and sporulating cells. Normalized localization probability distributions were calculated for the first quartile of the cell and then reflected into the other three quartiles to impose mirror symmetry in the axis perpendicular and parallel to the cell axis. The relative average number of clusters detected in each pixel of the grid is color-coded according to the color bar (right). **(f)** SpoIIIE-mMaple dynamic clusters distribute homogeneously over the cell membrane. (**g-h**) In contrast, heat maps of PALM-limited clusters in (g) dividing (N=245) and (d) sporulating (N=118) cells show a clear SpoIIIE-mMaple localization to the center of the septal plane.

SD 5. Number and Brightness analysis of exponential and sporulating cells undergoing cell division or sporulation

Supplementary Figure 5

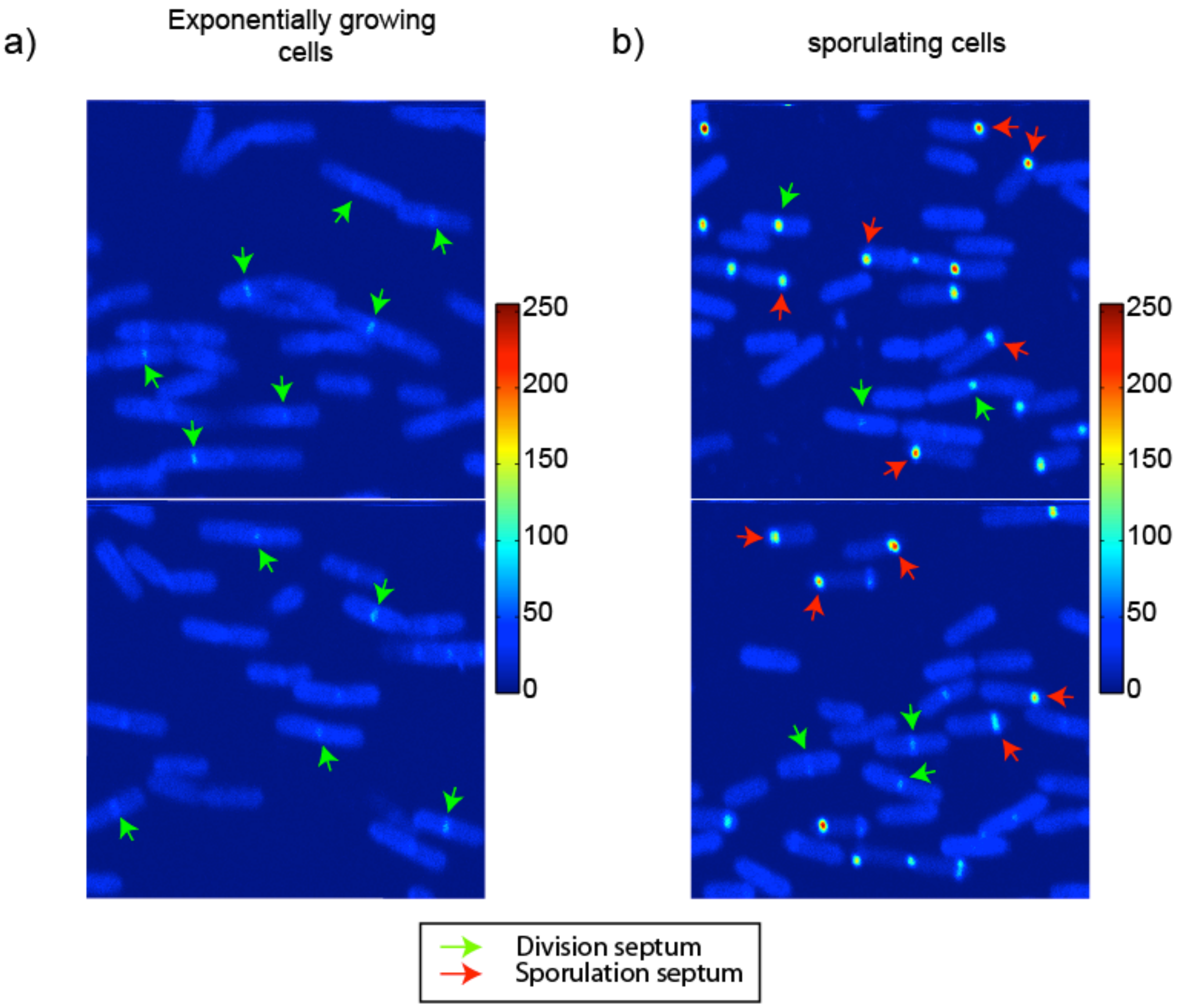

Number and Brightness analysis in exponentially growing or sporulating cells. Cells growing exponentially **(a)** or after induction of sporulation **(b)** were imaged by two-photon laser scanning microscopy (see Supplementary Methods). The intensity fluctuations at each pixel in a series of rapid raster scanned images of bacteria are used to deconvolve the average intensity (counts/s) into the molecular brightness (counts/s/molecule) and absolute number of fluorescent proteins inside individual bacterial cells [7,8]. Colour-coded scale indicates the intensity level detected per excitation volume (0.07 fL inside a typical *B. subtilis* cell). Green and red arrows indicate the estimated position of division and sporulation septa, respectively. Brightness of monomeric GFP was obtained in a strain in which monomeric GFP was expressed in the cytosol [7] (Supplementary Methods).

Sample	Number of bacteria analyzed	Brightness	Normalized number of proteins
Monomeric GFP	60	0.15 ± 0.02	-
SpoIIIE clusters - division (stage 2)	100	0.26 ± 0.05	18 ± 4
SpoIIIE clusters - sporulation (stage 3)	125	0.51 ± 0.15	47 ± 20

From these data, an average number of SpoIIIE proteins at the septum (either division or sporulation) was estimated. For dividing cells in exponentially growing conditions, we found 18 ± 4 SpoIIIE proteins at the division septum. In contrast, for sporulating cells, the number of SpoIIIE proteins at division/sporulation septa was equal to 47 ± 20, corresponding to a 2.6 fold increase. Interestingly, this behavior is in very good agreement with the results obtained by PALM for the analysis of SpoIIIE cluster composition (see Supplementary Fig. 3), in which a 2-3 fold increase in the number of fluorescent events was observed between sporulating and exponentially growing cells.

The Brightness data can also be used to determine the oligomerization state of SpoIIIE proteins. In sporulating conditions, the brightness measured for SpoIIIE foci is in average 3.4 times higher than for monomeric GFP. Assuming SpoIIIE proteins are either found in a monomeric state or in hexamers, our brightness measurements suggest that in PALM-limited clusters only 30% of the proteins exist as monomers, while the remaining 70% assemble into hexamers. On the other hand, for exponentially growing cells, the brightness is only 1.7-fold higher as compared to monomeric GFP, suggesting that 58% of the proteins are in a monomeric state. Due to the possibility that a fraction of SpoIIIE proteins remain immobile during the acquisition of the N&B dataset (which requires ~4 min), our estimates of the proportion of hexamerized proteins are a lower bound (i.e. at least 70% or 42% of proteins are in hexameric form during sporulation or division, respectively).

SD 6. Distributions of PALM-limited, dynamic and mixed clusters in sporulating and exponentially growing cells

Supplementary Figure 6

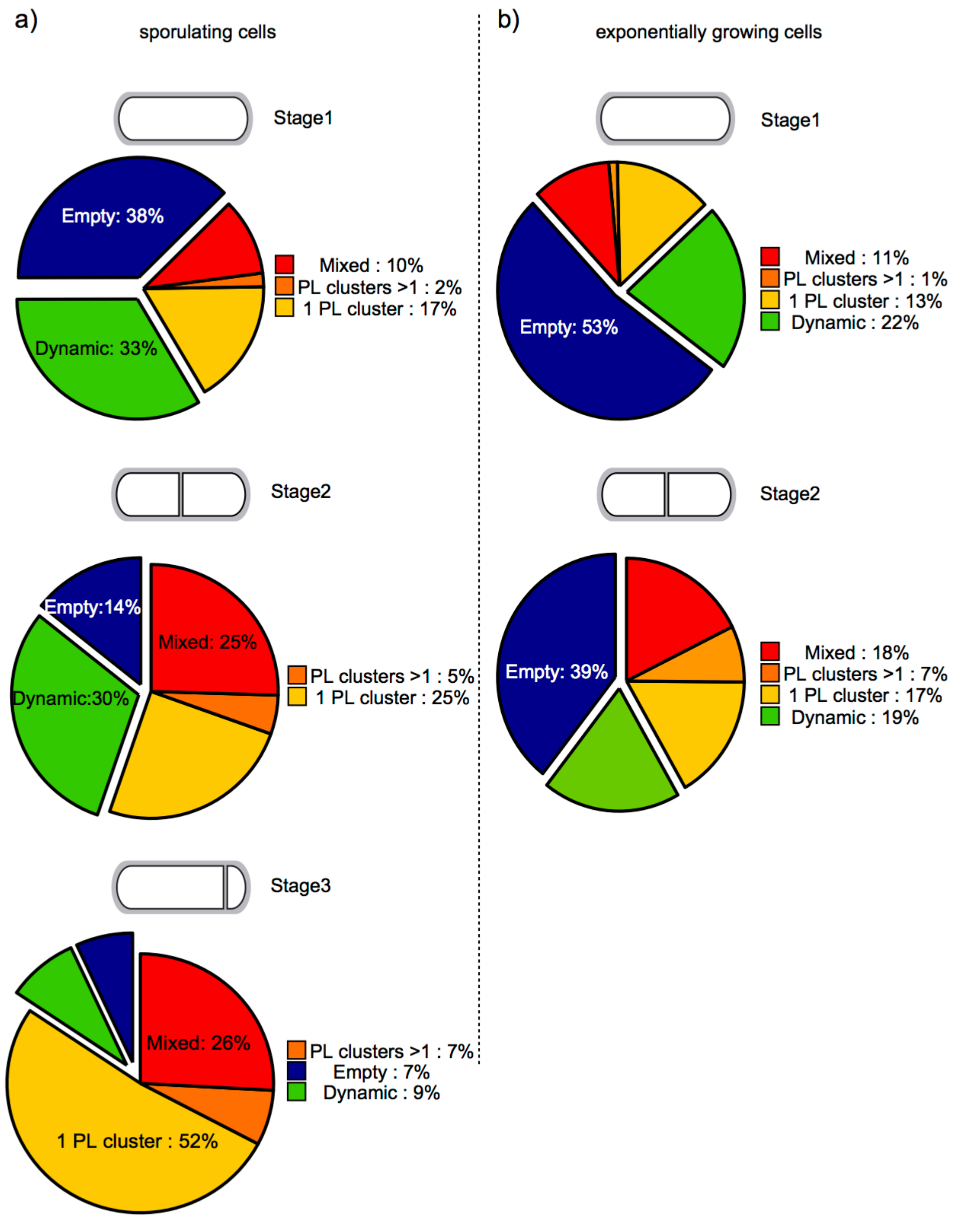

(a) Sporulating (same as in Fig. 3a-c) and **(b)** exponentially growing cells were imaged by PALM, and cells were classified as vegetative/pre-divisional, dividing or sporulating (stages 1, 2 and 3, respectively). In each stage, SpoIIIE clusters distribution was statistically analysed. The proportion of cells with no clusters detected (empty) in stage 1 and 2 was significantly higher for cells growing exponentially than in sporulating cells. However, the relative proportions of PALM-limited and dynamic clusters remained unchanged. In stage 3 cells, the total number of both types of PALM-limited clusters increased significantly with respect to cells in either stage 1 or 2.

SD 7. Heat maps representing the spatial probability distributions of PALM-limited and dynamic SpoIIIE clusters in vegetative/pre-divisional, dividing and sporulating cells

Supplementary Figure 7

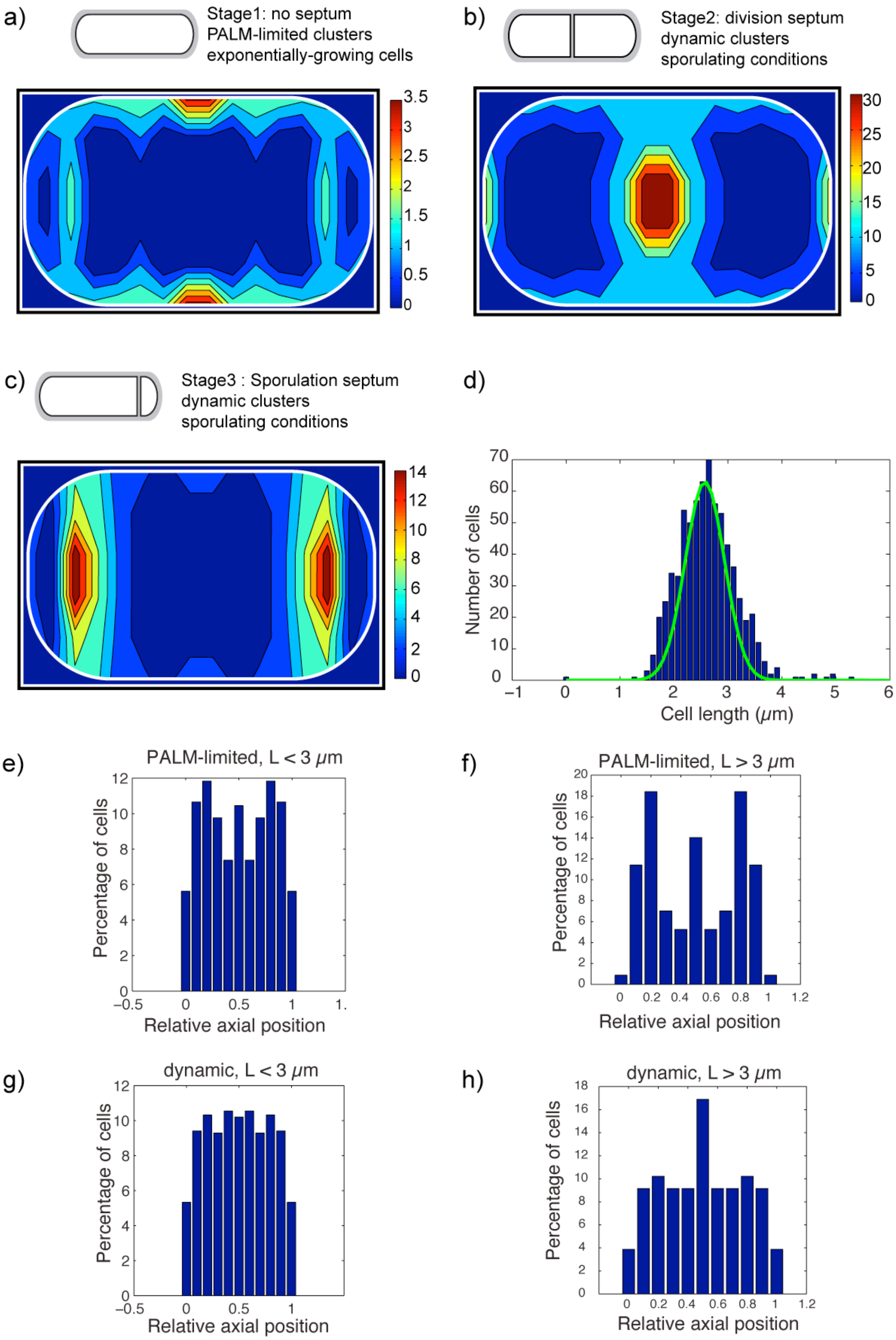

Heat maps representing the localization statistics of individual PALM-limited and dynamic clusters in pre-divisional, division and sporulating cells were built as described in Fig. 3d and Supplementary Methods. White lines represent cells outlines and the relative density of clusters detected in each position is color-coded according to the colour bar (right side of each figure).

(a) PALM-limited clusters in exponentially growing cells (stage 1) are predominantly found at positions where a division septum would be expected during symmetric cell division. A very small proportion of PALM-limited clusters is also detected at future asymmetric sites. This observation is not unexpected since exponentially growing cells were incubated in sporulation medium for 20 min to reduce cytoplasmatic background fluorescence.

(b-c) Dynamic clusters in a sporulating culture, for cells with a symmetric division septum (b) or an asymmetric sporulation septum (c) are mostly localized to the center of symmetric and asymmetric septa. Distributions are slightly larger (in the septal plane) than those observed for PALM-limited clusters (Fig. 4c-d). In contrast to the strong localization of PALM-limited clusters to the center of the division and sporulation septa (Fig. 4c-d), a small proportion of dynamic clusters can be detected along the cell wall and at the poles (left).

(**d**) Distribution of cell lengths from exponentially growing cultures (blue bars), and Gaussian fit (green solid line) indicating an average size of 2.5 ± 0.5 μm (s.d.).

(**e-f**) Distribution of localization of PALM-limited clusters in cells with lengths smaller (e) or larger (f) than 3 μm. PALM-limited clusters localize to future sites of symmetric or asymmetric septation in longer cells (panel f, pre-divisional cells), but show a homogeneous localization in small cells (panel e, newly born or still vegetatively growing). These results are consistent with SpoIIIE preferably localizing to new division sites in pre-divisional but not in vegetatively growing cells.

(**g-h**) Distribution of localization of dynamic clusters in cells with lengths (L) smaller (g) or larger (h) than 3 μm. As PALM-limited, dynamic clusters localize to future sites of symmetric septation in long cells (panel h, pre-divisional cells), but show a rather homogeneous localization pattern in small cells (panel g, newly born or still vegetatively growing).

SD 8. 3D-SIM imaging of SpoIIIE during early and late stages of septum formation in cells undergoing symmetric division

Supplementary Figure 8

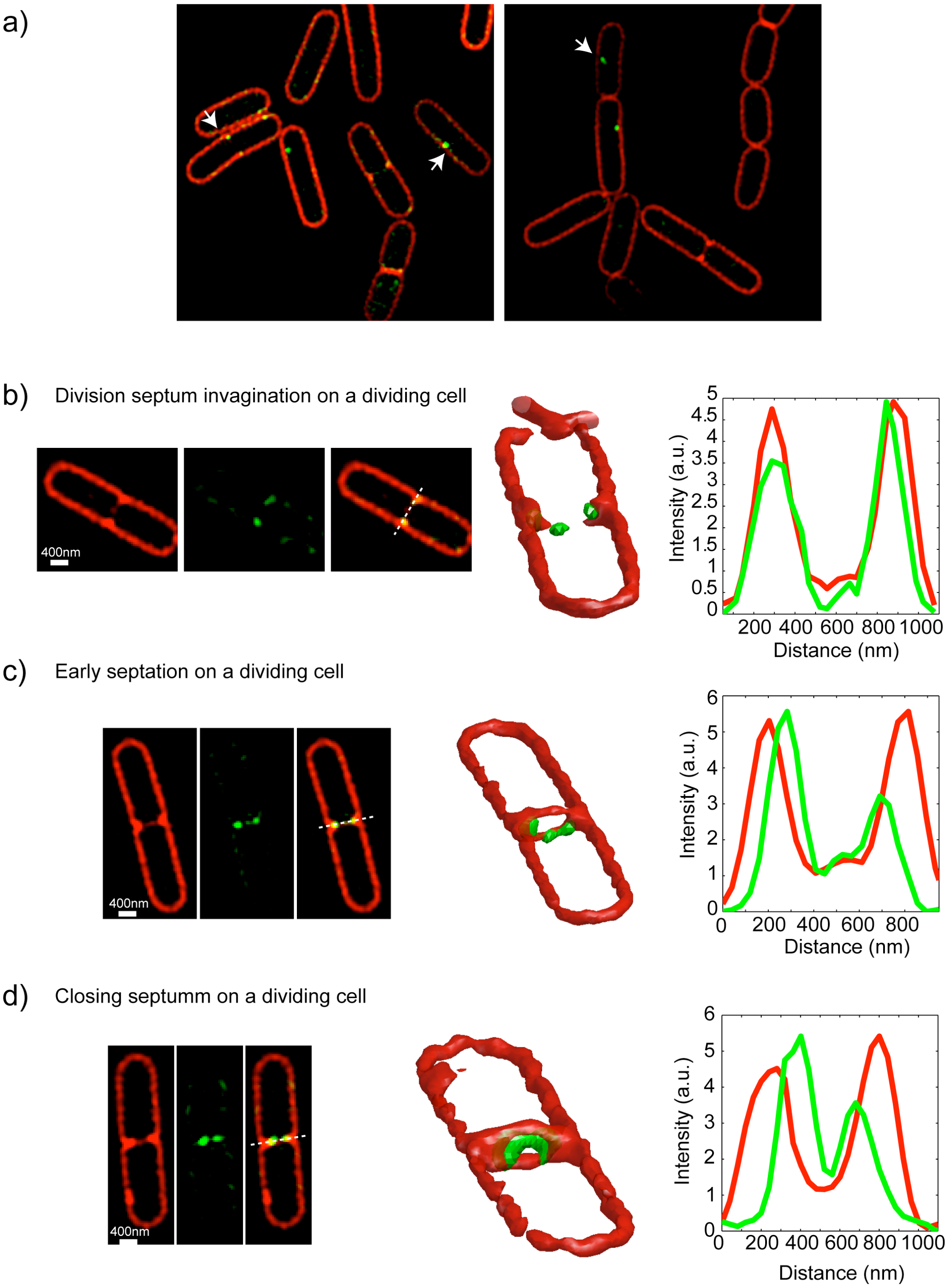

(a) Localization of SpoIIIE in vegetative/pre-divisional (stage 1) cells from an exponentially-growing culture. Single clusters of SpoIIIE localize to future sites of symmetric division (white arrow heads) before a septum can be detected. As shown in these 3D-SIM images, cells lye flat on the micro-fluidics chamber.

(b-d) Localization of SpoIIIE-GFP at different stages of septum invagination during symmetric cell division in *B. subtilis* by 3D-SIM. Three stages of advancement of the septum formation are displayed: **(b)** nascent, **(c)** early septation, and **(d)** and closing septum. For each stage, the left panel represents a z-stack where the first image represents membrane (stained with FM4-64, red), the middle image shows the localization of SpoIIIE (green), and the last image displays the overlay of fluorescence from SpoIIIE and membrane. A 3D reconstruction displaying membrane (red) and SpoIIIE (green) fluorescence signals is shown in the middle panel. In these different stages, SpoIIIE distributes either in single clusters (b-c) or along an arc (d). The latter probably due to the intrinsic dynamical behavior of invaginating septa. Finally, the right panel shows the quantification of fluorescence intensity from the membrane (red solid line) and SpoIIIE (green solid line) along the septum. Combined to the 3D reconstructions, these curves clearly show that SpoIIIE follow the leading edge of the closing septum. Interestingly, at the onset of invagination SpoIIIE localizes with the septal membrane, but shifts to the leading edge as invagination progresses. The white dotted line in the right image of the left panel indicates the direction used to calculate intensity profiles shown in the panels on the right.

Scale bar, 400 nm.

SD 9. Number of clusters detected in sporulating and exponentially growing cells by 3D-SIM

Supplementary Figure 9

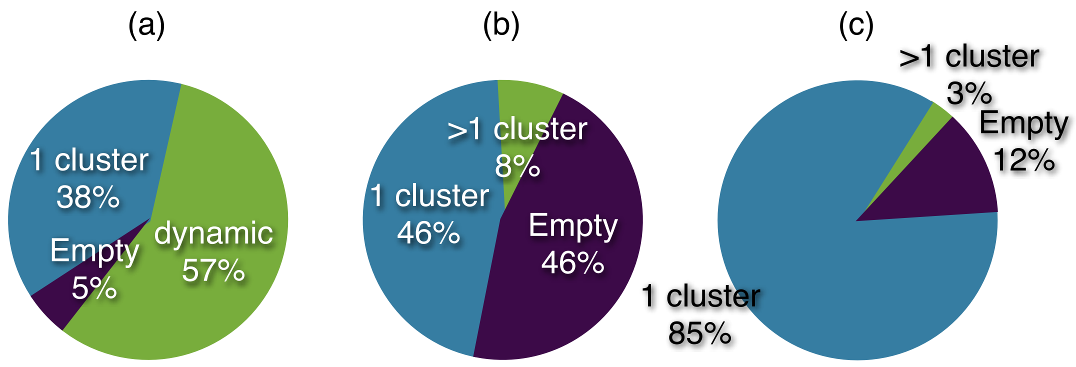

(a) 3D-SIM statistics for sporulating cells with incomplete asymmetric septa (undergoing constriction). SpoIIIE appears in both single and dynamic clusters (see Supplementary Fig. 8b-d). In dynamic clusters, SpoIIIE localizes mainly in arcs accompanying the leading edge of the invaginating septum (see Fig. 3g iv, and Supplementary Fig. 8d), consistent with the intrinsic dynamics of the septal membrane during constriction. **(b)** 3D-SIM statistics for exponentially growing cells undergoing division and displaying a mature (symmetric) division septum (stage 2). Here, half the cells show a single cluster of SpoIIIE at the division septum. **(c)** 3D-SIM statistics for sporulating cells displaying a mature sporulating septum (stage 3). Here, SpoIIIE predominantly assembles in single clusters with a size smaller than the lateral resolution limit of 3D-SIM (<100 nm).

SD 10. SpoIIIE localizes to FtsZ rings in dividing cells

Supplementary Figure 10

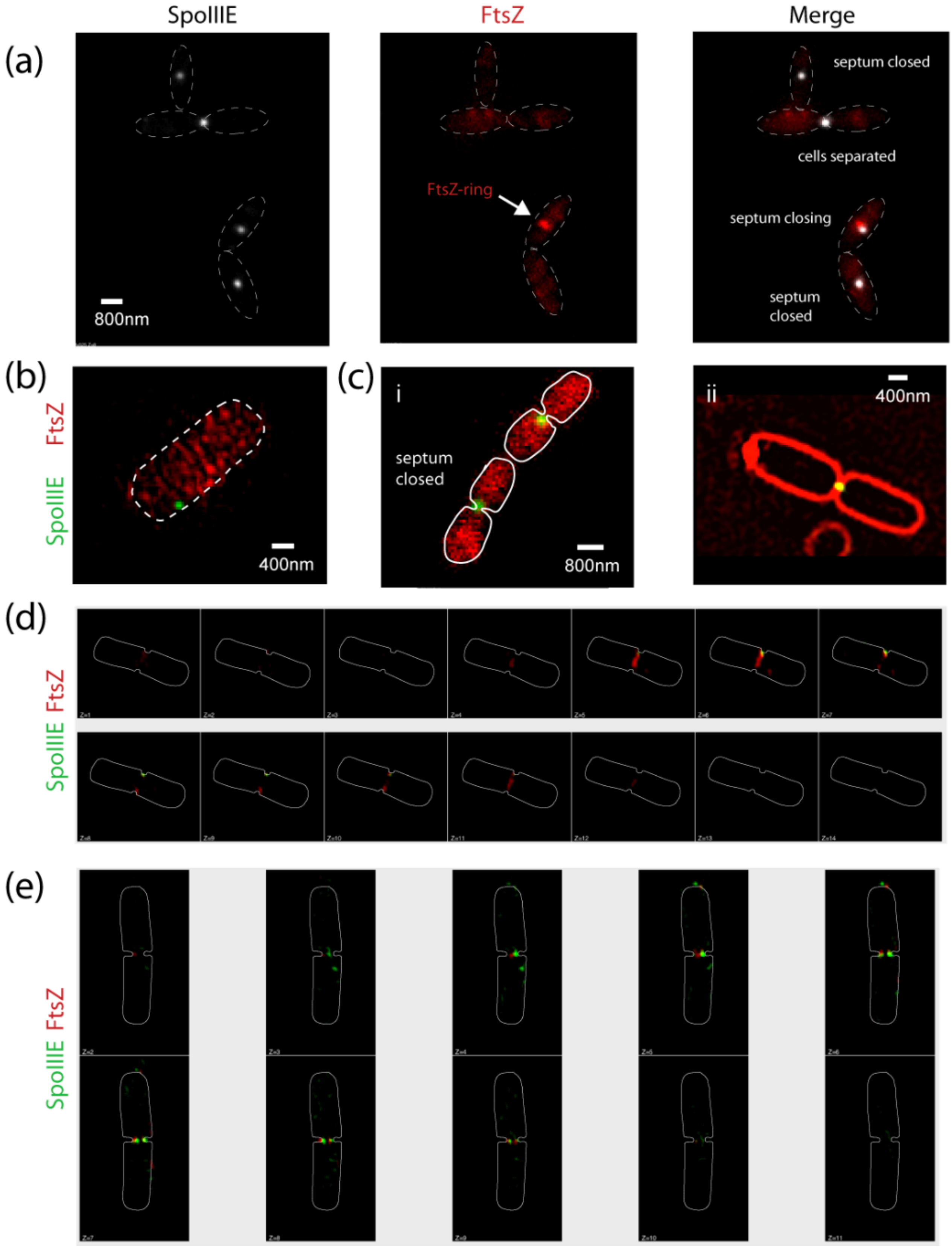

(a) Wide-field epi-fluorescence imaging of SpoIIIE (white) and FtsZ (red). A field of view display cells in different cell cycle stages, with SpoIIIE assembling in clusters during (i)

closure of the FtsZ septal ring, (ii) at the end of septation (FtsZ fluorescence becomes cytoplasmic), and (iii) after cell separation. 3D-SIM images were selected whenever possible but the same results were obtained with wide-field epi-fluorescence imaging.

(b) 3D-SIM imaging of SpoIIIE (green) and FtsZ (red). SpoIIIE looses its localization pattern in vegetative cells (i.e. small cells with no visible FtsZ ring) in 75% of cells (N=104).

(c) (i) Wide-field epi-fluorescence imaging of SpoIIIE (green) and FtsZ (red). SpoIIIE often assembles in a cluster at the end of septal constriction when FtsZ becomes cytosolic (30%, N=104). Note that in the terminology employed here, a 'closed septum' is a septum that has completed invagination but not necessarily fused membranes. (ii) 3D-SIM imaging of SpoIIIE (green) and membrane stain (red). SpoIIIE often remains localized to the septum after cell separation (30%, N=77). 3D-SIM images were selected whenever possible but the same results were obtained with wide-field epi-fluorescence imaging.

(d) Montage of a 3D-SIM image of SpoIIIE (green) and FtsZ (red), with z representing different z-stacks separated by 130 nm. SpoIIIE localizes to the FtsZ-ring in early dividing cells (ring diameter ~700 nm). 3D reconstruction and fluorescence line-scan are shown in Fig. 4a.

(e) Montage of a 3D-SIM image of SpoIIIE (green) and FtsZ (red), with z representing different z-stacks separated by 130 nm. SpoIIIE localizes to the FtsZ-ring in a cell that almost completed invagination (ring diameter ~300 nm). 3D reconstruction and fluorescence line-scan are shown in Fig. 4b.

SD 11. Effects of cell orientation in the localization of SpoIIIE, conservative classification of PALM-limited cluster distributions, and single-molecule SpoIIIE localizations with respect to the center of sporulation septa in agar pads.

Supplementary Figure 11

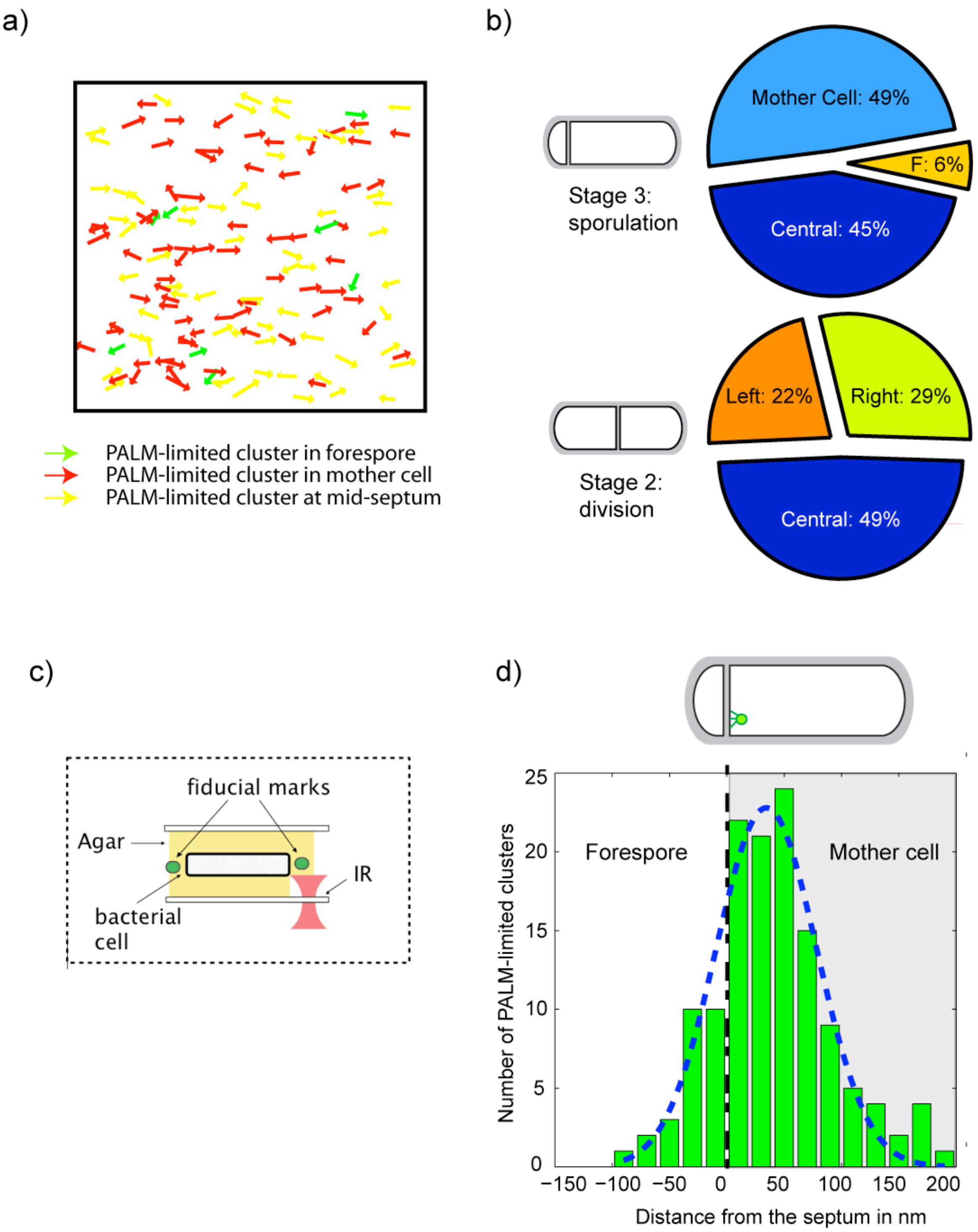

(a) Schematic representation of the directions of long axes of sporulating bacteria accumulated in a single field of view from different PALM imaging experiments. For each single cell, an arrow is represented, its position representing the location of the cell in the

field of view, its direction representing the long axis of the cell, and its orientation pointing towards the direction of the sporulation septum. The colour of each arrow indicates the calculated position of a SpoIIIE cluster with respect to a sporulating septum (green for forespore, red for mother cell, and yellow undefined). The direction and location of arrows is uncorrelated with the septal localization of SpoIIIE clusters, indicating that the relative position of SpoIIIE clusters with respect to sporulation septa is independent of cell orientation or position in the field of view.

(b) The position of PALM-limited clusters with respect to the center of dividing or sporulating septa was calculated as described in Supplementary Methods. From these measurements, clusters were conservatively classified as in the mother cell compartment (distance >15 nm), central (-15>distance<15 nm), or in the forespore compartment (distance <-15 nm) (N=43). For cells undergoing symmetric division, clusters were similarly classified as 'central', or on the 'left', or 'right' compartments (N=71).

(c) Experimental setup used to immobilize cells (black cylinder) in agar pads (yellow). Fiducial marks (green spheres) were used to correct for lateral drift during acquisition and an IR laser to correct for axial drift in real-time. More details are provided in the Supplementary Methods section.

(d) Distribution of distances of single emitters (proportional to the number of single SpoIIIE proteins) from the center of the asymmetric septum for sporulating cells (N=129) immobilized in agar pads (see Supplementary Methods). For these measurements, only cells with flat septa (undergoing DNA translocation) were selected. Dotted blue line represents a Gaussian distribution fitted to the experimental data (maximum = 30 ± 5 nm s.e.m.). Black dotted line indicates the septum position. As for cells immobilized in poly-L-lysine, SpoIIIE assembles asymmetrically on the mother cell side of the sporulation septum.

SD 12. Influence of the bacterial tilt angle on the position of SpoIIIE clusters

Supplementary Figure 12

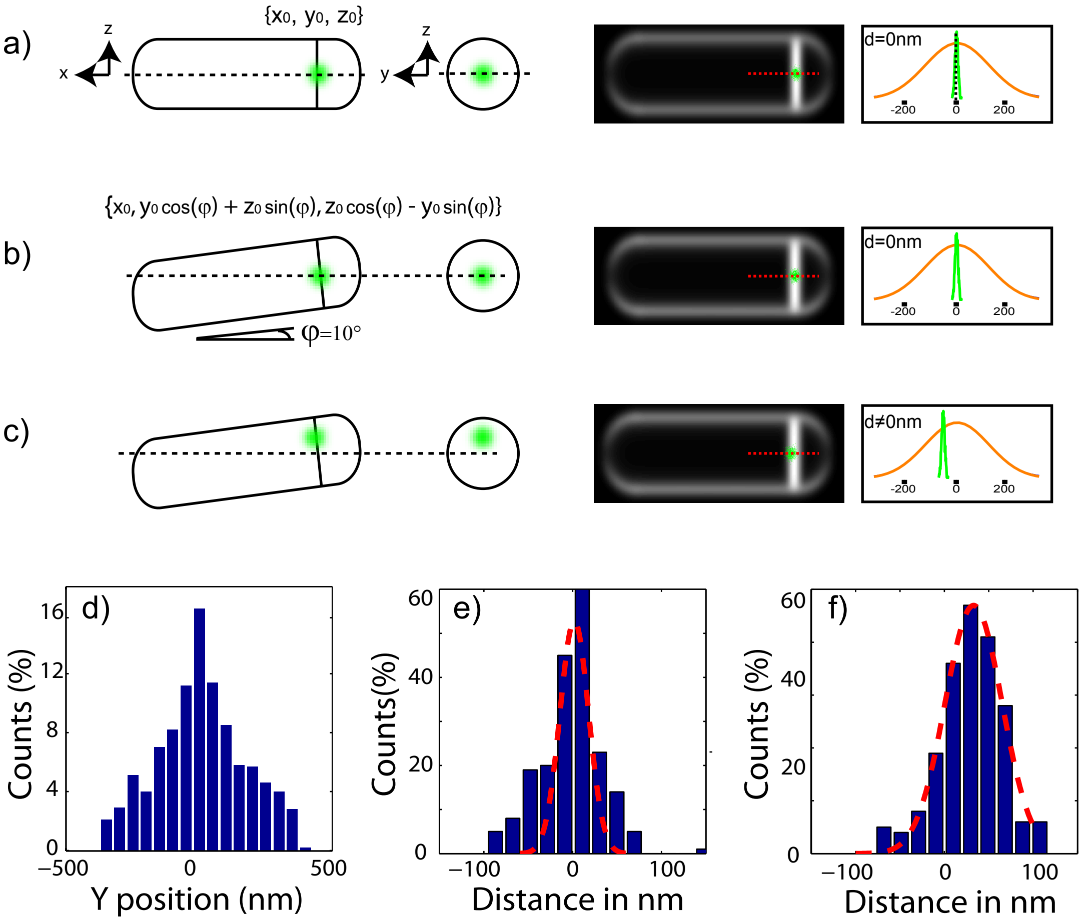

We performed simulations to test the effect of tilt of the cell axis with respect to the optical axis (φ) in the localization of single-emitters with respect to the septal membrane. From these simulations, we can conclude that our measurements of distances of SpoIIIE clusters to the centre of septa were not affected by tilt.

Simulated epifluorescence images of the cell membrane are calculated using an algorithm written in Matlab (Mathworks, version 2011a). We assume that the dye staining the membrane is homogeneously distributed along the bacterium membrane, cell wall and sporulation septum. The width of the bacterium was considered to be 800 nm and its length 2 μm. The microscope point spread function was assumed to be Gaussian, with a lateral standard deviation $\sigma_{x\text{-}y}$ of 250 nm and an axial standard deviation σ_z of 500 nm. The cluster of SpoIIIE proteins was simulated as an ensemble of 600 fluorescent events following a Gaussian distribution with a standard deviation σ of 30 nm. Then, we used our distance determination algorithm (Supplementary Methods) to recover the distribution of distances of single-emitters to the center of the septum.

(a) For the first simulation, the cluster of SpoIIIE proteins is placed at the centre of the sporulation septum (*yz* plane) and on the image plane (*xy*). (middle panel) Simulated

image of the cell (white representing membrane stain) and the cluster of SpoIII localizations (green). (right panel) As expected, after applying our distance measurement algorithm the distribution of SpoIIIE localizations (green curve) is perfectly centered with the membrane fluorescence intensity distribution across the septum (orange curve). *d* indicates the distance between the center of SpoIIIE cluster and center of membrane.

(**b**) If a tilt of 10° is added without changing the position of the cluster or the image plane, almost no change is observed on the simulated image of the cell, though the sporulation septum appears slightly thicker. For tilts $\varphi < 10°$, the distance *d* between the SpoIIIE and membrane intensity distributions remains equal to zero, and therefore does not affect our measurements of distance of the cluster to the center of the septum. For values above 15°, the simulated images start to show membrane distribution artifacts that are clearly related to a large tilt and are not observed experimentally. This simulated result suggests that the tilt angle on the sample surface remains below 15° in our experiments. Consistent with this observation, 3D-SIM imaging showed that our protocol for fixing cells in our micro-fluidics chambers produces samples with cells lying flat on the surface with a tilt smaller than 5°. Thus, in our observation conditions, our measurements are not affected by our experimental tilt.

(**c**) To explore the influence of the septal membrane (yz plane) positioning of the SpoIIIE cluster, we simulated a cell tilted by 10° and a SpoIIIE complex outside the image plane. In this case, the cluster will appear shifted from the sporulation septum on the simulated epi-fluorescence image (middle panel), even though the proteins are actually assembled on the septum. Under these conditions, a tilt of 10° and a cluster 50 nm away from the septum centre can lead to a shift as high as ± 8 nm along the x direction, depending on the position of the complex along the z axis (right panel). The detection of single-molecules and the stringent fit of the PSF to a gaussian distribution creates a depth-of-field in PALM imaging that is smaller than a few tens of nanometers, thus a shift of 8nm would be an upper bound (i.e. maximum expected systematic error).

In order to ensure that this potential systematic error was not influencing our measurements, we investigated the effect of tilt and septal plane positioning on the average cluster-to-septum distance after a series of 150 independent simulations (each representing a single cell with a specific φ angle and a cluster at a specific *yz* position) in which:

1- The radial and angular positions of the cluster in the septal plane (*yz*) are homogeneously distributed

2- The tilt angle follows a normal law centered around zero with a standard deviation σ_φ of 10°

(d) As expected from the simulation conditions, the distribution of cluster positions on the septal plane (plotted as a distance distribution in the y-axis) is centered at zero and shows a large dispersion. (**e**) Importantly, despite this large dispersion of cluster positions on the

septal plane, the distances of detected SpoIIIE clusters to the centre of the sporulation septum shows a gaussian distribution with a mean of zero and a standard deviation of σ=35 nm. Thus, provided that a large enough sample is used, our method recovers the mean distance of SpoIIIE clusters to the septum in spite of changes in the cell axis with respect to the surface (for angles φ <~10 °) and heterogeneous distributions of SpoIIIE clusters on the septal plane.

(f) Finally, we simulated a distribution of clusters located at a distance x_0 = 30 nm away from the septum in which we randomly varied the position of clusters in the *yz* plane and the angle φ as in (**d-e**). Importantly, the distribution of cluster-to-septum distances gives a mean distance <*d*> = 32 ± 20 nm (s.d.), thus recovering the simulated results despite large dispersions in the *yz* distributions and φ angles.

From these simulations, we conclude that the presence of a gaussian distribution of tilt angles around φ = 0° (flat) in a population of cells changes the standard deviation of localization measurements but, importantly, not the mean value of the distribution. The dispersion in localization measurements increase with tilt, highlighting the need for an experimental method in which cells are kept as flat as possible and a large number of measurements are made in order to obtain an accurate value for the localization of SpoIIIE clusters.

Thus, for experiments performed in micro-fluidics chambers, the influence of tilt in measurements is minimal (as cells were flat within a few degrees and the surface was highly stable). In SpoIIIE localization measurements performed in agar pads (Supplementary Fig. 11d), the dispersion was higher (σ~60 nm) than that observed in micro-fluidics chambers (σ~30 nm), likely due to the surface of the pads being not perfectly flat, leading to larger variations in cell orientations.

Bibliography

1. Lee SH, Shin JY, Lee A, Bustamante C (2012) Counting single photoactivatable fluorescent molecules by photoactivated localization microscopy (PALM). Proc Natl Acad Sci U S A 109: 17436-17441.
2. Annibale P, Scarselli M, Kodiyan A, Radenovic A (2010) Photoactivatable Fluorescent Protein mEos2 Displays Repeated Photoactivation after a Long-Lived Dark State in the Red Photoconverted Form. The Journal of Physical Chemistry Letters 1: 1506-1510.
3. Wiedenmann J, Ivanchenko S, Oswald F, Schmitt F, Röcker C, et al. (2004) EosFP, a fluorescent marker protein with UV-inducible green-to-red fluorescence conversion. Proceedings of the National Academy of Sciences of the United States of America 101: 15905-15910.
4. Cormack BP, Valdivia RH, Falkow S (1996) FACS-optimized mutants of the green fluorescent protein (GFP). Gene 173: 33-38.
5. Mika JT, Poolman B (2011) Macromolecule diffusion and confinement in prokaryotic cells. Curr Opin Biotechnol 22: 117-126.
6. McEvoy AL, Hoi H, Bates M, Platonova E, Cranfill PJ, et al. (2012) mMaple: A Photoconvertible Fluorescent Protein for Use in Multiple Imaging Modalities. PLoS One 7: e51314.
7. Ferguson ML, Le Coq D, Jules M, Aymerich S, Declerck N, et al. (2011) Absolute quantification of gene expression in individual bacterial cells using two-photon fluctuation microscopy. Anal Biochem 419: 250-259.
8. Digman MA, Dalal R, Horwitz AF, Gratton E (2008) Mapping the number of molecules and brightness in the laser scanning microscope. Biophys J 94: 2320-2332.
