# Supplementary material for: Recruitment, Assembly, and Molecular Architecture of the SpoIIIE DNA Pump Revealed by Superresolution Microscopy
Source: PLoS Biol. 2013 May 7;11(5):e1001557. doi: 10.1371/journal.pbio.1001557 (PMC3646729; doi:10.1371/journal.pbio.1001557)
Supplement: Methods S1 — (SM1) PALM microscope setup. (SM2) Coverslip cleaning protocol. (SM3) Cell culture, strains and induction of sporulation. (SM4) Fluidic chamber assembly. (SM5) In vivo PALM imaging with fluidic chamber. (SM6) In vivo PALM experiments on agar pads. (SM7) Alignment correction in PALM experiments. (SM8) Drift correction for PALM experiments. (SM9) Contour calculation and automatic cell sorting. (SM10) SpoIIIE pointillist reconstruction and cluster detection for PALM experiments. (SM11) Calculation of distance between SpoIIIE proteins and the membrane. (SM12) 3D structured illumination microscopy (3D-SIM). (SM13) Two-photon number and brightness analysis. (SM14) Calculation of cluster localization probability. (PDF) [file pbio.1001557.s014.pdf]

Recruitment, assembly and molecular architecture of the SpoIIIE DNA pump revealed by super-resolution microscopy

Fiche, JB., Cattoni, DI., Diekmann, N., Langerak, J., Clerte, C., Royer, C.A., Margeat, E., Doan,T., Nöllmann, M.

Supplementary Methods (SM)

SM 1. PALM microscope setup **2**

SM 2. Cleaning protocol for the coverslips **4**

SM 3. Cell culture, induction of sporulation, and strains **4**

SM 5. In-vivo PALM imaging with fluidic chamber **7**

SM 6. In-vivo PALM experiments on agar pads **9**

SM 7. Alignment correction in PALM experiments **10**

SM 8. Drift correction for PALM experiments **11**

SM 9. Contour calculation and automatic cell sorting **12**

SM 10. SpoIIIE pointillist reconstruction and cluster detection for PALM experiments **13**

SM 11. Calculation of the distance between SpoIIIE proteins and the membrane **15**

SM 12. 3D Structured Illumination Microscopy (3D-SIM) **18**

SM 13. Two photon Number and Brightness analysis (N&B) **18**

SM 14. Calculation of cluster localization probability maps (heat maps) **19**

Supplementary Table 1. Equipment parts **20**

Supplementary Table 2. Products and consumables used for the PALM experiments **21**

Supplementary Table 3. Bacterial strains used in this work **21**

Bibliography **22**

SM 1. PALM microscope setup

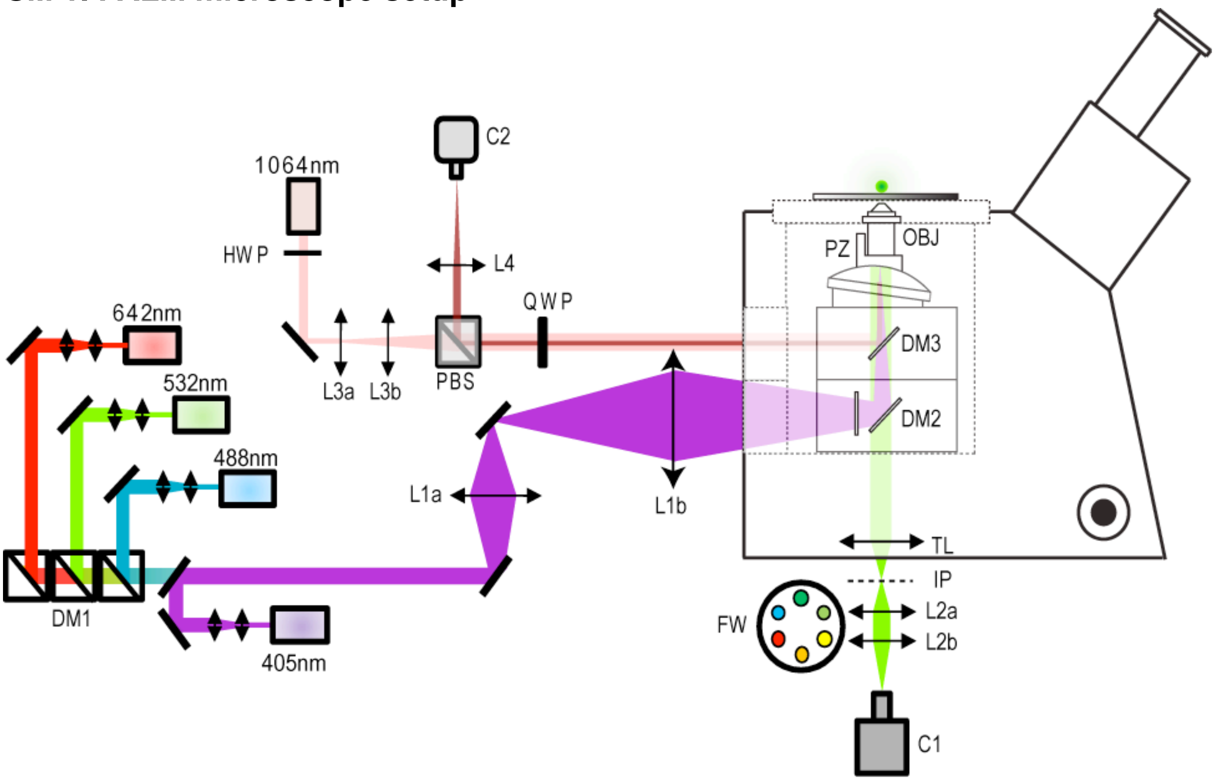

Four lasers with excitation wavelengths of 405nm, 488nm, 532nm and 642nm were expanded to a beam diameter of 2mm and combined into a single co-linear beam using dichroic mirrors (DM1). Two achromatic lenses (L1a & b) were used to expand the excitation beam and to obtain a homogeneous illumination over fields of view as large as 40x40µm². The lasers were focused by L1b, located near the back port of the microscope, and directed by dichroic mirror DM2 to the back focal plane of a 100x Plan-Apo oil objective (OBJ, NA = 1.4) mounted on a z-direction piezoelectric stage (PZ). L1b can be translated perpendicularly to the optical axis in order to shift between TIRF and epifluorescence imaging mode, depending on the experiment. A motorized stage is used to translate the sample perpendicularly to the optical axis.

Fluorescence from the sample is collected by the objective, separated from laser light by dichroic mirrors DM2 and DM3 and focused by the tube lens (TL) on the microscope original imaging plane (IP). A pair of achromatic relay lenses arranged as a telescope (L2a & b) is used to form an image on the EMCCD camera sensor (C1) while increasing the total magnification of the system (effective pixel size of 115nm). A motorized filters wheel (FW) placed between the two lenses allows for the selection of the fluorescence emission filter.

Control software for the lasers and the filter wheel was written in LabView 2010 (National Instrument, France). The emission intensity of each laser is controlled individually using a USB-DAQ device (National Instruments). Specific electronic circuits were designed for the control of the 532nm laser.

To avoid loss of focus during PALM acquisition, an active autofocus system was built. In a separate path from the other four lasers, a linearly polarized 1064nm IR beam from an Ytterbium fiber laser is expanded twice (L3a & b) and passes through an optical separator formed by a polarized beam splitter (PBS) and a quarter wave plate (QWP). The beam is directed towards the objective lens by dichroic mirror DM3. Depending on the sample and the position of the plane imaged by the objective, the distance between L3a and L3b can be modified to ensure that the IR beam is always focused at the glass/sample interface. Part of the IR beam is reflected by the sample, collected by the objective and redirected towards the PBS following the same path than the incident beam. Due to a change in polarization introduced by reflection at right angles, the reflected beam is redirected by the PBS towards lens L4 and imaged on a CCD detector (C2). A half-wave plate (HWP) is used to manually adjust the intensity of the incident beam.

Control software for the autofocus was written in LabView 2009, using the PID and Fuzzy Logic Toolkit. A feedback loop between the CCD detector (C2) and the piezo stage (PZ) makes sure that the sample remains in focus at all times during the PALM acquisition (resolution of ~5-10 nm over hours). At the beginning of each experiment, a calibration is carried out to ensure that the intensity of the IR reflection varies linearly over a course of 600nm around the plane imaged by the objective. When the acquisition starts, the intensity of the IR reflection is used as reference and axial drift is corrected by adjusting the position of the objective.

Information on specific part numbers and vendors for all key components is given in **supplementary Table 1 (below)**.

SM 2. Cleaning protocol for the coverslips

Coverslips were rinsed sequentially with Acetone, Methanol and MilliQ-Water, then placed in 1M KOH solution and sonicated for 30 minutes in a Branson 2510 sonicator. Afterwards, the slides were rinsed and stored in MilliQ-water. Before an experiment, the coverslips were dried by flushing nitrogen and passed over an open flame to eliminate any remaining fluorescent contamination.

SM 3. Cell culture, induction of sporulation, and strains

Cells were plated directly from glycerol stocks and kept overnight at room temperature. Next day, the plate was incubated at 37°C until single colonies were observed. For optimal aeration, 250ml flasks were used for day cultures and sporulation. To induce sporulation, *B. subtilis* strains were grown at 37°C at 200 rpm to O.D. ~0.6 in 20% LB medium and induced to sporulate by resuspension at 37°C [1] in sporulation medium (see below). For strains expressing a fluorescent tag, flasks were covered with aluminum foil to avoid light exposure. For experiments in agar pads, we added sporulation supernatants, which affected previous long-term time-lapse imaging of the process of sporulation [2].

GFP fusions used *gfp*-mut2, a monomeric mutant of GFP that folds within minutes and provides high brilliance [3]. Fusion of GFP to the carboxyl terminal of SpoIIIE was achieved by integration of pEB410 [4] in PY79 by a single recombination event at spoIIIE and selected for Spo+ a phenotype to obtain SpoIIIE-GFP [4]. The DNA sequence of the linker between SpoIIIE and fluorescent reporters was CTC GAG GGT TCC GGA. GFP was replaced by eosFP by cutting *gfp* and ligating with the *eosFP* gene at the SpeI site to produce pEB428. To obtain EB1407, PY79 was transformed with pEB428 by a single recombination event and selected for a Spo+ phenotype to get SpoIIIE-eosFP. Strains were verified by sequencing. SpoIIIE-maple and SpoIIIE-GFP fusions in BTM200 were generated by isothermal assembly [5] of PCR products followed by direct transformation into *B. subtilis*. SpoIIIE fusion strains grew at the same rate as wild-type *B. subtilis* (PY79), and showed similar sporulation levels than PY79 in spore titers assays. For more details on strains, see Supplementary Table 1 (below).

<u>*Sporulation medium protocol (adapted from Sterlini et al. [1])*</u>

Solution A - per 60 ml: 0.9 ml 2% $FeCl_3.6H_2O$, 0.83 g $MgCl_2.6H_2O$, 2.0 g $MnCl_2.4H_2O$, Filter sterilize and store at 4°C.

Solution B - per 100 ml. 5.35 g NH_4Cl, 1.06 g Na_2SO_4, 0.68 g KH_2PO_4, 0.97 g NH_4NO_3, Adjust pH to 7.0 with NaOH and bring volume to 100 ml. Aliquot and autoclave. Store at 4°C.

Pre-sporulation medium

-50 ml MOPS 0.5M ph7.5
-1ml A
-10 ml B.

-920 ml with doubly-distilled dH_2O
-adjust ph to 7.2-7.4
-aliquot and filter in 92ml fractions, keep at 4C.

Sporulation medium
-92 ml pre-sporulation medium
-2 ml 10% Glutamic Acid
-1 ml 0.1M $CaCl_2$
-4 ml 1M $MgSO_4$

SM 4. Fluidic chamber assembly

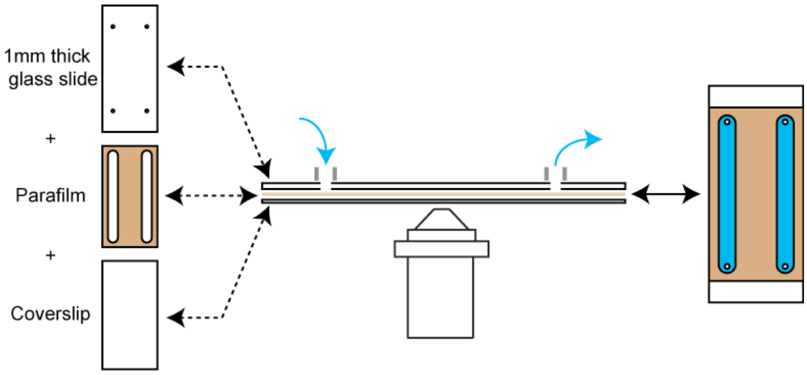

Fluidics chambers consisted of a 1mm thick glass slide with two pairs of inlet/outlet and a coverslip, sealed together by melted parafilm. As depicted in the figure, inlet/outlet ports were drilled through the 1mm thick glass slide using a laser cutter (Thermoflan, France). A thick glass slide was used to ensure a better rigidity of the chamber and to avoid deformation when approaching the objective lens. The two channels were cut on a small piece of parafilm and sandwiched between the glass slide and the coverslip, making sure each channel is properly aligned with a pair of inlet/outlet. The chamber was subsequently sealed by heating the assembly at 90°C for 1min.

Prior to injection of bacteria, each channel was filled with a 0.01% (v/v) solution of poly-L-Lysine and incubated for at least 5 minutes at room temperature. In the meantime, 1ml of bacterial suspension was spun down and resuspended in 100µl of filtered sporulation medium. Cells were injected and let settle onto the poly-L-Lysine coated surface for several minutes. A high flow force was applied by pumping up several milliliters of medium at ~200 µl/s to rinse the channel and make sure the bacteria were properly attached to the surface.

Fluorescent beads were added to the bacterial solution and injected in the chamber. Immobilized beads were used as fiducial marks for drift correction. The ratio between bacteria and beads was adjusted to obtain an optimal density of 5-15 beads per field of view. In the end, 100-200 bacteria can be imaged on each field of view, all of them being well separated and laying flat on the surface.

SM 5. In-vivo PALM imaging with fluidic chamber

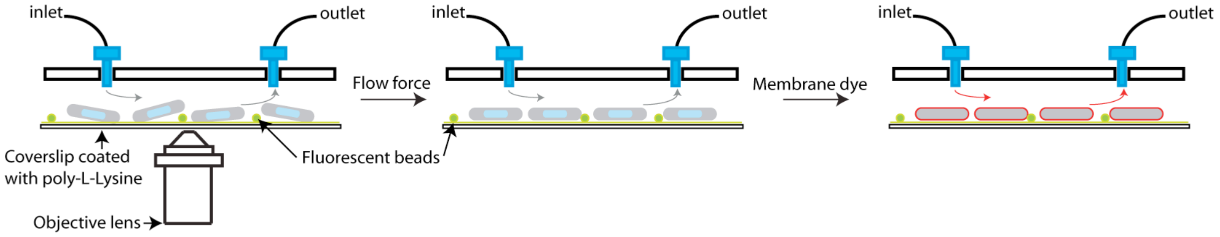

Sporulating cells were imaged using the epifluorescence imaging configuration previously described. All the images are acquired using the same four-band dichroic filter. First, DNA stained with sytox green was imaged by exciting at 488nm and collecting the emitted fluorescence with a 525±25nm band pass filter (Chroma). A well contrasted image of bacterial chromosomes was obtained and will be used later to calculate the cell contours (see **Contour calculation and cells sorting** for more detailed explanations). DNA stain was used for the initial segmentation as it is more robust than membrane stain. In fact, DNA stain images of cells in physical contact will show a gap in fluorescence signal between them, whereas the membrane stain of both cells will overlap at the contact point, making segmentation more difficult. Fluorescent beads were imaged as well and their positions calculated to correct for drift and chromatic aberrations introduced when changing emission filters.

The focal plane was located at the center of cells at the beginning of the experiment by using phase contrast images, and confirmed by fluorescence imaging on sytox-labeled DNA. This focal plane was conserved during the whole data collection process by the use of an accurate autofocus system, which maintained the focal plane position within 5-10 nm over hours. Cells lied flat on the microfluidics surface (Supplementary Fig. 8), therefore the focal plane position was identical in all cells.

PALM imaging of SpoIIIE was performed immediately after, using a 605±35nm bandpass filter (Chroma). Between 10.000 and 25.000 images were acquired under continuous illumination of the 532nm read-out laser with a typical power density on the sample of 0.2kW.cm^{-2}. This value was optimized for the detection of single photo-activatable proteins while preventing activation induced by the read-out laser and photo-damages to the cells. Exposure time was set to 55ms in frame transfer mode and electronic gain was set between 300 and 1000. Pulses of the 405nm laser were used for photo-activation of single emitters and their length and power were slowly increased during the course of the experiment in order to maintain the density of activated fluorophores constant. Typical starting values for the pulse length and power were 5ms and 10W.cm^{-2} respectively.

When all the fluorescent proteins have been activated and photo-bleached, 100µl of a 10nM membrane stain solution (FM4-64) was slowly injected in the fluidic chamber.

Bacterial membranes were imaged by exciting with the 532nm laser and using the same emission filter, thus preventing chromatic aberration between PALM images and membrane images.

SM 6. In-vivo PALM experiments on agar pads

A double-side adhesive o-ring (Invitrogen) was placed on a coverslip. 60µl of 2.4% melted agarose (diluted in PBS, melted at 95°C) were spread on the centre of the coverslip, which was then dried for 12 minutes in a sterile vertical laminar flow-hood. The high concentration of agarose was used to improve the stability of the pad during PALM experiments. However, the agarose must melt slowly without reaching its boiling point in order to prevent high auto-fluorescence background during PALM experiments.

In the meantime, cells were stained by adding 5µl of a 0.3µM solution of membrane dye (FM4-64) to 1ml of bacterial suspension. After five minutes incubation avoiding light exposure, the bacterial aliquot was spun down in a bench centrifuge at 24°C and 4000rpm for 3 minutes, the supernatant discarded and the pellet resuspended in 60µl filtered sporulation medium. Fluorescent beads were added to the suspension and 3ul of the bacterial solution is pipetted onto the agar. The pad was then sealed with a clean coverslip and kept in the dark for three minutes to allow for spreading of the bacteria over the surface. The sample was finally fixed into an Attofluor cell (Invitrogen) to avoid bacterial motion during microscopy.

PALM acquisition conditions for pads were the same than for micro-fluidics chambers. The image of the membrane was acquired at the very beginning of the experiment instead of at the end.

PALM localizations and bead-specific fluorescence events were calculated using MTT-analysis software [6]. Bacteria contour detection and calculation were done with microbeTracker [7]. Further analysis of PALM experiments on bacteria were performed using PALMcbs, a custom-made software written in Matlab 2011.

SM 7. Alignment correction in PALM experiments

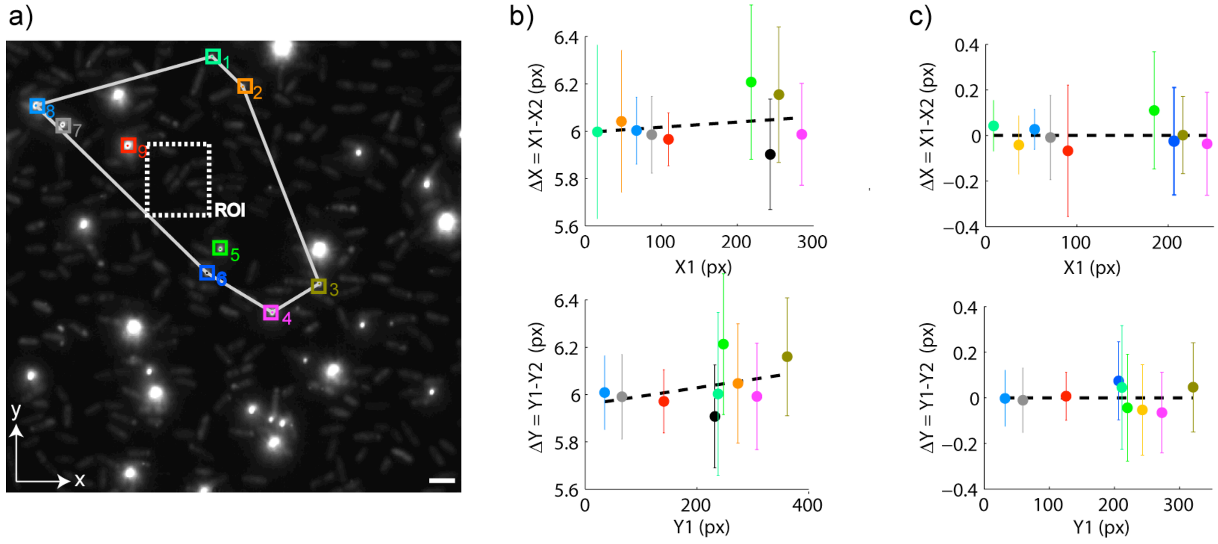

Misalignment and chromatic aberrations between images obtained at different times and using different filters were corrected using the positions of fluorescent beads scattered over the field of view (**a**). For each two sets of images, the mean positions (X_i and Y_i) and the localization errors (σ_{Xi} and σ_{Yi}) were calculated for each bead. For a given ROI (**a**), a minimum of seven proximal beads were automatically selected and a second-order polynomial transformation T was inferred from their positions.

This transformation allowed for the correction of image translation and rotation as well as variation in magnification. The quality of the alignment was assessed by comparing the variation in the bead positions between two images, before and after applying the correction (**b** and **c,** respectively). Typically, a shift of several pixels in bead positions (ΔX and ΔY) can be usually measured between both images when no correction was applied. This is illustrated on figure **b** (upper and lower panels) where the values of ΔX and ΔY measured for each bead were plotted as a function of the positions X_1 and Y_1 respectively. In this particular example, an average variation of six pixels was measured between both images and along the two axes. (**c**) After applying the polynomial transformation, both ΔX and ΔY values fell below 0.1 pixels (i.e. 11.5nm for our pixel size), illustrating the quality of the alignment correction.

Scale bar is 3µm.

SM 8. Drift correction for PALM experiments

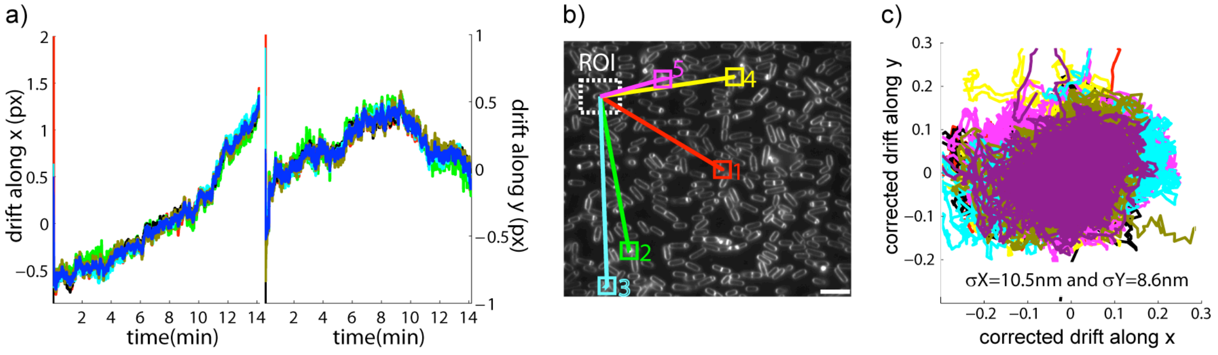

Lateral drift over the full acquisition period was assessed by plotting the trajectories of fluorescent beads in x and y coordinates over time (**a**). For this procedure, only beads detected during the entire acquisition (~15-20 minutes) were employed. Curves were smoothed by a Stavinsky-Golay filter and overlaid by minimizing the distance between each trajectory using the first selected bead as reference. The origin was calculated by averaging the trajectories over the first 100 images, ensuring the drift was equal to zero at t=0min. For each ROI, the trajectories of five close-by beads were chosen (**b**) and a reference trajectory was calculated (blue curve on **a**). The quality of the drift correction was estimated by subtracting the reference to all the trajectories and calculating the standard deviations along x and y (**c**). Experimentally, we found that the drift correction precision was 10nm for the experiments performed on poly-L-Lysine and 15nm for experiments performed on agar pads. Scale bar is 6µm.

SM 9. Contour calculation and automatic cell sorting

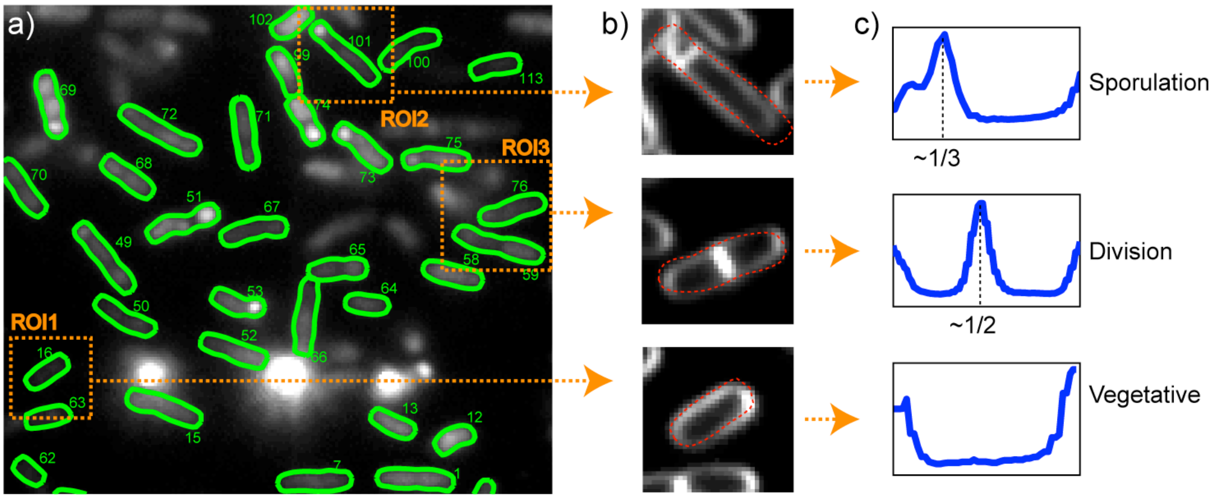

After imaging DNA stained with sytox green, cell contours were calculated using a modified version of MicrobeTracker (Sliusarenko et al. 2011). For each detected bacterium, the position of the cell and the points delimiting its contour were saved and fed to our PALMcbs software (**a**).

Due to misalignment between the DNA and membrane images, each contour was automatically recalculated to make sure it was properly aligned with the image of the cell wall (**b**). Since bacterial chromosomes were naturally confined within the cell, the contour calculated with microbeTracker was often too small to encompass the whole membrane and required enlargement.

Finally, a cell sorting procedure was used to classify bacteria according to the three growing states studied in this work: *vegetative/pre-divisional, dividing* or *sporulating* (**c**). The presence of a septum was first assessed by plotting the fluorescence intensity profile of the membrane along the cell length. If no septum was detected, the bacterium was automatically classified as *vegetative/pre-divisional*. Else, the cell was divided into three compartments of equal length. If the septum was found in the middle one, the cell state was set to *division*. Otherwise, if the septum was found in one of the two other compartments, the bacterium state was set to *sporulating.*

The automatic cell sorting procedure was correct in 90% of the cases, with 10% of cells mis-classified due to fluorescence inhomogeneities in membrane stain and cell-cell contacts. Consequently, for each bacterium, its state was manually verified (and re-assigned if necessary) to prevent any errors in the interpretation of the data.

SM 10. SpoIIIE pointillist reconstruction and cluster detection for PALM experiments

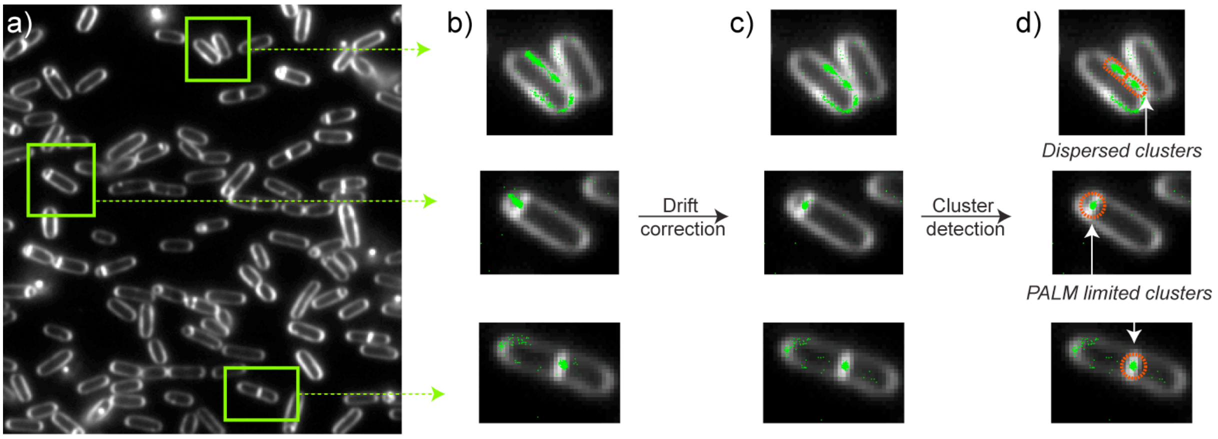

PALM experiments were analyzed using MTT-analysis [6]. For each image acquired, PALM localization events (or single-molecule events) were localized and their coordinates fed to PALMcbs. In our PALM images, each single-molecule event obtained from a single frame is represented by a single green (or colored) dot. To obtain a pointillist representation, all the single-molecule events obtained from all frames are plotted together, and over-imposed on the membrane stain image in white. Single photo-activated proteins can emit over several contiguous frames, therefore generating many single-molecule events. In average, we observe ~60 single-molecule events per single photo-activated protein (described in Supplementary Fig. 3). In a pointillist image, we represent each single-molecule event by a point. In the images shown in Figure 2a-b, PALM-limited clusters are the overlap of several (in average ~36) single proteins and so contain an average of ~2000 single-molecule events (see also Supplementary Fig. 3).

From a pointillist PALM image of the whole field of view (**a**), a cell was automatically selected, and (**b**) the fluorescent events detected in a 50- to 70-pixels ROI centered on the bacterium of interest were extracted. (**c**) Lateral drift was corrected making sure that the localization of SpoIIIE proteins is within the cell and that no false-positive events were detected outside the cell.

Secondly, the distribution of fluorescent events was analyzed in order to find clusters of SpoIIIE proteins in the bacterium (**d**). This detection is only based on the spatial organization of the fluorescent events and do not take into account when and for how long events were detected during the acquisition. A first rough analysis was carried out and returned the positions of all the clusters detected in the cell, starting with the largest ones. Since SpoIIIE complexes were sometimes dynamic, the events associated to a single complex could be dispersed over large distances along the membrane (see for example the cell in vegetative state, in the top of panels b-d), leading to the detection of several smaller clusters instead of a single large one.

To prevent this artifact, the area surrounding each cluster (C_i) was probed in order to check if other fluorescent events or close-by clusters could be connected to C_i. This was achieved by increasing the radius of the searching area by steps of 0.5 pixels (~55nm) around the cluster C_i. If the number of events detected in the enlarged area increased by more than 5%, all the events were connected together to form a single cluster C_i and the search proceeded with the next area. However, if the variation in the number of events is lower than 5%, the search was stopped and another cluster C_{i+1} was analyzed.

If a cluster contained less than 50 events, it was automatically discarded since the number of events is too low to obtain accurate localization statistics. Otherwise, its size is calculated using the standard deviation $\sigma_{x,y}$ of the spatial distribution. If $\sigma_{x,y}$<40nm (<100 FWHM, the cluster is defined as a “PALM-limited cluster” and if $\sigma_{x,y}$>40nm (>100 nm FWHM) it is designated as a “dynamic cluster”. The cut-off value of 40nm was chosen empirically after analyzing the size of SpoIIIE proteins clusters detected at the sporulating septum in ~150 bacteria and the number of particles per cluster (see Fig. 2b).

The overwhelming majority of detections were either in PALM-limited or dynamic clusters (>95%). Cells with fewer than 10 events were classified as empty, and cells in which all clusters had less than 25 events were also classified as empty.

SM 11. Calculation of the distance between SpoIIIE proteins and the membrane

1. PALM using fluidics chamber

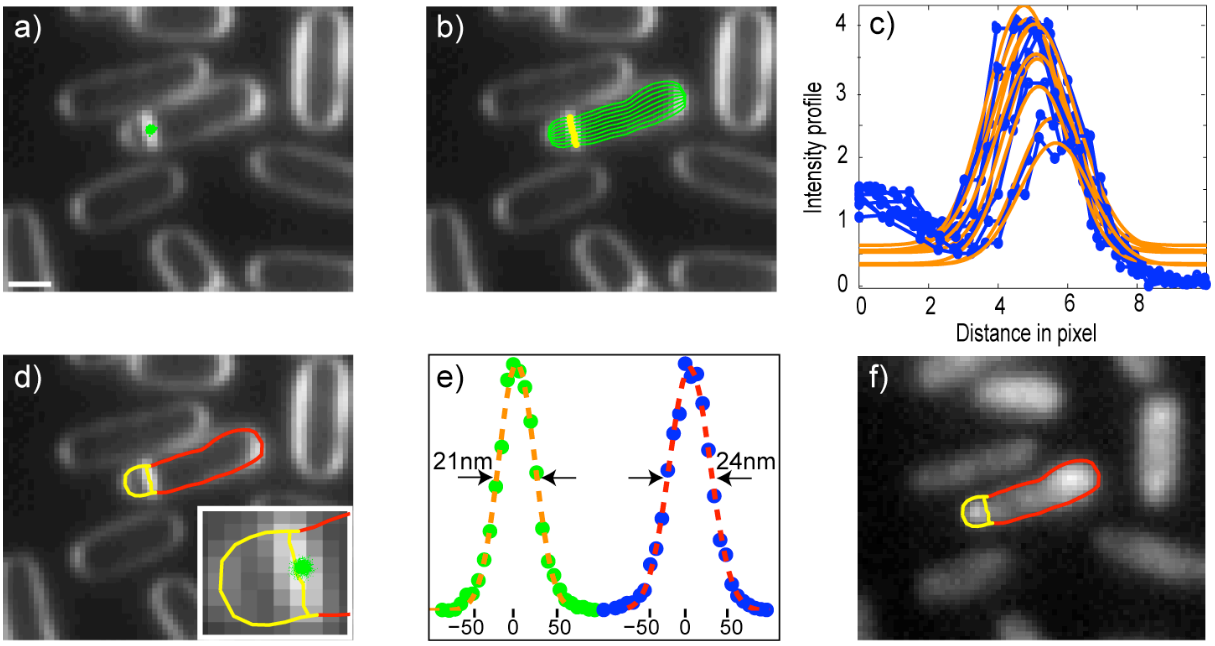

This calculation method was used for experiments performed in micro-fluidics chambers (i.e. the majority of the data presented in the paper).

(**a**) After selecting a bacterium of interest and applying all the necessary corrections to the SpoIIIE specific fluorescent events, (**b**) nine equally spaced lines (green) were plotted across the cell contour, each of them crossing the sporulation septum at a different location. (**c**) An intensity profile of the septum was generated for each line (blue lines) and the position of the centre was calculated using a Gaussian fit (orange lines on **c**). If the quality of the fits was good enough ($r>0.9$), the calculated positions (yellow dots on **b**) were used to interpolate a profile of the sporulation septum. (**d**) By combining the septum profile with the cell contour, the forespore and mother cell compartments were automatically delimited and a contour of both compartments was generated (yellow and red contours, respectively). The two contours were then used to analyze the position of the SpoIIIE cluster within the cell and calculate its distance with respect to the sporulation septum (inset on **d**).

(**e**) Additionally, the distributions of SpoIIIE localizations in the directions perpendicular and parallel to the septum were calculated (blue and green dots respectively). These two distributions were used to estimate the size of the SpoIIIE cluster and its aspect ratio. On the figure, the standard deviation is indicated (21 nm for the direction perpendicular to the septum and 24 nm for the perpendicular direction). The size of SpoIIIE clusters is defined as the Full Width Half Maximum (FWHM) value.

(**f**) Finally, the percentage of chromosome already translocated in the forespore is calculated by the ratio of total fluorescence intensity emitted by the DNA marker in the forespore (yellow compartment) by that in the mother cell (red compartment). Thus, after full packaging of one chromosome in the forespore, the percentage of DNA translocated reaches 100%. Calculations are performed after correcting for background intensity.

Scale bar is 1µm.

The number of cells with a large proportion of DNA transferred into the forespore is under-represented due to two main reasons: (1) we selected cells that have a strictly flat septum to avoid any systematic error in the localization of the relative distance of a SpoIIIE cluster to the center of the septum. At the end of DNA translocation (which coincides with the beginning of engulfment), septa tend to be curved and thus there are fewer cells with a large amount of DNA translocated; (2) The maximum percentage of DNA inside the forespore may be underestimated due to quenching of SYTOX green at the highest DNA concentrations at the end of DNA translocation.

2. PALM on agar pads

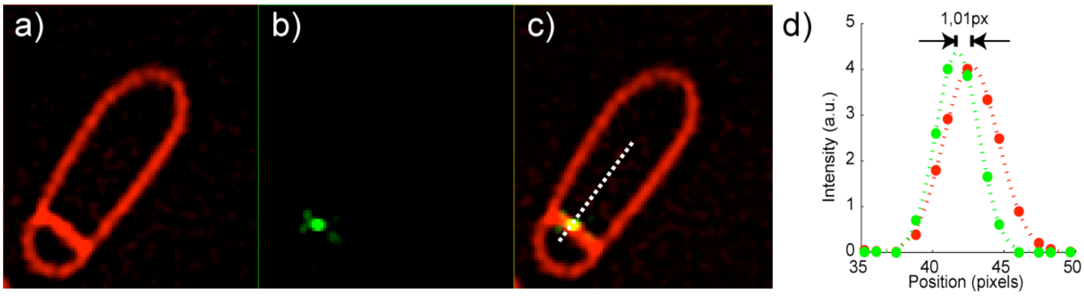

This calculation method was used for experiments in which cells were immobilized in agar pads (Supplementary Data 11c-d).

(**a,b**) A bacterium of interest was selected and all previously described corrections were performed (membrane in **a**, protein in **b**). (**c**) A line perpendicular to the septum and going through the center of the SpoIIIE cluster was manually placed. (**d**) Average intensities of all the pixels along the perpendicular line were used to generate an intensity-profile of the septum (red dots), which was then fitted with a Gaussian distribution to determine the position of the centre of the septum (red dotted line).

Next, all SpoIIIE-specific single-molecule localization events were projected onto the perpendicular line. A distribution of the positions was generated and the average position of the SpoIIIE cluster was estimated using a Gaussian fit (green dotted line). The size of the cluster is estimated as well calculating the standard deviation of the distribution of single molecule events.

Finally, the distance between the septum and SpoIIIE cluster was calculated by measuring the distance between the maxima of the two distributions. This procedure was repeated 6 times for each PALM-limited cluster in order to reduce statistical uncertainties introduced by manually placing the perpendicular line. These measurements were used to calculate an average SpoIIIE/septum distance and a standard deviation.

SM 12. 3D Structured Illumination Microscopy (3D-SIM)

Sample preparation was done as described for PALM-imaging in a micro-fluidics chamber. Cells are attached on poly-lysine coated, high precision #1.5H coverslips (Marienfeld GmbH) and the sample mounts on the OMX acquisition stage.

3D-SIM imaging was performed on a OMX V3 microscope (Applied Precision Inc.) using the 488nm and 561nm lasers and the corresponding standard drawer. Reconstruction and alignment of the 3D-SIM images was performed using softWoRx v 5.0 (Applied Precision Inc.). 100nm green and red fluorescent beads (Invitrogen) were used to measure the experimental optical transfer functions for the corresponding channels. z-stacks for 3D reconstruction were of 170nm. TetraSpeck beads (Invitrogen) were used to measure x, y and z offsets, rotation about the z-axis and magnification differences between channels. These corrections were applied to the acquired images.

SM 13. Two photon Number and Brightness analysis (N&B)

In the laser scanning module, 930 nm excitation light was expanded to fill the back aperture of a Zeiss Apochromat 63X, 1.4 NA, oil immersion objective. Laser power before injection into the laser scanning module was 25 mW. Infrared excitation light was filtered from the detection path by an E700 SP-2P dichroic filter and a secondary E700 SP-2P filter (Chroma Technologies) placed between the laser scanning module and the detector. Fluorescence signal was detected through a bandpass filter 525/70-2p (Chroma Technologies). A series of 50 raster scanned 20 µm × 20 µm images of 256 ×256 pixels were recorded with a 100 µs laser dwell time per pixel, chosen to be faster than the GFP diffusion rate and to provide statistically relevant photon counts while reducing the effects of photobleaching.

Image stacks were analyzed for number and brightness (N&B) values using SimFCS program. A detailed explanation of the N&B analysis methods can be found in Digman et al. [8]. In scanning N&B analysis, the number and molecular brightness of the diffusing fluorescent molecules are calculated from the fluctuations in fluorescence intensity at each pixel in a series of raster scanned images, in which the laser dwell time is small with respect to the diffusion time of the molecule. Fluorescence fluctuations from the average intensity arise from Brownian diffusion into and out of the very small two-photon excitation volume focused inside the bacterial cells. The pixel-based temporal average and variance images were calculated from 50 raster scans. The average brightness for a population of cells expressing monomeric freely diffusing GFP under the same set of conditions as the experiments was obtained. The average brightness were calculated and normalized by the brightness of a monomeric GFP for the pixels corresponding to the foci formed by SpoIIIE inside the bacteria in vegetative or sporulation conditions.

SM 14. Calculation of cluster localization probability maps (heat maps)

Heat maps illustrate the probability distribution of SpoIIIE cluster localization in the cell for a given stage and a specific cluster type. Cells are analyzed separately, according to the following procedure:

1- The relative positions of each single-molecule localization event within the reference coordinate system of the cell is calculated. Single-molecule coordinates are normalized to the length and the width of the bacterium from which they were detected.
2- Events are placed into the first quartile of the cell and then reflected into the other three quartiles by enforcing mirror symmetry along the long and short axis of the cell. Since the number of events varied significantly from one bacterium to the other, each event was weighted according to the number of events detected in the cluster. Using this procedure, we ensure that heat maps are not biased by the number of events contained in each cluster.
3- The surface of the reference cell was segmented into 96 pixels, 6 in the direction of the short axis and 16 for the long axis. The number of clusters detected in each pixel is color-coded according to the color bar (right). The scale on the color bar is proportional to the relative average number of clusters detected in each pixel. The scale changes according to the number of cells analyzed and the total number of clusters detected.

In order to facilitate visualization, a white line was added to outline the contour the reference cell wall.

Supplementary Table 1. Equipment parts

**Parts of the PALM setup**	**Vendor**	**Specification / Part number**
Lasers 405nm – 641nm	Vortran Technology	Stradus™ 405-100<br>Stradus™ 642-100
Laser 488nm	Coherent, Inc	Cube-488-50C
Laser 532nm	Laser Quantum	Gem532
Laser 1064nm	IPG Photonics Laser	YLM5-1064-LP
Objective **OBJ**	Nikon	Plan Apo VC 100x H
Piezo stage **PZ**	Mad City Labs, Inc	Nano-drive® 1
Stage	Physik Instrumente	PI Mercury™ MS163E
Camera **C1**	Andor	iXon 897
Camera **C2**	Cooke	Pixelfly
Dichroic mirrors **DM1**	Semrock	427 nm LaserMUX™<br>503 nm LaserMUX™<br>552 nm LaserMUX™
Dichroic mirrors **DM2** and **DM3**	Chroma	zt/405/488/532/633rpc<br>z1064rdc-sp
Filter wheel **FW**	Thorlabs	FW102C
Filters	Chroma	ET525/50m<br>ET605/70m<br>ET700/75m
Data Acquisition DAQ	National Instruments	NI-USB 62-11

Supplementary Table 2. Products and consumables used for the PALM experiments

Consumables for PALM experiments	Vendor	Specification / Part number
Glass slide	Dutsher	SuperFrost ULTRA PLUS 25x75mm
Coverslip	Bellco<br>Bellco<br>Marienfeld	25mm round #1<br>22x60mm #1<br>24x60mm #1.5H
Fluorescent beads	Invitrogen	40nm TransFluoSpheres® 488/645nm or 488/605nm
Membrane dye	Invitrogen	FM® 4-64
DNA marker	Invitrogen	SYTOX® Green

Supplementary Table 3. Bacterial strains used in this work

Strain name	Genotype	Source / Reference
PY79	Wild type	Yougman et al. 1984
EB1407	*spoIIIE-eosFP (kan)*	Becker, E., this work, derived from pEB410 [4]
EB1384	*spoIIIE-gFP (kan)*	Fleming et al. 2010 [4]
pBaSysBioII	monomeric GFP	Botella et al., 2010
BTM177	spoIIIE-mMaple (kan)	This work
BTM200	*spoIIIE-gfp (kan), amyE::Pspac-ftsZ-mCherry (spec) (cat)*	This work

Bibliography

1. Sterlini JM, Mandelstam J (1969) Commitment to sporulation in Bacillus subtilis and its relationship to development of actinomycin resistance. Biochem J 113: 29-37.
2. Ptacin JL, Nollmann M, Becker EC, Cozzarelli NR, Pogliano K, et al. (2008) Sequence-directed DNA export guides chromosome translocation during sporulation in Bacillus subtilis. Nat Struct Mol Biol 15: 485-493.
3. Cormack BP, Valdivia RH, Falkow S (1996) FACS-optimized mutants of the green fluorescent protein (GFP). Gene 173: 33-38.
4. Fleming TC, Shin JY, Lee SH, Becker E, Huang KC, et al. (2010) Dynamic SpoIIIE assembly mediates septal membrane fission during Bacillus subtilis sporulation. Genes Dev 24: 1160-1172.
5. Gibson DG, Young L, Chuang RY, Venter JC, Hutchison CA, 3rd, et al. (2009) Enzymatic assembly of DNA molecules up to several hundred kilobases. Nat Methods 6: 343-345.
6. Serge A, Bertaux N, Rigneault H, Marguet D (2008) Dynamic multiple-target tracing to probe spatiotemporal cartography of cell membranes. Nat Methods 5: 687-694.
7. Sliusarenko O, Heinritz J, Emonet T, Jacobs-Wagner C (2011) High-throughput, subpixel precision analysis of bacterial morphogenesis and intracellular spatio-temporal dynamics. Mol Microbiol 80: 612-627.
8. Digman MA, Dalal R, Horwitz AF, Gratton E (2008) Mapping the number of molecules and brightness in the laser scanning microscope. Biophys J 94: 2320-2332.
