# Supplementary material for: Recruitment, Assembly, and Molecular Architecture of the SpoIIIE DNA Pump Revealed by Superresolution Microscopy
Source: PLoS Biol. 2013 May 7;11(5):e1001557. doi: 10.1371/journal.pbio.1001557 (PMC3646729; doi:10.1371/journal.pbio.1001557)
Supplement: Table S1 — Equipment parts. (DOC) [file pbio.1001557.s018.doc]

### Supplementary Table 1. Equipment parts

| Parts of the PALM setup | Vendor | Specification / Part number |
| --- | --- | --- |
| Lasers 405nm – 641nm | Vortran Technology | StradusTM 405-100  StradusTM 642-100 |
| Laser 488nm | Coherent, Inc | Cube-488-50C |
| Laser 532nm | Laser Quantum | Gem532 |
| Laser 1064nm | IPG Photonics Laser | YLM5-1064-LP |
| Objective OBJ | Nikon | Plan Apo VC 100x H |
| Piezo stage PZ | Mad City Labs, Inc | Nano-drive® 1 |
| Stage | Physik Instrumente | PI Mercury™ MS163E |
| Camera C1 | Andor | iXon 897 |
| Camera C2 | Cooke | Pixelfly |
| Dichroic mirrors DM1 | Semrock | 427 nm LaserMUX™  503 nm LaserMUX™  552 nm LaserMUX™ |
| Dichroic mirrors DM2 and DM3 | Chroma | zt/405/488/532/633rpc  z1064rdc-sp |
| Filter wheel FW | Thorlabs | FW102C |
| Filters | Chroma | ET525/50m  ET605/70m  ET700/75m |
| Data Acquisition DAQ | National Instruments | NI-USB 62-11 |
