# Supplementary material for: Recruitment, Assembly, and Molecular Architecture of the SpoIIIE DNA Pump Revealed by Superresolution Microscopy
Source: PLoS Biol. 2013 May 7;11(5):e1001557. doi: 10.1371/journal.pbio.1001557 (PMC3646729; doi:10.1371/journal.pbio.1001557)
Supplement: Table S2 — Products and consumables used for the PALM experiments. (DOC) [file pbio.1001557.s019.doc]

### Supplementary Table 2. Products and consumables used for the PALM experiments

| Consumables for PALM experiments | Vendor | Specification / Part number |
| --- | --- | --- |
| Glass slide | Dutsher | SuperFrost ULTRA PLUS  25x75mm |
| Coverslip | Bellco  Bellco  Marienfeld | 25mm round #1  22x60mm #1  24x60mm #1.5H |
| Fluorescent beads | Invitrogen | 40nm TransFluoSpheres®  488/645nm or 488/605nm |
| Membrane dye | Invitrogen | FM® 4-64 |
| DNA marker | Invitrogen | SYTOX® Green |
