# Supplementary material for: Recruitment, Assembly, and Molecular Architecture of the SpoIIIE DNA Pump Revealed by Superresolution Microscopy
Source: PLoS Biol. 2013 May 7;11(5):e1001557. doi: 10.1371/journal.pbio.1001557 (PMC3646729; doi:10.1371/journal.pbio.1001557)
Supplement: Table S3 — Bacterial strains used in this work. (DOC) [file pbio.1001557.s020.doc]

### Supplementary Table 3. Bacterial strains used in this work

| Strain name | Genotype | Source / Reference |
| --- | --- | --- |
| PY79 | Wild type | Yougman et al. 1984 |
| EB1407 | spoIIIE-eosFP (kan) | Becker, E., this work, derived from pEB410 (Fleming, Shin et al. 2010) |
| EB1384 | spoIIIE-gFP (kan) | Fleming et al. 2010 (Fleming, Shin et al. 2010) |
| pBaSysBioII | monomeric GFP | Botella et al., 2010 |
| BTM177 | spoIIIE-mMaple (kan) | This work |
| BTM200 | spoIIIE-gfp (kan), amyE::Pspac-ftsZ-mCherry (spec) (cat) | This work |
